# Online interventions for reducing hate speech and cyberhate: A systematic review

**DOI:** 10.1002/cl2.1243

**Published:** 2022-05-25

**Authors:** Steven Windisch, Susann Wiedlitzka, Ajima Olaghere, Elizabeth Jenaway

**Affiliations:** ^1^ Department of Criminal Justice Temple University Philadelphia Pennsylvania USA; ^2^ School of Social Sciences The University of Auckland Auckland New Zealand

## Abstract

**Background:**

The unique feature of the Internet is that individual negative attitudes toward minoritized and racialized groups and more extreme, hateful ideologies can find their way onto specific platforms and instantly connect people sharing similar prejudices. The enormous frequency of hate speech/cyberhate within online environments creates a sense of normalcy about hatred and the potential for acts of intergroup violence or political radicalization. While there is some evidence of effective interventions to counter hate speech through television, radio, youth conferences, and text messaging campaigns, interventions for online hate speech have only recently emerged.

**Objectives:**

This review aimed to assess the effects of online interventions to reduce online hate speech/cyberhate.

**Search Methods:**

We systematically searched 2 database aggregators, 36 individual databases, 6 individual journals, and 34 websites, as well as bibliographies of published reviews of related literature, and scrutiny of annotated bibliographies of related literature.

**Inclusion Criteria:**

We included randomized and rigorous quasi‐experimental studies of online hate speech/cyberhate interventions that measured the creation and/or consumption of hateful content online and included a control group. Eligible populations included youth (10–17 years) and adult (18+ years) participants of any racial/ethnic background, religious affiliation, gender identity, sexual orientation, nationality, or citizenship status.

**Data Collection and Analysis:**

The systematic search covered January 1, 1990 to December 31, 2020, with searches conducted between August 19, 2020 and December 31, 2020, and supplementary searches undertaken between March 17 and 24, 2022. We coded characteristics of the intervention, sample, outcomes, and research methods. We extracted quantitative findings in the form of a standardized mean difference effect size. We computed a meta‐analysis on two independent effect sizes.

**Main Results:**

Two studies were included in the meta‐analysis, one of which had three treatment arms. For the purposes of the meta‐analysis we chose the treatment arm from the Álvarez‐Benjumea and Winter (2018) study that most closely aligned with the treatment condition in the Bodine‐Baron et al. (2020) study. However, we also present additional single effect sizes for the other treatment arms from the Álvarez‐Benjumea and Winter (2018) study. Both studies evaluated the effectiveness of an online intervention for reducing online hate speech/cyberhate. The Bodine‐Baron et al. (2020) study had a sample size of 1570 subjects, while the Álvarez‐Benjumea and Winter (2018) study had a sample size of 1469 tweets (nested in 180 subjects). The mean effect was small (*g *= −0.134, 95% confidence interval [−0.321, −0.054]). Each study was assessed for risk of bias on the following domains: randomization process, deviations from intended interventions, missing outcome data, measurement of the outcome, and selection of the reported results. Both studies were rated as “low risk” on the randomization process, deviations from intended interventions, and measurement of the outcome domains. We assessed the Bodine‐Baron et al. (2020) study as “some” risk of bias regarding missing outcome data and “high risk” for selective outcome reporting bias. The Álvarez‐Benjumea and Winter (2018) study was rated as “some concern” for the selective outcome reporting bias domain.

**Authors' Conclusions:**

The evidence is insufficient to determine the effectiveness of online hate speech/cyberhate interventions for reducing the creation and/or consumption of hateful content online. Gaps in the evaluation literature include the lack of experimental (random assignment) and quasi‐experimental evaluations of online hate speech/cyberhate interventions, addressing the creation and/or consumption of hate speech as opposed to the accuracy of detection/classification software, and assessing heterogeneity among subjects by including both extremist and non‐extremist individuals in future intervention studies. We provide suggestions for how future research on online hate speech/cyberhate interventions can fill these gaps moving forward.

## 1 PLAIN LANGUAGE SUMMARY

### 1.1 Limited evidence on online interventions to reduce online hate speech and cyberhate

Not enough evidence exists to determine the efficacy of online hate speech/cyberhate interventions for reducing the creation and/or consumption of hateful content online.

### 1.2 What is this review about?

The availability of the Internet has made it a valuable setting for sharing hateful content. Therefore, it is vital to have online interventions in place that address such hateful online behavior. Unfortunately, while television and radio interventions offer some evidence about the value of these efforts to combat hateful content, online interventions lack thorough testing.

### 1.3 What studies are included?

Although our systematic review identified 22 promising reports, only 2 studies were eligible for a meta‐analysis.

### 1.4 What is the aim of this review?

This systematic review examines the success of online hate speech/cyberhate interventions for reducing hateful content online.

### 1.5 What are the main findings of this review?

There is insufficient evidence to determine the effectiveness of online hate speech/cyberhate interventions.

### 1.6 What do the findings of this review mean?

More research is needed to determine the success of online interventions for reducing online hate speech/cyberhate.

### 1.7 How up to date is this review?

The review authors searched for studies conducted between January 1990 and December 2020 and completed the initial systematic search in December 2020 and an additional search in March 2022.

## 2 BACKGROUND

### 2.1 Description of the condition

The Internet has become an everyday tool to communicate and network with people around the globe. However, its perceived anonymity, availability, and instant access have made it an environment conducive to spreading hateful content and connecting to like‐minded individuals with similar hateful ideologies. Hate speech and other prejudice‐motivated behavior, however, need to be considered on a continuum of victimization, and “like other social processes, [be seen as] dynamic and in a state of constant movement and change, rather than static and fixed” (Bowling, 1993, p. 238). It is a social process that is marked by multiple, repeat, and constant victimization (Bowling, 1993), with victims no longer distinguishing between specific hateful events and rather normalizing experiences of hateful conduct “as an everyday, unwanted but the routine reality of being ‘different’” (Chakraborti, 2016, p. 581). Understanding hateful behavior and victimization as a process allows us to connect ‘low‐level' incidents of hateful behavior to the more severe and life‐threatening incidents at the end of the spectrum (Bowling & Phillips, 2002). We often encounter such ‘low‐level' instances of hateful behavior online while browsing through, for example, Twitter and Facebook feeds. However, we have also seen instances where online hate speech/cyberhate has escalated to “real life” attacks, leaving the online sphere and spilling into the offline world (e.g., the Christchurch attack in New Zealand, the Poway Synagogue shooting in San Diego, the El Paso shooting in Texas, and their link to hateful communication on the online platform 8chan in 2019). As per Allport's (1954) scale of prejudice, more extreme forms of prejudice‐motivated violence are founded on ‘lower level' acts of prejudice and bias. Therefore, hateful content online should not be ignored.

Allport's (1954) scale of prejudice is the basis for this systematic review. Early on, Allport (1954) asserted that individuals with negative attitudes toward groups are likely to act out on these prejudices “somehow, somewhere” (p. 14) and that the more intense such negative attitudes are, the more hostile the action will be. Allport (1954) put forward a scale of acts of prejudice to illustrate different degrees of acting out harmful attitudes, which starts with *antilocution* (or what we call hate speech), described as explicitly expressing prejudices through negative verbal remarks to either friends or strangers. *Avoidance* is the next level on the scale of prejudice, with people avoiding members of certain groups, followed by *discrimination*, where distinctions are made between people based on prejudices, which leads to the active exclusion of members from specific groups (Allport, 1954). This level of acting on prejudices is routed in institutional or systemic prejudices, such as the differential treatment of people within employment or education practices as well as within the criminal justice system or through the social exclusion of certain minoritized group members. *Physical attack* is the next level on the scale of prejudice, which includes violence against members of certain groups by physically acting on negative attitudes or prejudices. The last level, *extermination*, includes ultimate acts of violence against members of specific groups, an expression of prejudice that systematically eradicates an entire group of people (e.g., genocide).

Allport's (1954) scale of prejudice makes it clear how hate speech/cyberhate is connected to more extreme forms of violence motivated by specific biases, with hate speech (or antilocution) being only the starting point (Bilewicz & Soral, 2020). The importance of this scale of prejudice is not only that it clearly illustrates a range of different ways and intensity levels to act out prejudices, but also the “progression from verbal aggression to physical violence or, in other words, the performative potential of hate speech” (Kopytowska & Baider, 2017, p. 138). This is where interventions at the lower level of prejudices, specifically online interventions targeting online hate speech/cyberhate, become important.

Because different countries inconsistently conceptualize the same hate speech phenomenon, there is no universal definition of hateful conduct online. This, unfortunately, affects our ability to develop a comprehensive search of the literature. However, there is some consensus that hate speech targets disadvantaged social groups (Jacobs & Potter, 1998). Bakalis (2018) more narrowly defines cyberhate as “any use of technology to express hatred toward a person or persons because of a protected characteristic—namely race, religion, gender, sexual orientation, disability and transgender identity” (p. 87). Another definition that also points out the ambiguity and challenges involved with identifying more subtle forms of hate speech, and also makes reference to the potential threat of hate speech escalating to offline violence, is put forward by Fortuna and Nunes (2018): “Hate speech is any language that attacks or diminishes, that incites violence or hate against groups, based on specific characteristics such as physical appearance, religion, descent, national or ethnic origin, sexual orientation, gender identity or other, and it can occur with different linguistic styles, even in subtle forms or when humor is used” (p. 5).

In this systematic review, we distinguish hate speech/cyberhate specifically from other forms of harmful online activity, such as cyber‐bullying, harassment, trolling, or flaming, as perpetrators of such online behavior repeatedly and systematically target specific individuals to cause distress, to seek out negative reactions, or to create discord on the Internet. Research focused on desensitization suggests that being exposed to hate speech leads to a normalization of prejudiced attitudes, which further leads to an increase in outgroup bias toward groups targeted by such speech (Soral et al., 2018). With society increasingly recognizing that it is inappropriate to express prejudices in public settings, interventions may include some form of social norm nudging to reduce such prejudices or interventions that “nudge behavior in the desired direction” (Titley et al., 2014, p. 60). Therefore, hate speech not only affects minoritized group members but also has an influence on the opinions of majority group members (Soral et al., 2018), which makes strategies that can elicit change in people's prejudice‐related attitudes crucial (see, e.g., Zitek & Hebl, 2007).

We specifically choose to assess the effectiveness of online hate speech/cyber hate interventions for two reasons. First, the unique feature of the Internet is that such individual negative attitudes toward minoritized groups and more extreme, hateful ideology can find their way onto certain platforms and can instantly connect people sharing similar prejudices. By closing the social and spatial distance, the Internet creates a form of collective identity (Perry, 2000) and can convince individuals with even the most extreme ideologies that others out there share their views (Gerstenfeld et al., 2003). In addition, the enormous frequency of hate speech/cyberhate within online environments creates a sense of normativity to hatred and the potential for acts of intergroup violence or political radicalization (Bilewicz & Soral, 2020, p. 9). Seeing other people post prejudiced comments online can lead to the adoption of an online group's biases and can influence an individual's own perceptions and feelings toward the targeted, stigmatized group (Hsueh et al., 2015). Second, in contrast, hate speech/cyberhate is more general and does not necessarily target a specific individual (Al‐Hassan & Al‐Dossari, 2019). Instead, hate speech/cyberhate heavily features prejudice, bias, and intolerance toward certain groups within society, with most hate speech happening online. Interventions that take place online are therefore an important way to challenge prejudice and bias, potentially reaching masses of people across the globe.

It is important to challenge hate speech, especially since hate movements have increasingly crossed into the mainstream (Futrell & Simi, 2017). With hate speech/cyberhate posing a threat to the social order by violating social norms (Soral et al., 2018), perceptions of social norms as either supporting or opposing prejudice have been found to have an influence on how individuals react online (Hsueh et al., 2015). Governments around the world face increased demand for understanding and countering hateful ideology and violent extremism both online and offline (e.g., the Christchurch Call). The US Government's 2021 national strategy for countering domestic terrorism highlights the importance of ongoing research and analysis, the sharing of knowledge and best practices internationally, and the countering of hateful ideologies and propaganda. The goal of this systematic review is to examine the effectiveness of online campaigns and strategies for reducing online hate speech and cyberhate. In doing so, we take a step toward better understanding the complex and multifaceted nature of this type of hateful messaging.

### 2.2 Description of the intervention

The Internet provides an opportunity to reach masses of people, people who are exposed to hateful content and hateful ideology online, but also people who engage in consuming and spreading hateful content online. Online interventions that address such hateful online behavior, therefore, become crucial. This systematic review set out to focus on online interventions addressing online hate speech and cyberhate, with interest in interventions deployed on websites, text messaging applications, and online and social media platforms including, but not limited to, Facebook, Instagram, TikTok, WhatsApp, Google, YouTube, and Snapchat. We focused specifically on online interventions that aimed to change people's online behavior and encouraged individuals or groups to conform to established social norms. Such social norms, for example, can be communicated through creating community standards on online platforms themselves (e.g., Facebook, Twitter, etc.), through more formal online training courses, or through anti‐hate speech/anti‐cyberhate campaigns teaching people to recognize hate, embrace diversity, and stand up to bias. Such prevention campaigns are designed to challenge bias and build ally behaviors by supplying people with constructive responses to combat, for example, antisemitism, racism, and homophobia, as well as provide resources to help people explore and critically reflect on current events. Other interventions we set out to find in this systematic review addressed online hate speech/cyberhate by adding messages to hateful online comments, countering hateful content or extremist ideology, or redirecting people to more credible sources.

### 2.3 How the intervention might work

Regardless of how an individual develops certain racial, religious, or sexual biases, in this systematic review, we were interested in online interventions that targeted and reduced the consumption and creation of original hateful content, such as spreading antisemitic Tweets and/or homophobic blog posts as well as accessing and consuming hate speech material online (e.g., watching or reading hate speech videos or blogs). For example, Bodine‐Baron et al. (2020) used rather broad messaging approaches by promoting racial sensitivity and inclusion through hashtag campaigns (i.e., “#CapekGakSih” (“Aren't You Tired?”) or “#AkuTemanmu” (“I Am Your Friend”)) on Facebook, Instagram, Twitter, and YouTube. These campaigns were designed to recast online encounters as opportunities for personal growth and share humanity. These campaigns disputed and contradicted negative stereotypes associated with specific cultures, people, and institutions by sharing different points of view based on human rights values such as openness, respect for difference, freedom, and equality. Moreover, such interventions involved blanket bans on specific behaviors enforced through the public promotion of norms or individual sanctions enforced by moderators.

Other interventions, such as the “Redirect Method,” are narrower in their messaging. These interventions generate curated playlists and collections of authentic content that challenge hate speech/cyberhate narratives and propaganda (Helmus & Klein, 2018). For instance, people who are directly searching for extremist content online may be linked to videos and written content that confronts such claims. These videos are designed to be objective in appearance instead of containing material that explicitly counters extremist propaganda. The underlying goal of this type of intervention is to provide credible content that effectively undermines extremist messaging but does not overtly attack the source of propaganda. There were three key findings associated with the Redirect Method (Helmus & Klein, 2018). First, the Redirect Method reached a portion of the “low‐prevalence, high‐risk” audience that advertising services are not designed to reach. Second, it created friction between search queries for white supremacist and/or neo‐Nazi communities and positive search results; and finally, it functioned as the conduit between high‐risk individuals and their respective delivery partners (e.g., Life After Hate) such that some passive searches became active conversations. With that said, more effort should be given to expanding the keyword list and creating partner microsites with content specifically tailored to the needs of the redirected individuals. Online platforms, such as Twitter and Facebook, have started to employ such methods, redirecting people who comment on or share “fake news” or conspiracy theories, which often are fraught with prejudicial undertones and are harmful to minoritized groups, to more credible content and news sources.

### 2.4 Why it is important to do this review

Findings from this systematic review enhance our understanding of the effectiveness of online anti‐hate speech/anti‐cyberhate interventions, help ensure that programming funds are dedicated to the most effective efforts and play a critical role in helping individual programs improve the quality‐of‐service provisions. Our findings also inform governments and policymakers of the current state of such online efforts, what works and which modes of interventions to implement, and help guide economically viable investments in nation‐state security.

Our search of the scholarly literature identified one review, Blaya (2019), as similar to the current topic. Blaya's (2019) review, however, focused on the prevalence, type, and characteristics of existing interventions for counteracting cyberhate and did not include a meta‐analysis. Two other similar reviews focused on exposure to extremist online content (Hassan et al., 2018) and communication channels associated with cyber‐racism (Bliuc et al., 2018). A search of the Campbell Library using key terms (hate OR radical*) identified two protocols and one review for further inspection to assess potential overlap. The protocols include “Psychosocial processes and intervention strategies behind Islamist deradicalization: A scoping review” by de Carvalho and colleagues (2019) and “Police programs that seek to increase community connectedness for reducing violent extremism behavior, attitudes and beliefs” by Mazerolle and colleagues (2020). A further review on a similar topic is a recently completed Campbell review (January 2020), “Counter‐narratives for the prevention of violent radicalization: A systematic review of targeted interventions” by Carthy et al. (2018) at the National University of Ireland, Galway.

Our review is distinguished from the de Carvalho and colleagues' (2019) review in that we are focusing on hate speech and cyberhate generally without delimiting our approach to a specific type of radicalization (e.g., Islamist). Furthermore, we elected to complete a systematic review and meta‐analysis. Likewise, the protocol by Mazerolle and colleagues (2020) focuses on interventions involving police officers either as initiators, recipients, or implementers of community connectedness interventions. Our review focuses specifically on any online intervention, which may or may not involve the police, but police will not be the focus nor the basis of the online intervention strategy. Judging from Carthy et al.'s (2018) protocol, our review also captured counter‐narrative interventions but differed based on setting, timing, and scope of interventions. Specifically, we were interested in online interventions that extend beyond counter‐messaging campaigns to include a broad array of interventions outlined above and extend beyond radicalization to include everyday hate and prejudice. In addition to conducting a meta‐analysis, our review builds on Blaya's (2019) work by expanding the population parameters to include both adolescents as well as adults. Blaya (2019) limited her search to include interventions aimed toward children and adolescents (e.g., young adults, teenagers) and did not focus on extremism.

## 3 OBJECTIVES

The main objective of this review was to synthesize the available evidence on the effectiveness of online interventions aimed at reducing the creation and/or consumption of online hate speech/cyberhate material. We initially sought to examine differences in intervention effectiveness based on the type of intervention and individual characteristics. However, we were unable to complete these analyses. Later in this review, we provide explanations for why we are currently unable to answer RQ 2 and RQ 3 below.

RQ 2: How is effectiveness related to the type of online hate speech/cyberhate intervention used?

RQ 3: How is effectiveness related to the characteristics of individuals experiencing the online hate speech/cyberhate intervention (e.g., age, gender, race/ethnicity, offense history, childhood trauma)?

Within this review, we, therefore, set out to investigate the following research question:

RQ 1: To what extent are online interventions effective in reducing online hate speech/cyberhate?

## 4 METHODS

### 4.1 Criteria for considering studies for this review

#### 4.1.1 Types of studies

As set out within our protocol (see Windisch et al., 2021), we planned to include both experimental and quasi‐experimental quantitative studies in this review as these methodological approaches are the most effective strategies for isolating the effect of the intervention. Therefore, eligible quantitative study designs included the following:

##### 4.1.1.1 Experimental designs

Eligible experimental designs that involved random assignment of participants to distinct treatment and control group(s). Designs that involved quasi‐random assignment of participants, such as alternate case assignment, were also eligible and were coded as experimental designs.

##### 4.1.1.2 Quasi‐experimental designs

All eligible quasi‐experimental designs must have included participants in a control condition compared to participants in a treatment condition. Eligible studies included those that report matching procedures (individual‐ or group‐level) and statistical procedures employed to achieve equivalency between groups. Statistical procedures included, but were not limited to, propensity score matching, regression analysis, and analysis‐of‐covariance. Furthermore, in anticipation of a limited quantitative evidence base, we also included quasi‐experimental studies with unmatched comparison groups that provide a baseline assessment of outcomes for both groups. Finally, time‐series analyses were also included. Eligible time‐series designs included short‐interrupted time series designs with a control group (less than 25 pre/post observations) and long‐interrupted time series designs with or without a control group (more than 25 pre/post observations). Ineligible quasi‐experimental designs involved studies that included a comparison group consisting of participants who either refused to participate in the study or who initially participated in a study but then dropped out before the start of a study.

Eligible comparison conditions included other online interventions or conditions in which participants did not receive or experience an online intervention.

#### 4.1.2 Types of participants

Both youth and adult participants of any racial/ethnic background, religious affiliation, gender identity, sexual orientation, nationality, or citizenship status were eligible for this review. The eligible youth population included study participants with a minimum age of 10 through age 17. The eligible adult population included study participants with a minimum age of 18 and older.

Studies in which only a subset of the sample was eligible for inclusion—for example, if a study subject participated in both online and offline hate speech interventions—were excluded. This exclusion was necessary to specifically focus our review on the effects of online interventions on changes in hate speech behavior online, especially when unable to extract data unique to the online subset. We did not anticipate excluding studies based on sample eligibility, as our inclusion criteria were wide‐ranging, and we took reasonable steps to locate studies that only involved online interventions.

#### 4.1.3 Types of interventions

We adopted Blaya's (2019) four‐part typology of intervention strategies to outline the potential universe of eligible interventions. The first intervention strategy is the adaptation of legal responses to hate speech/cyberhate, which includes the countering of violent extremism and aims to address cybercrime. More specifically, online interventions that are eligible range from disrupting hateful content online via specific “crackdowns” (e.g., server shutdowns, deletion of social media accounts) to responding to online hate using targeted strategies (e.g., through counter‐narratives, modifying hateful content). Examples of studies focusing on online crackdowns include the monitoring and investigation of online accounts and content takedowns, online content monitoring and censorship (Álvarez‐Benjumea & Winter, 2018), modifying hateful online comments to non‐hateful comments (Salminen et al., 2018), and possibly changing algorithms to divert users out of online echo chambers. We were also interested in interventions such as the recent take‐down of 8chan after this online platform was linked to “in real life” attacks in New Zealand and the United States and the existence of interventions that disrupt further hateful online content and radicalization after similar trigger events.

Disrupting hateful content online via such crackdowns has brought up free speech concerns, as well as concerns around online users and hateful groups just moving on to other online platforms. Responding to hateful content online using targeted strategies has, therefore, been suggested as an effective online intervention. Examples include message priming using the endorsement from religious elites (Siegel & Badaan, 2020), the use of bots to sanction online harassers (Munger, 2017), automatically generating responses to intervene in online conversations where hate speech has been detected (Qian et al., 2019), and redirecting online users to YouTube videos debunking, for example, ISIS recruiting themes (https://redirectmethod.org/). Our systematic review included a broad range of online interventions, many of which have only recently emerged.

Two other strategies identified by Blaya (2019) are the automatic identification and regulation of hate speech/cyberhate using technology, as well as the creation of online counter‐spaces and counter‐communication initiatives. These interventions include online counter‐narrative marketing campaigns, the establishment and/or use of online counter spaces, online education‐based interventions, online citizenship training, and online legislative initiatives narrowly defined to address extremist ideologies and hate speech that incites targeted violence and radicalization. In general, such interventions seek to prevent or minimize the occurrence of violent extremism or radicalization, including the spread of hate speech and extremist propaganda, by disrupting recruitment channels and creating opportunities to leave such groups.

The fourth and final intervention strategy eligible for this systematic review involves educational programs that, for example, provide people with online literacy skills and challenge racism (Blaya, 2019). We included online empowerment/resilience approaches, policy programs with an online component (e.g., Prevent and Exit programs), and educational and awareness‐raising online interventions. Such interventions may evaluate behavioral changes by individuals no longer engaging in the creation and/or consumption of cyberhate and extremist material online or may involve direct online counseling. In some instances, these online interventions may be sponsored by non‐profit and nongovernmental organizations, Internet service providers, or policy or governmental agencies in the case of legislative interventions.

#### 4.1.4 Types of outcomes

The primary outcome of interest in this systematic review was the creation and/or consumption of hateful online content. By creation, we refer to the production and authorship of original hateful content such as posting antisemitic Tweets, uploading racist YouTube videos, and/or writing homophobic blog posts (Ligon et al., 2018). The consumption of hate speech material may include visiting or being a member of a hate website/online group, watching or reading hate speech videos or blogs, being a target of online hate speech/cyberhate, or reporting hate speech material (Ligon et al., 2018). Secondary outcomes of interest in this review included affective and emotional states such as anger, fear, emotional unrest, depression, anxiety, mood swings, and attitudes toward hate speech/cyberhate. We included these secondary outcomes to also capture interventions which may not have measured behavioral changes around hateful content online, but may have otherwise impacted participants' affective and emotional states, which in turn can have an impact on the creation and/or consumption of hateful content online, and may specifically have an influence on reactions to or reporting of online hate speech/cyberhate material.

This systematic review focused specifically on *online* interventions and their impact on changes in *online* hate speech/cyberhate behavior. We, therefore, excluded offline hate behavior outcomes (i.e., hate incidents and hate crimes). As mentioned earlier, we wanted to capture online interventions that can reach masses of people across the globe and with the prospect of changing and challenging the vast amount of online (compared to offline) hate that is being seen and spread in the virtual world. In addition, it was necessary to clearly distinguish our study setting from those of previous reviews (i.e., de Carvalho et al., 2019; Mazerolle et al., 2020).

Eligible studies had to report a primary or secondary outcome (or both) to be included. There were no exclusion criteria on the source of outcome data. We had also planned to include data for the primary and secondary outcome measures from any programs, including institutional records, direct observations, surveys, or questionnaires completed by participants.

#### 4.1.5 Adverse effects

There was also the possibility of adverse effects of online interventions on online hate speech/cyberhate. We included any measure of unintended adverse effects from strategies to increase the scale of implementation of potentially effective anti‐hate speech interventions for participants, including, for example, adverse changes to emotional or psychological well‐being, defensiveness, guilt, shame, resistance to the teaching, miscommunication, creation of barriers, and dysfunctional adaptation behaviors. Adverse effects could have also included nonindividual effects such as relocation of hate speech/cyberhate to other platforms instead of a reduction of hate speech/cyberhate. We included all adverse effects described in eligible studies in this meta‐analysis.

#### 4.1.6 Other inclusion criteria

We focused on the period between 1990 and 2020. For purposes of the current study, we opted for an inclusive approach by designating 1990 as the lower end of our search period. Based on prior research, 1990 was considered a period in which the Internet transitioned to a wider infrastructure and broad‐based global community (Leiner et al., 2009). While it is conceivable that instances of hate speech or cyberhate were present online through mailing lists or emails, the odds of experimental interventions assessing the effectiveness of online interventions are slim.

Our population of studies was limited to studies published in English and German but inclusive of studies completed in any geographical region, as we focused on online content consumed and shared across geographic and nation‐state boundaries. The language parameters reflect the language abilities of the review team. Our full‐text coding captured the geographic location where studies were conducted and study participants were located.

### 4.2 Search methods for identification of studies

#### 4.2.1 Terms used to search

We conducted our systematic search between August 19, 2020 and December 31, 2020, with additional searches undertaken between March 17 and 24, 2022 based on feedback from the Campbell Collaboration Crime and Justice Coordinating Group (see Supporting Information: Appendix A and additional notes in Section 6.4). We used Zotero to manage references and implement the search strategy below. We documented the search process using the following fields: date, reviewer initials, database/website/journal searched, final search string, total yield, and notes to capture any aberrant cases (see Supporting Information: Appendix A for a complete search record). Search terms were developed based on implementation and dissemination research terminology and included search filters used in previous reviews (see, e.g., Blaya, 2019). The search strategy was conducted using the search terms specified below within the default search field of database, meaning we did not search with the Title, Abstract, Keywords (supplied by the author), and indexing terms as specified in our protocol. If and when used, these fields were used to refine searches by increasing specificity.

1. Setting search terms:
  online OR “social media” OR internet OR Twitter OR Facebook OR 8chan OR 8Kun OR Gab OR Telegram OR TikTok OR Reddit OR WhatsApp OR Instagram OR “social networking site*” OR “cybervictimization” OR “online incivility”
  AND
2. Extremism/radicalization/hate terms:
  “hate speech” OR cyberhate OR extrem* OR narrative OR racis* OR radical* OR speech OR ideolog* OR islamophobi* OR homophobi* OR transphobi* OR misogyny OR disablism OR discrim* OR terror*
  AND
3. Treatment terms:
  interven* OR option* OR strategy* OR “counter narrative*” OR “nudge” OR “norm* intervention” OR “norm* nudge” OR counternarrative* OR “alternative narrative*” OR campaign* OR counter* OR peer‐to‐peer OR prevent* OR disrupt* OR stop* OR fight* OR redirect* OR “censoring hate content”
  AND
4. Evaluation terms:
  comparison* OR quantitative OR quasi‐experiment* OR survey* OR interview* OR poll* OR mixed‐methods OR individual‐level OR group‐level OR control* OR experiment* OR study OR studies OR evaluat* OR MTurk OR longitudinal OR random* OR “digital method*” OR “machine learning” OR “natural language processing” OR multisectoral OR review*
  AND
5. Year limiter:

1990 – 2020

#### 4.2.2 Electronic searches

The search strategy described above was applied to the following databases, which cover easily accessible sources as well as gray literature. Gray literature includes reports, working papers, white papers, government documents, and generally non‐peer‐reviewed works.

*Academic databases*

EBSCOHost platform

**Table:** 

*August–December 2020*
Academic Search Complete
Communication Abstracts
Communication and Mass Media Complete
Criminal Justice Abstracts with Full Text
Education Resources Information Center (ERIC) (also searched via ProQuest)**
Military and Government Collection
PsycARTICLES
Psychology and Behavioral Sciences Collection
PsycINFO

ProQuest platform

**Table:** 

*August–December 2020*
Applied Social Sciences Index & Abstracts (ASSIA)
Criminal Justice Database
Education Resources Information Center (ERIC) (also searched via EBSCOHost)**
Gender Watch
International Bibliography of the Social Sciences (IBSS)
National Criminal Justice Reference Service (NCJRS)
Policy File Index
ProQuest Criminal Justice
ProQuest Dissertation & Theses Global
ProQuest Political Science Database
ProQuest Social Science Database
ProQuest Sociological Abstracts
ProQuest Sociology Database
Public Affairs Information Service (PAIS)
Worldwide Political Science Abstracts

Databases—Individually searched

Academic One File
Australian Federal Police Digest (AFPD)
ArticleFirst
Cambridge Journals Online
CINCH: Australian Criminology Database
Columbia International Affairs Online (CIAO)
Declassified Documents Reference System*
Don M. Gottfredson Library of Criminal Justice Gray Literature Database
European Commission
Global Issues in Context
Google Scholar
Govinfo
HeinOnline (All databases)
Homeland Security Digital Library (HSDL)
Index New Zealand: INNZ
Ingenta Connect
Journals@Ovid
JSTOR
LLMC Digital*
Multicultural Australia and Immigration Studies—Aboriginal and Torres Strait Islander Subset (MAIS‐ATSIS)
Oxford Journals Online
Oxford Scholarship Online
Project Muse
PsychiatryOnline
Sage Journals Online (which also included the following journal of interest: Sociology (Sage Full‐Text Journal Collection))
Sage Knowledge ebook collection
ScienceDirect
Scopus
Social Science Research Network
Social Sciences Citation Index (also searched via Web of Science)**
Sociological Abstracts (also searched via ProQuest)**
SpringerLink
Taylor & Francis Online (which also included the following journals of interest: Behavioral Sciences of Terrorism and Political Aggression, Critical Studies on Terrorism, Dynamics of Asymmetric Conflict, Intelligence and National Security, Studies in Conflict & Terrorism, Terrorism and Political Violence)
Web of Science (All databases)
Wiley Online Library
WorldCat

Journals

*Annual Review of Criminology*
*Annual Review of Sociology*
*Journal for Deradicalization*
*Journal of Hate Studies*
*Journal of Policing, Intelligence, and Counter‐Terrorism* (also searched via Taylor & Francis Online)**
*Perspectives on Terrorism*

Websites

Anti‐Defamation League (ADL) Combating Hate—CYBERHATE
Building Respect on the Internet by Combating Hate Speech (BRICkS)
Council of Europe
Counter Narrative Handbook*
eMORE Project—Monitoring and Reporting Online Hate Speech in Europe
European Commission against Racism and Intolerance (ECRI)—On combating hate Speech (searched via Council of Europe)
Fundamental Rights Agency
Hate Speech Watch
Home Office
Human Rights League*
IN@CH—International Network Against Cyber Hate
INHOPE*
International Network for Hate Studies online library
International Federation for Human Rights*
International League Against Racism and Anti‐Semitism (LICRA)*
Institute for the Student of Contemporary Antisemitism (iSCA)
Irish Network Against Racism
The Institute for Strategic Dialogue (ISD)
they can't—Fighting Antisemitism & Terrorism Online*
Light on Project*
MANDOLA—Monitoring and Detecting OnLine Hate Speech
Ministry of Justice (UK, New Zealand), Department of Justice (each Australian state or territory)
Online Antisemitism Taskforce*
RAND
RAND Europe
Stand Up to Hate*
The Alan Turing Institute Online Hate Research Hub
The Online Hate Prevention Institute
Together against Hate on the Net*
UNESCO—Countering Online Hate Speech
United Nations—General recommendation No. 35 (Combating racist hate speech)
Urban Institute*
VOX‐Pol
YouTube Creators for Change*

*These sources either did not yield any eligible studies or were no longer accessible.

**This search was inadvertently searched on two platforms and likely led to importing duplicates.

#### 4.2.3 Searching other resources

We completed forward citation searching and backward searches, or reference harvesting, of relevant reviews we came across in our search in addition to prior reviews and reports (e.g., Blaya, 2019; Bliuc et al., 2018; Brown & Cowls, 2015; Hassan et al., 2018; Strachan, 2014) and searched reference lists of included studies eligible from full‐text screening. In addition, we imported any article from the *Journal for Deradicalization* and the *Journal of Hate Studies* as the content from these two journals closely aligned with the review topic.

**Figure 1:**
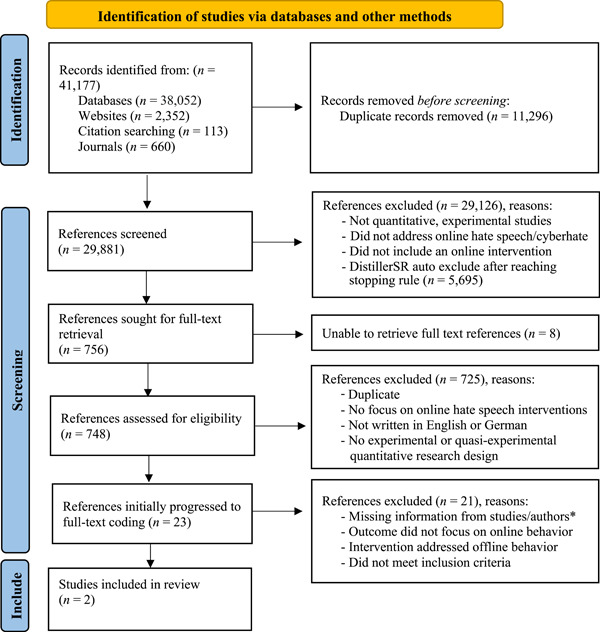
PRISMA flowchart.

We documented all search process steps in sufficient detail to ensure future replicability and correct reporting. This included a PRISMA flowchart (see Figure 1), and detailed search notes are provided in Supporting Information: Appendix A. In addition, we recorded the following information for each conducted search: the date of search, database/website searched, the final search syntax, any modifications or restrictions to the search string, the reported yield from the source, as well as the final yield of studies subsequently exported into Zotero. For database searches in particular, a reported yield would be different than what was exported to Zotero. As references were exported sequentially by results pages in a database, the final results would adjust and reflect the database algorithm removing duplicates automatically. When forward‐searching was complete, we used Google Scholar because the database identifies both published and unpublished literature.

#### 4.2.4 Criteria for determining independent findings

We anticipated two additional issues relating to independence (see Windisch et al., 2021). The first issue concerned the possibility of multiple documents reporting data from the same study/evaluation, which we would have treated as unique only if the sample did not include the same participants of any other eligible study. Related studies would have been treated as a single study. We would have chosen the study with the most information as the primary study with a coding reference to the other studies. The second issue we anticipated concerned reported outcome data at multiple time points. While we did include one study (Bodine‐Baron et al., 2020) that administered outcome surveys at 5 weeks, 10 weeks, and 15 weeks following baseline testing, it did not warrant separate analyses around the effects of the intervention because the authors only compared baseline results to the 15‐week survey. We planned to allow studies to contribute multiple effect sizes but only contribute one effect size for each outcome. If a study provided multiple effect sizes for an outcome, we planned to create one effect size by averaging the reported effect sizes for a synthesized outcome. As a sensitivity analysis, we planned to model the statistical dependencies of these effect sizes using robust variance estimation (Hedges et al., 2010). However, we identified only two effect sizes, one from each study, relevant to the meta‐analysis. Accordingly, we did not need to adjust for statistical dependency as originally planned (see Windisch et al., 2021) and did not average effect sizes nor use robust variance estimation.

### 4.3 Data and analysis

#### 4.3.1 Selection of studies

After removal of duplicates, the abstracts and titles were single screened via DistillerSR, and screeners were asked to assess the eligibility of each of the studies via the following questions:

1. Is this a quantitative or experimental study? No or Yes.
2. Does the study address online hate speech/cyberhate or online radicalization? No or Yes.
3. Does the study include an online intervention/prevention component? No or Yes.

Title and abstracts advanced to full‐text screening when researchers indicated “yes” to all questions. If screeners were unsure of the study's eligibility, these titles and abstracts were double‐screened. If these remained unsure due to limited information within the title and/or abstract, these studies were then pushed through to full‐text screening for closer investigation.

DistillerSR's “continuous AI reprioritization” feature allows for automatic learning from abstracts that screeners accepted or rejected. This meant that DistillerSR learned from our decisions on including or excluding abstracts and titles and pushed more relevant studies to the top of the screening order. This allowed for a speedier abstract and title screening process. Once studies were deemed potentially eligible at the title and abstract screening phase, we also used DistillerSR for full‐text screening via our data collection forms (see Supporting Information: Appendix B). The full texts were double‐screened. We resolved differences of opinion regarding the eligibility of a study for inclusion through discussion and consensus within team meetings. Where we were unsure about the eligibility of a study, we reached out to a subject matter expert (SME) or to the authors of the study under consideration for further information that would help us decide on its eligibility, whereby the final list of included and excluded studies was then decided.

#### 4.3.2 Data extraction and management

Two review authors independently double‐coded information from the included studies. This information was recorded in a data‐extraction form piloted before the initiation of the review. Discrepancies between reviewers regarding data extraction were resolved by consensus or, if required, via a third reviewer. Data‐extraction forms were created and hosted online using DistillerSR (see Supporting Information: Appendix B for specific coding forms). Basic information about included studies was described as a narrative and included in multiple study characteristics tables. Specifically, we described and tabulated information consistent with MECCIR reporting standards, including dates, sample size, study design, study setting, intervention characteristics, outcome characteristics, effect size data, and funding source. Where information was unavailable from published reports, we accessed online supplementary material and/or contacted study authors to obtain such data.

#### 4.3.3 Assessment of risk of bias in included studies

Two reviewers independently evaluated the risk of bias for the primary outcome using the Cochrane Risk of Bias tool, version 2.0 (Sterne et al., 2019). This tool encourages consideration of the following domains: bias in the randomization process; deviations from the intended intervention (intervention assignment); missing outcome data; bias in the measurement of the outcome; and bias in selecting the reported result.

Two review authors independently judged each source of potential bias indicating low risk, high risk, or some concerns. We then made an overall risk of bias judgment for each study by combining ratings across the six domains. Specifically, if any of the above domains were rated at high risk, the overall risk of bias judgment would be rated at high risk. Finally, we processed the “risk of bias” assessments using the revised Cochrane risk‐of‐bias tool for randomized trials (ROB 2) as well as the Cochrane Handbook and the Methodological Expectations of Campbell Collaboration Intervention Reviews (MECCIR) reporting standards. We made our risk of bias ratings available in Table 3 and Figure 4. As the authors of the original studies provided adequate details for this assessment, we did not need to contact corresponding authors for clarification. However, should this become problematic in any future review updates, we would solve disagreements through discussions with authors.

We planned to address the risk of bias in non‐randomized quantitative studies using ROBINS‐I and the domains of bias in selecting participants and all domains of bias in post‐intervention (Higgins et al., 2011; Sterne et al., 2016). We coded for the experimental and quasi‐experimental design type based on assignment (e.g., matching, waitlist control, cohort, etc.) at the study level. However, no quasi‐experimental studies fit our inclusion criteria. For future review updates, quasi‐experimental studies will be evaluated using the ROBINS‐I tool as outlined in the protocol (Windisch et al., 2021).

#### 4.3.4 Measures of treatment effect

The primary outcome for this review is content creation and consumption of online hate speech/cyberhate. The underlying nature of data for this outcome was continuous. The effect size for this review is the standardized mean difference. One included study provided proportions (Bodine‐Baron et al., 2020), so we used the logit method for transformation, dividing the transformed effect size and its standard error by 1.83, the standard deviation of the logistic distribution, to make it comparable to the standardized mean difference effect size (Lipsey & Wilson, 2001).

#### 4.3.5 Unit of analysis issues

Unit of analysis issues occurred within studies that used subjects as the unit of analysis versus studies that used comments/tweets as the unit of analysis, especially in cases where the authors did not account for nested subjects by using multilevel models (e.g., tweets nested within individuals). Our final two studies did not have any unit of analysis issues, as Bodine‐Baron et al. (2020) used subjects as their unit of analysis, and Álvarez‐Benjumea and Winter (2018) used a multi‐level model to account for tweets within subjects. For future updates of this review, our approach for handling these issues is specified in the protocol (Windisch et al., 2021).

#### 4.3.6 Dealing with missing data

Missing data can take the form of missing studies, missing outcomes, missing summary data, or missing participants. Although we took all reasonable steps to retrieve all full text documents—via Temple University Library and the University of Auckland Library, and subsequently reaching out to study authors— there were eight studies that we were unable to locate for full‐text screening (see PRISMA flowchart in Figure 1 and the list of studies awaiting classification attached later in this review). Of the twenty studies that we planned to code, six studies lacked the necessary information to allow inclusion in a meta‐analysis (see Supporting Information: Appendix E). In these situations, we contacted study authors with a request to provide the missing text, with some either not responding to our requests or responding after the period of performance (see Pigott & Polanin, 2020). We plan to follow up with these authors in subsequent review updates. With our final two studies, we did not encounter issues with missing outcomes or missing participants.

#### 4.3.7 Assessment of heterogeneity

We intended to use study design, among other factors, to explore heterogeneity between study outcomes using the *Q*‐statistic and *I* ^2^ statistics to describe the percent variation across studies (see Windisch et al., 2021). Posthoc moderating factors could have included the intervention setting, such as an online intervention versus a laboratory or classroom intervention setting. Unfortunately, we could not explore heterogeneity because we lacked viable a priori and posthoc moderators to use. We attempted to collect information on study sample characteristics (i.e., age, gender, race/ethnicity), but this information was incomplete or not reported within the final set of studies. Furthermore, both studies were randomized, and the intervention took place in an online setting.

#### 4.3.8 Assessment of reporting biases

Publication selection bias is an important consideration when assessing the robustness of meta‐analytic findings because statistically significant results are more likely than nonsignificant results to be published (Lipsey & Wilson, 2001; Rothstein et al., 2005). To minimize publication bias, we extended our search to gray literature studies and included technical reports, theses, and other unpublished works (e.g., government and agency reports) (Rothstein & Hopewell, 2009). One of the two studies included in this review is a technical report found via a gray literature website search (see Bodine‐Baron et al., 2020). Unfortunately, we could not use various methods (see Coburn & Vevea, 2015) to assess for publication bias given the limited number of included studies (*n* = 2). Nevertheless, we surmise there is a possibility of publication bias in the results given the variety of potential sources of bias, the availability of eligible studies being chief among them, in addition to language bias.

#### 4.3.9 Data synthesis

**Figure 2:**
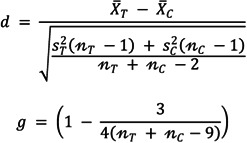
Standardized mean difference and Hedges *G* formulas.

**Figure 3:**
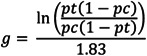
Logit transformation formula.

The underlying nature of data for this outcome was continuous. As such, we calculated the standardized mean difference for this review, using the Stata *meta set* command for precomputed effect sizes. We used the following formulas to compute standardized mean differences and standard errors (see Figure 2):

One included study provided proportions (Bodine‐Baron et al., 2020). We used the logit method for transformation and divided the logged odds ratio by 1.83, the standard deviation of the logistic distribution, to rescale the logged odds ratio onto the normal distribution (Lipsey & Wilson, 2001; see Figure 3).

We used a random‐effects model for the meta‐analysis, estimated using the restricted maximum likelihood (REML) method. All statistical analyses were performed using Stata IC/16.1. Per our protocol, Windisch et al. (2021), we intended to implement robust variance estimation to address statistically dependent effect sizes (correlated effects) using *robumeta* (Hedges et al., 2010). However, both studies in the meta‐analysis contributed one effect size each, for the content creation outcome, so we did not employ this method. Furthermore, we did not have any meaningful moderators to exploit the benefits of robust variance estimation fully. Therefore, additional research is needed for all treatment types, and the outcome examined in this meta‐analysis and other outcomes of interest that we were unable to meta‐analyze within this systematic review (see Windisch et al., 2021).

#### 4.3.10 Subgroup analysis and investigation of heterogeneity

Heterogeneity was assessed using *I* ^2^ in conjunction with *τ* ^2^ (tau‐squared) and *χ* ^2^ (Chi‐squared). Our protocol (Windisch et al., 2021) indicated we would split the included studies into subgroups based on study design, demographics (e.g., political affiliation, age, etc.), and intervention characteristics to explain the heterogeneity. For instance, to explore heterogeneity according to study design (whether studies are RCTs or non‐RCTs), we would have tested whether the mean effect size from the RCT‐only studies differed from the mean effect size from the non‐RCTs studies. However, we could not conduct moderator analyses on study design as both studies used random assignment without matching, and both studies were conducted online, and sample characteristics were either incomplete or not reported. Moreover, we did not have sufficient studies and effect sizes to conduct any meaningful subgroup analyses or moderator analyses.

#### 4.3.11 Sensitivity analysis

We do not have any sensitivity analyses to specify. During the review process, we did not encounter unusual issues suitable for sensitivity analyses.

#### 4.3.12 Treatment of qualitative research

This review did not synthesize any existing qualitative research.

#### 4.3.13 Differences between protocol and review

We made several decisions or deviations from the protocol at different stages of this review. In the search between August and December 2020, we accidentally searched three databases both separately and via database aggregators during our search for references. This double search applied to the Social Sciences Citation Index, which was also picked up by our search via Web of Science, and Sociological Abstracts, also searched via ProQuest. In addition, Education Resources Information Center (ERIC) was searched via both database aggregators, EBSCOHost and ProQuest. *The Journal of Policing*, *Intelligence, and Counter‐Terrorism* was also searched separately and via Taylor & Francis Online search. These double searches have likely led to importing duplicates and added to our number of records identified within the database reference total (see Figure 1, PRISMA flowchart). These duplicates, however, were later removed.

Although our protocol reflects a separate search for the following journals of interest, these were picked up within our Taylor & Francis Online search and are therefore no longer listed as separate searches within this final report: *Behavioral Sciences of Terrorism and Political Aggression*; *Critical Studies on Terrorism*; *Dynamics of Asymmetric Conflict*; *Intelligence and National Security*; *Studies in Conflict & Terrorism*; and *Terrorism and Political Violence*. We did, however, search Sage Journals Online (which also included Sociology [Sage Full‐Text Journal Collection]) and Sage Knowledge Ebook Collection separately, as it was not possible to search the Sage platform overall for these databases. These are now in alphabetical order under the individually searched databases to reflect this change in our electronic search. Two databases, Declassified Documents Reference System and LLMC Digital, were screened via the website. This decision was made for websites that did not allow for easy export into Zotero.

In addition, some websites that we had planned to search within the protocol stage either did not yield any eligible studies or were no longer accessible (see search notes in Supporting Information: Appendix A for detail). These include the Counter Narrative Handbook; Human Rights League; INHOPE; International Federation for Human Rights; International League Against Racism and Anti‐Semitism (LICRA); they can't—Fighting Antisemitism & Terrorism Online; Light on Project; Stand Up to Hate; Together against Hate on the Net; Urban Institute; and YouTube Creators for Change. The European Commission against Racism and Intolerance (ECRI)—On Combating Hate Speech is part of the Council of Europe and was picked up via our Council of Europe search. We also added two more websites to our search since the acceptance of our protocol: the Online Antisemitism Taskforce website was searched but did not yield any relevant references. However, The Institute for Strategic Dialogue (ISD) website was valuable for additional references.

Within the protocol, we proposed searching on the title, abstract, keyword, and indexing terms search fields. However, we deviated from the protocol as we searched for our search terms within all search fields. However, in some searches, we used specific search fields as filters to increase the specificity of specific searches (see search notes in Supporting Information: Appendix A for details about this process).

At the title and abstract screening stage (level 1), we deviated from the protocol by only screening for studies in English and German, as the Arabic and Persian speaker was no longer available to assist us. We also dropped the screening questions: “Is the study in English, German, Persian, or Arabic?” and “Was the study conducted between 1990 and 2020” as we realized that most references included an English title and abstract, regardless of the actual study language, and that the year of data collection or the intervention was often not noted within the title or abstract. The first question regarding whether the study was a quantitative or experimental study was a deviation from the review protocol. This question was added to the title and abstract screening stage as we encountered many studies that fit our other two screening questions but were clearly not quantitative or experimental.

We also piloted the coding forms at different stages of the review process in DistillerSR, either to make screening documents more efficient or to remove questions/fields that were deemed unnecessary and adjusted the coding forms accordingly (see Supporting Information: Appendix B).

Moreover, we initially proposed to use the analog‐to‐the‐ANOVA method for a single categorical variable to perform moderator analyses. However, given the insufficient number of studies and effect sizes, we could not conduct any meaningful moderator analyses.

Lastly, given the scarcity of experimental evaluations, we could not assess the effectiveness of online hate speech/cyberhate interventions in terms of the type of intervention used (RQ 2). We were also unable to assess the effectiveness of online interventions related to the characteristics of subjects such as age, gender, race/ethnicity, offense history, or childhood trauma (RQ 3). While both studies reported age, sex, and ethnicity/nationality, neither study assessed differences in intervention effectiveness based on these factors.

## 5 RESULTS

### 5.1 Description of studies

#### 5.1.1 Results of the search

The systematic searches conducted between August 19, 2020 and December 31, 2020, and March 17 and 24, 2022 yielded 41,177 references. We identified these references by searching across two database aggregators, 36 individual databases, six individual journals, 34 websites, and multiple forward and backward citation searches. A total of 11,296 duplicate references were removed before screening the titles and abstracts, which left 29,881 references. Following title and abstract screening, 29,126 references were excluded for varying reasons, such as being an ineligible study design, not addressing online hate speech/cyberhate, or not exploring an online intervention. This left 756 references eligible for full‐text retrieval. Of these 756 references, we could not retrieve eight documents (see studies awaiting classification reference list). We excluded a total of 725 references because they did not meet our eligibility criteria (i.e., studies that did not focus on online hate speech/cyberhate interventions, studies that did not use English or German, and studies that did not include an experimental or quasi‐experimental study design). A total of 23 references were initially considered eligible for full‐text coding. During full‐text coding, 21 of these references were excluded due to the outcome not focusing on online behavior, the intervention addressing offline behavior, or the study not meeting other inclusion criteria for this review (see Supporting Information: Appendices C and D for characteristics of excluded studies). Some of these studies had incomplete information, and we could not receive a response from study authors in time. These studies, however, were signposted via an asterisk (*) and noted for possible inclusion in an update to this review. Overall, two studies were included in the final review and deemed eligible for the meta‐analysis (refer to Figure 1 for PRISMA flow diagram illustrating the reference distillation process and see list of references included in the meta‐analysis).

#### 5.1.2 Included studies

The study characteristics for the two included studies are displayed in Table 1. One study was conducted in Asia (Bodine‐Baron et al., 2020), and the other was conducted in Europe (Álvarez‐Benjumea & Winter, 2018). While both studies were published in English, one study was administered in Indonesian, specifically Bahasa (Bodine‐Baron et al., 2020), and the other study was administered in German (Álvarez‐Benjumea & Winter, 2018). Both studies were published in the late 2010s. Moreover, the Bodine‐Baron et al. (2020) study was a technical report, whereas, Álvarez‐Benjumea and Winter's (2018) study was a peer‐reviewed journal article. The intervention was initiated by an online platform for Bodine‐Baron et al. (2020) and was researcher‐initiated for Álvarez‐Benjumea and Winter (2018). In addition, both included studies were funded by an external agency. Both studies used random assignment without matching and maintained the integrity of randomization. The inclusion of studies employing random assignment is preferred as it offers the best basis to examine the influence of online inventions in mitigating the creation and/or consumption of hateful content online and has a low risk of selection bias.

**Table 1:** Characteristics of included studies—Study level

	Bodine‐Baron et al. (2020)	Álvarez‐Benjumea and Winter (2018)
Publication type	Technical report	Journal article
Research funded by grant/external agency	Yes—Global Engagement Center at the US Department of State & the International Security and Defense Policy Center of the RAND National Security Research Division	Yes—Max Planck Society
*Study setting/context*		
Geographic location	Asia (Indonesia)	Europe (Germany)
Language (of publication)	English	English
Language (of study)	Indonesian (Bahasa)	German
Year of data collection	2019	2016
*Study design*		
Methodological approach	Quantitative	Quantitative
Sample size	1570 subjects	1469 tweets^a^ (nested in 180 subjects)
Unit of assignment	Individual	Individual
Subjects assigned to condition	Randomly without matching	Randomly without matching
Intervention type	Online only	Online only
Researcher involvement	Online platform initiated intervention	Researcher initiated intervention

^a^ According to Álvarez‐Benjumea and Winter (2018), a total of 1585 comments were collected with 116 deemed invalid/unintelligible.

A total of two hate speech content creation treatments and one comparison condition were evaluated in the meta‐analysis. We also present individual effect sizes for content creation from one study, Álvarez‐Benjumea and Winter (2018). One of the studies examined three different treatment conditions regarding the creation of comments: censored, extremely‐censored, and counter‐speech compared to one baseline condition (Álvarez‐Benjumea & Winter, 2018). This baseline condition was the comparison variable for this study. Table 2 provides an overview of the characteristics of the included intervention studies. Both studies included individuals who were at least 18 years old. While the maximum age for one study only indicated above 45 years, the maximum age of individuals participating in the second study was 35. In addition, the sex distribution for one of the studies was 67.1% male, while the other was 45% male. Bodine‐Baron et al. (2020) study had a larger sample size with 475 and 465 individuals in the treatment and comparison groups, respectively. The comparison group for this study watched media, entertainment, and Public Service Announcement (PSA) advertisements, but not a “#Search for Common Ground” campaign. Alternatively, the sample size for the comparison group of Álvarez‐Benjumea and Winter's (2018) study was 47 individuals, with the three treatment groups having sample sizes of 42, 45, and 46 individuals.

**Table 2:** Characteristics of included studies—Comparison level

	Bodine‐Baron et al. (2020)	Álvarez‐Benjumea and Winter (2018)
*Demographics*		
Age		
Youngest age in sample	18	18
Oldest age in sample	35	>45
Sex distribution	67.1% male	45% male
Ethnicity/Nationality	100% Indonesian^a^	100% German^b^
*Sample size*		
Treatment group	475	344/373/377 tweets (nested in 42/45/46)
Comparison group	465	375 (nested in 47)
*Intervention*		
Type	Social media campaign	Responding to online hate, deletion of hate content
Content	General online hate speech/cyberhate	Multiple (related to current social topics: feminism, LGBT rights, refugees and multiculturalism, and poverty)
Location	Online social media platform: Facebook, Instagram, Twitter, YouTube	Online forum resembling Internet forum
Comparison condition	Comparison exposure (control group)—advertisements and public service announcement campaigns	Comparison exposure (baseline group) – balanced mix of two friendly, two neutral, and two hostile comments
Outcome	Use social media	Frequency of uncivil comments

^a^ According to Bodine‐Baron et al. (2020), people living full‐time in Indonesia were considered Indonesian. The authors suggest that due to the low immigration rate in Indonesia, it is likely that all participants within their study were native‐born (Bodine‐Baron et al., 2020). People living outside of the 22 Indonesian provinces were not eligible for inclusion in the study, and the majority of participants within the sample (75%) were from the six provinces of Java (Bodine‐Baron et al., 2020).

^b^ According to Álvarez‐Benjumea and Winter (2018), only German residents were included in the sample.

While both studies evaluated the creation of hate speech content, the interventions differed. Specifically, Bodine‐Baron et al.'s (2020) intervention involved social media campaigns, public service announcements, and television ads designed to recast online encounters as opportunities for personal growth and share humanity. These campaigns disputed and contradicted negative stereotypes associated with specific cultures, people, and institutions by sharing different points of view based on human rights values such as openness, respect for difference, freedom, and equality. Alternatively, Álvarez‐Benjumea and Winter's (2018) intervention involved varying degrees of hate speech censoring in which forum moderators deleted participants' comments and presented participants only with friendly and neutral comments (censored condition); deleted participants' comments and presented participants only with friendly comments (extremely censored condition); and replied to hostile comments by highlighting the unacceptability of hostile opinions (counter‐speaking). More variation among intervention types is required to make meaningful conclusions regarding the effect of intervention types. The location for the intervention for Álvarez‐Benjumea and Winter's (2018) study was on an online forum resembling an Internet forum. In contrast, the location for the intervention for Bodine‐Baron et al.'s (2020) study was on the social media platforms Facebook, Instagram, Twitter, and YouTube.

The creation of online hate speech/cyberhate was the common outcome of interest for both included studies. Our systematic review did not capture any online interventions that reported on outcome measures of the consumption of hate speech material (e.g., visiting or being a member of a hate website/online group, watching or reading hate speech videos or blogs, being a target of online hate speech/cyberhate, or reporting hate speech material). Álvarez‐Benjumea and Winter (2018) rated subject comments on a 9‐point Likert scale, ranging from “friendly” to “hostile.” An example of a friendly comment included, “Very brave, I find it great and refreshing. I find despising homosexuals generally bad,” whereas an example of a hostile comment included, “Gays are the last thing I would tolerate, especially in public.” In contrast, Baron‐Bodine and colleagues (2020) assessed how participants would respond to a dispute by airing their feelings on social media. While the authors did not specify the nature of the social media posts, we elected to treat them as antisocial because participants were given five options for how they may respond to a dispute with somebody, including: 1 = “do nothing,” 2 = “talk,” 3 = “insult,” 4 = “use social media” and 5 = “use violence.” Similar to Allport's (1954) scale of prejudice, we viewed these responses as escalating from the least combative behavior (i.e., “do nothing”) to behaviors with more life‐threatening consequences (i.e., “use violence”). From this perspective, “use social media” was considered more antisocial than insulting someone but less antisocial than using violence. An example of using social media to resolve a dispute would include doxxing a person, blasting their networks with spam, or inserting @mention messages to legitimate users.

Secondary outcomes of interest in this review included affective and emotional states such as anger, fear, emotional unrest, depression, anxiety, mood swings, and attitudes toward hate speech/cyberhate. Our systematic search did not yield any eligible studies that measured these outcomes.

#### 5.1.3 Excluded studies

Due to the large number of full‐text documents already screened in our search between August and December 2020 (*n* = 748), we broadly indicate the reasons for excluding these studies. In addition, most studies at this stage were excluded due to the absence of an eligible empirical intervention (*n* = 411) or because study authors assessed the accuracy of online hate speech/cyberhate detection and classification software (*n* = 291) without testing an intervention. Due to the number of excluded studies, the “references to excluded studies” contain interventions that were initially deemed eligible but were subsequently excluded for various reasons (*n* = 21).

Out of the 748 retrieved full‐text documents, 23 progressed to the full‐text coding stage, with 21 of these references excluded during full‐text coding (see PRISMA flowchart in Figure 1 and the list later within this document), for the following reasons: six studies were excluded because the studies lacked the necessary information to complete the meta‐analysis, such as standard errors, standard deviations, confidence intervals, or sample sizes. The potential impact of these studies is unclear without this information to assess. While attempts were made to contact the corresponding author(s) of these studies, we were unsuccessful in receiving the required information in time for inclusion. Four additional references were nearly eligible but ultimately excluded because the online intervention focused more on the effects of media exposure on cognition than countering the transmission, creation, and/or consumption of online hate speech/cyberhate materials (see, e.g., Shortland et al., 2020). Finally, 11 references were excluded upon further examination because they did not meet our inclusion criteria. For example, in 5 of these studies, the online intervention addressed offline rather than online hate speech/cyberhate behaviors.

#### 5.1.4 Risk of bias in included studies

Methodological quality and risk of bias were coded during data extraction. Two reviewers independently evaluated the risk of bias using the Cochrane Collaboration's risk of bias tools (RoB 2). In particular, we focused the risk of bias assessment on the following domains: randomization process, deviations from intended interventions, missing outcome data, measurement of the outcome, and selection of the reported results (Sterne et al., 2019). Our ratings for evaluating the risk of bias were “low risk,” “some concerns,” and “high risk” of bias (see Table 3 for Summary of Risk of Bias Ratings).

**Table 3:** Risk of bias summary table

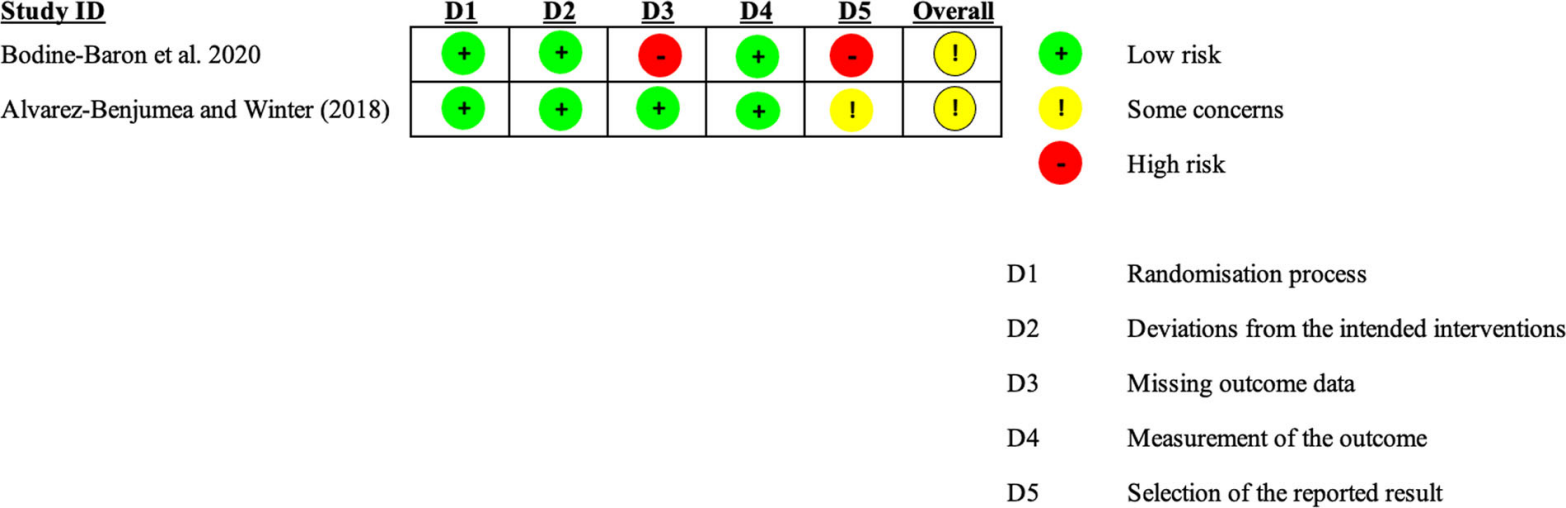

Based on our assessment, we rated both studies as “low risk” on the randomization process as researchers reported simple random assignment. We rated both studies as “low risk” on the deviations from intended interventions domain as the authors utilized a “double‐anonymous” research designed in which both the researchers and participants were unaware of the intervention conditions/manipulation. In addition, authors of both studies used appropriate analysis to estimate the effect of the intervention. Specifically, Bodine‐Baron et al. (2020) utilized OLS regression and Álvarez‐Benjumea and Winter (2018) utilized a random intercept regression model with two random factors. In terms of the measurement of the outcome domain, we rated both studies as “low risk” because researchers utilized an appropriate method for measuring the outcome, implemented procedures to minimize differences in measurements between groups (e.g., trained and validated external raters), and utilized a double‐anonymized research design, which reduced bias introduced by knowledge of the intervention group.

**Figure 4:**
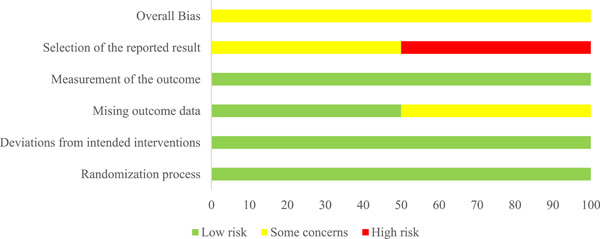
Risk of bias summary.

In terms of missing outcome data, we identified “high” risk of bias as an issue in one study (Bodine‐Baron et al., 2020) because, relative to the baseline‐only sample, follow‐up respondents were older, more likely to live in Java, more likely to have the Internet at home, and regularly used social media. The source of this difference in the sample characteristics at follow‐up relative to baseline was differential attrition, as a portion of the individuals who completed the baseline survey did not complete any of the follow‐up surveys. The Álvarez‐Benjumea and Winter (2018) study was rated as “low risk” for the missing outcome data domain as data was available for all randomized participants (see Figure 4 for Risk of Bias Summary).

As mentioned in 4.3.3, we assessed eligible studies for selective outcome reporting bias, which concerns outcome data authors may not have reported for all variables measured in their study. In this review, the Bodine‐Baron et al. (2020) study was rated “high risk” for selective outcome reporting bias. In this study, the authors collected outcome data at multiple time points—at baseline, during the intervention, and at its conclusion (5 weeks and 10 weeks, respectively), and during a follow‐up (15 weeks). However, the authors only reported baseline and 15‐week outcome data. The Álvarez‐Benjumea and Winter (2018) study was rated as “some concern” for the selective outcome reporting bias domain as the authors did not specify if the data that produced the results were analyzed following a prespecified analysis plan. This information was not included in the published manuscripts.

### 5.2 Effects of interventions

Our systematic search identified a total of two eligible studies, which allowed us to conduct one meta‐analysis, as well as a presentation of single effect sizes (see Supporting Information: Appendix F for full Stata log). Of the two eligible studies, we included two effect sizes, one from each study, in our meta‐analysis. Although the Bodine‐Baron et al. (2020) study included seven effect sizes in total, only one outcome aligned with those reported by Álvarez‐Benjumea and Winter (2018). The other six effect sizes were not related to content creation/consumption (e.g., responses to disputes that involved the following actions: doing nothing, talking, insulting someone, or using violence; and justifying violence based on religious or ethnic insults). We analyzed effect sizes from online interventions designed to reduce the creation and/or consumption of hateful online content.

Table 4 displays the mean effect size for content creation (*g* = −0.134, 95% confidence interval [CI] [−0.321, −0.054]). The effect indicates a small reduction in creating hateful content online, but is not statistically significant. The insignificant results of the meta‐analysis indicate that there was no difference in the creation of negative outputs such as writing hateful comments (Álvarez‐Benjumea & Winter, 2018) or using social media in response to disputes (Bodine‐Baron et al., 2020) when participants were exposed to the counter‐speaking treatment versus the baseline/control group condition. While there were no statistical differences between the groups, the effect size favored the intervention, whereby those in the counter‐speaking condition created lower levels of hateful content online.

**Table 4:** Mean effect size for content creation

		95% CI		
Study	Hedges' *g*	Lower	Upper	% Weight
Álvarez‐Benjumea and Winter (2018)	−0.109	−0.354	0.122	41.61
Bodine‐Baron et al. (2020)	−0.168	−0.458	0.122	58.39
Overall	−0.134	−0.321	0.054	

*Note*: Model estimated using random effects model using restricted maximum‐likelihood. *z *= −1.40, *p *= 0.161; *τ* = 0.000; *I* ^2^ (%) = 0.00; *Q* = 0.09, *df* = 1, *p* = 0.7633.

Abbreviation: CI, confidence interval.

Table 5 displays all four effect sizes across the two studies. The Álvarez‐Benjumea and Winter (2018) study also included treatments that were deemed different from counter‐speaking, namely the censoring of hateful content (i.e., the deletion of prior hateful content and presenting only friendly/neutral content) and extremely censoring of hateful content (i.e., presenting only friendly content). Table 5 indicates that the effect sizes favored the intervention, whereby those in the censored condition (*g* = −0.328) and the extremely censored condition (*g* = −0.314) created lower levels of hateful online contents. The censored and extremely censored treatments seem to create lower levels of hateful online content, compared to the two effect sizes that included a counter‐speaking treatment. However, the Bodine‐Baron et al. (2020) study did not include a treatment similar to Álvarez‐Benjumea and Winter's (2018) censoring treatment, which meant we could not include any further effect size comparisons within the meta‐analysis. For the meta‐analysis results reported above, only the one effect size from Bodine‐Baron et al. (2020) was combined with the effect size from the counter speech intervention from the Álvarez‐Benjumea and Winter (2018) study.

**Table 5:** Effect sizes for content creation

Study	Outcome	Hedges' *g*	SE	% Weight
Bodine‐Baron et al. (2020)	Social media use	−0.168	0.148	45.62
Álvarez‐Benjumea and Winter (2018)	Counter speech	−0.109	0.124	64.02
Álvarez‐Benjumea and Winter (2018)	Censored speech	−0.328	0.126	62.55
Álvarez‐Benjumea and Winter (2018)	Extremely censored speech	−0.314	0.125	63.20

While our meta‐analysis results indicate no variability between studies (*τ* ^2^ = 0.00, *Q* = 0.09, *p* = 0.763) and *I* ^2^ = 0%, suggesting variability is due to chance, only two studies were included. *Q* is traditionally underpowered when few studies are included (Altman et al., 2021; Higgins et al., 2003), which is the case for this review. Furthermore, we can presume heterogeneity is given or inevitable (see Bryan et al., 2021; Higgins et al., 2003), particularly for social science research.

**Figure 5:**
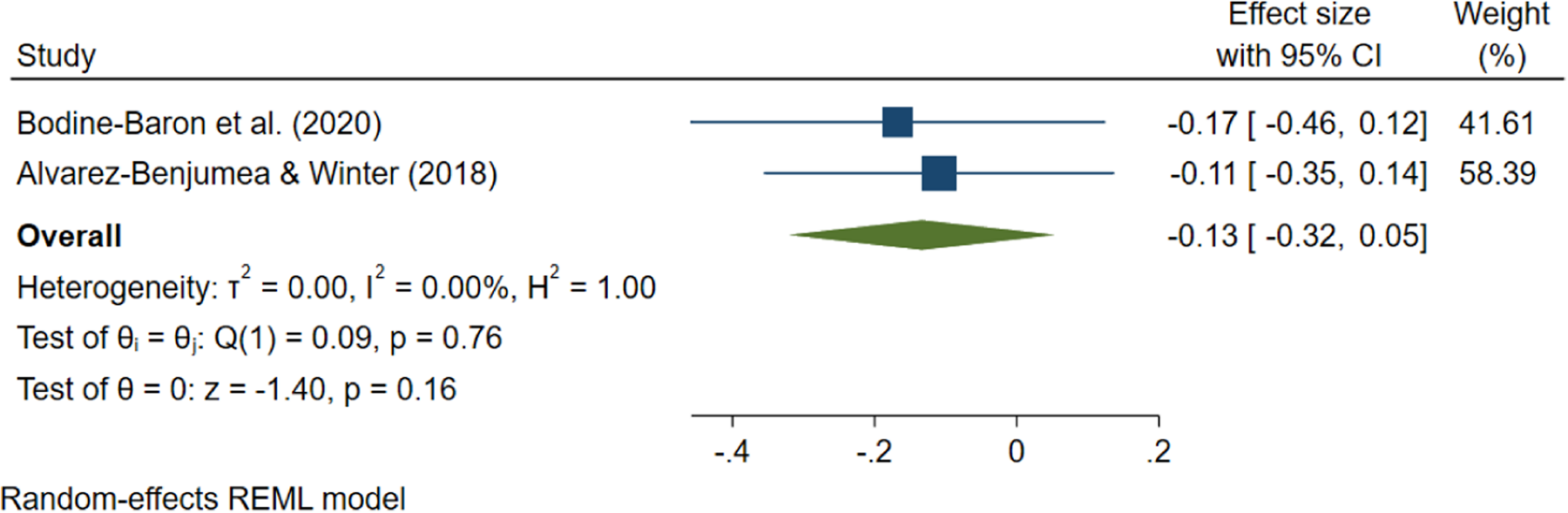
Forest plot for meta‐analysis.

These effect sizes and lack of heterogeneity should be interpreted very cautiously. Both studies measured negative behavior as higher scores and instances of online behavior (see Forest Plot in Figure 5). While at least two studies can be meta‐analyzed, we hesitate to draw any final conclusions about the effect of online interventions in reducing the creation of hateful/cyberhate content online. Therefore, replication is required.

## 6 DISCUSSION

### 6.1 Summary of main results

The main objective of this review was to synthesize the available evidence on the effectiveness of online interventions aimed at reducing the creation and/or consumption of online hate speech/cyberhate material. Our systematic search identified two studies that met our eligibility criteria (Álvarez‐Benjumea & Winter, 2018; Bodine‐Baron et al., 2020), one of which had three treatment arms (Álvarez‐Benjumea & Winter, 2018). For our meta‐analysis, we chose the treatment arm from the Álvarez‐Benjumea and Winter (2018) study that most closely aligned with the treatment condition in the Bodine‐Baron et al. (2020) study. Both studies evaluated the effectiveness of online interventions for reducing online hate speech/cyberhate. The mean effect was small (*g* = −0. 134, 95% CI [−0. 321, −0.054]) and not significant. This finding is not conclusive, with only two studies contributing to this effect and given the scarcity of high‐quality studies measuring the outcome. The evidence base does not allow for any strong conclusions regarding the effectiveness of online interventions for reducing online hate speech/cyberhate.

### 6.2 Overall completeness and applicability of the evidence

While this review offers a meta‐analysis of the effectiveness of online interventions aimed at reducing the creation and/or consumption of online hate speech/cyberhate material, it must be acknowledged that the scope and span of online interventions likely extend beyond the two studies included in this review. This is the case for three reasons.

First, while we had promising results in the initial searches of our review, most online interventions did not meet the inclusion criterion for outcomes related to online hate speech/cyberhate. Instead, the evaluative components of many of these campaigns were more reflective of the effects of media exposure on cognition than countering the transmission and/or consumption of online hate speech/cyberhate materials (e.g., Frischlich et al., 2018; Shortland et al., 2020). Moreover, 290 references were excluded from this review as they measured outcomes related to the accuracy of computer algorithms to classify and identify hateful online content rather than overall effectiveness at reducing cyberhate and online hate speech.

Second, we know that additional campaigns exist that fight online hate speech and cyberhate, however, the effectiveness of such tools for the reduction of hateful content online either still needs testing by use of an experimental study design, or such experimental studies are currently still underway and therefore not captured within the present review. For example, one intervention we encountered early on in our initial search was the Redirect Method which generates curated playlists and collections of authentic content that then challenge hate speech/cyberhate narratives and propaganda (Helmus & Klein, 2018). Although captured in our review search, the Helmus and Klein (2018) study was ineligible for inclusion in this review, as it was not a quasi‐/experimental study, and there was no assessment of the impact such video content can have on user attitudes or behavior.

Third, it is likely that additional studies exist that were not picked up in our search and screening due to the mentioned language constraints. Such language constraints may have also had a part in us only picking up studies within specific country contexts (i.e., Indonesia and Germany) with additional online interventions and studies likely existing within other country and language contexts. In addition, the two final studies included participants from the age of 18, which meant our review did not capture any studies with younger participants.

### 6.3 Quality of the evidence

There are three potential limitations concerning external validity (generalizability). First, in addition to being restricted to German (Álvarez‐Benjumea & Winter, 2018) and Indonesian (Bodine‐Baron et al., 2020) residents, we lacked complete information on the ages for both study samples. Although we have no evidence to assume that treatment effects may or may not be qualitatively changed by subjects' age, the results in this article should not be interpreted as prevalence estimates of hate speech.

Second, we identified “some” risk of bias as an issue in the Bodine‐Baron et al. (2020) study because, relative to the baseline‐only sample, follow‐up respondents were older, more likely to live in Java, more likely to have access to the Internet and be regular social media users. The Bodine‐Baron et al. (2020) study was also rated as “high risk” for selective outcome reporting bias. In this study, the authors collected outcome data at multiple time points but only reported outcome data for baseline and the 15‐week measurements.

Third, it is important to note that treatments for these studies might have different effects for those with a strong ideological leaning as both studies did not recruit participants from radical or extreme left‐ or right‐leaning websites. While both study samples represented more diversity than the traditional convenience sample of students, these samples might differ from users who possess extremist anti‐government or anti‐authority violent ideologies and Salafi‐jihadi ideologies in their inclination to post hateful comments. These online interventions might have different effects on radicalized people.

### 6.4 Potential biases in the review process

We did not identify any specific biases in the systematic review process. Although our review only identified two eligible online interventions, our search strategy was comprehensive, and we took all reasonable steps to locate eligible studies. In addition, and based on feedback from the Campbell Collaboration Crime and Justice Coordinating Group, we undertook an additional search of certain databases between March 17 and 24, 2022 to ensure that our search was not too restrictive (see Supporting Information: Appendix A for information regarding these additional searches). However, this additional search did not lead to any further studies being included in our final sample. Moreover, the use of several supplementary systematic search strategies reduced the likelihood that eligible evidence was not captured by the review. Specific strategies were employed to maintain consistency and validity for assessing the extent to which online interventions were effective in reducing online hate speech/cyberhate (RQ 1), such as independent double‐coding and weekly collaborative discussions about eligibility during the screening and coding process.

However, there are two limitations worth mentioning. First, findings in this review include studies published before January 1, 2021. As such, this review omits eligible studies conducted in 2021. Thus, it is vital to update this review within 3–5 years to capture any new research. Second, based on our research team's background and training, eligible studies were limited to English and German languages. It is possible that additional eligible studies were screened out because they were published outside of these language domains (e.g., Korean, Spanish, etc.). Future review teams should look to expand their language skillsets or, alternatively (but cautiously), may rely on translation programs such as Google Translate to be inclusive and not omit studies published in other languages. Web translation programs, however, are currently still unreliable and thus problematic for rigorous review standards. Finally, our review excluded qualitative studies, which is an artifact of our exclusive focus on effectiveness studies. This may be an important source of bias in that qualitative studies could provide important insight about context, and thus, potential moderating variables. These potential variables could have been important independent variables to examine the magnitude of effect sizes. We point to the Blaya (2019) and Carthy et al. (2018) reviews, which examined a large amount of qualitative research and provide additional context to this meta‐analysis.

### 6.5 Agreements and disagreements with other studies or reviews

Due to the limited and mixed nature of evaluation and review literature on the effectiveness of interventions aimed at reducing online hate speech and cyberhate, the findings of this review do not reaffirm or contradict any existing review. Two reviews offer additional insight into the current state of online hate speech and cyberhate interventions. First, Carthy et al. (2018) assessed the effectiveness of counter‐narratives in reducing the risk of violent radicalization. While the authors found little evidence that counter‐narratives are effective at targeting the intent to act violently, they concluded that these counter‐narrative interventions might affect some risk factors (e.g., realistic threat, in‐group favoritism, out‐group hostility) related to violent radicalization. Second, Blaya (2019) conducted a narrative review of cyberhate interventions. While the author did not conduct a meta‐analysis, her review identified three key counter‐speech intervention areas: law, technology, and education. No specific intervention toward aggressors was found, and most projects focused on prevention or on victims, through confidence and skills building such as learning to speak out, report, and potentially react in an appropriate way. We support the recommendations offered by Carthy et al. (2018) and Blaya (2019) that additional research is needed in this area to properly interpret the effectiveness of online interventions aimed at reducing online hate speech and cyberhate.

## 7 AUTHORS' CONCLUSIONS

### 7.1 Implications for practice

Two studies met our eligibility criteria by rigorously testing online interventions designed to reduce online hate speech/cyberhate, which highlights a gap in research and also has implications for practice. These types of studies and evaluations of interventions need to be included in near future research agendas to inform the work of practitioners and policymakers more effectively. The process of reducing online hate speech/cyberhate likely requires a great deal of theoretical complexity and methodological rigor to work effectively in preventing violent extremism (PVE) and countering violent extremism (CVE) domains. With the emergence of further, rigorous research, the extent of its ability to effectively reduce online hate speech/cyberhate will become clearer. While this project takes us one step closer toward better understanding the complex and multifaceted nature of hate speech and cyberhate messaging, we have insufficient evidence to determine the effectiveness of online hate speech/cyberhate interventions for reducing the creation and/or consumption of hateful content online.

### 7.2 Implications for research

Based on our review, we offer several recommendations for future research agendas. First, additional online interventions need to be conducted to clearly interpret the effectiveness of online hate speech and cyberhate interventions, focusing on random assignment of subjects into experimental and control groups. Researchers must experimentally control for the effect of the intervention if we are to test the ability of online interventions to reduce hateful types of expression on the grounds of race, skin tone/hue, national or ethnic origin, descent, age, disability, language, religion or belief, sex, gender, gender identity, sexual orientation and other personal characteristics or status. Along these lines, assessment of these aforementioned interventions should also be included in future research agendas.

Second, further experimental studies are needed to be able to examine the effect of online interventions on additional outcome measures of interest (see Windisch et al., 2021). For instance, research must attend to the importance of cyberhate and online hate speech within the context of creating (e.g., making videos, posting, sharing, liking, etc.) and transmitting hateful content rather than the classification and identification of online hate speech/cyberhate and its selected users. As mentioned, many interventions were excluded from this review as they measured outcomes related to the accuracy of computer algorithms to classify and identify hateful online content rather than overall effectiveness at reducing cyberhate and online hate speech. If online interventions are to become an evidence‐based tool for reducing online hate speech/cyberhate, more emphasis should be given to outcomes that measure the creation and transmission of hateful content.

There is also a need for more empirical studies of online interventions that focus their outcomes specifically on online behavior. In this review, we found some studies that tested online interventions but then investigated subjects' potential changes in offline behavior instead of also testing for any perceived changes in online behavior. There is also an opportunity here to test if such online interventions can make a difference in both the online and offline world, as it is likely that online interventions could influence both behaviors. However, further empirical testing is necessary in this regard. We encourage research into truly innovative approaches to addressing this problem that are radically distinct from existing programs. Third, echoing the recommendation put forth by Carthy et al. (2018), we encourage researchers to clearly specify the theoretical frameworks that guided their online intervention and/or campaign. While social norming (Elster, 1989; also see Bicchieri, 2005) emerged as a common theoretical perspective among the eligible studies, there are other useful theoretical frameworks (e.g., social identity theory, terror management theory, subjective uncertainty reduction theory) that warrant thoughtful consideration and testing.

Fourth, we suggest that future studies should focus on exploring online interventions for individuals who may have already been exposed to and/or have become radicalized by more extremist ideologies and/or who have moved on to more radical platforms with fewer rules around hateful content creation online. As pointed out at the beginning of this review and within our protocol (Windisch et al., 2021), hate speech and other prejudice‐motivated behaviors need to be considered on a continuum of victimization (Bowling, 1993), with more extreme forms of prejudice‐motivated violence founded on “lower level” acts of prejudice and bias (Allport, 1954). Hateful content online on such lower levels of the prejudice scale should therefore not be ignored, and the two studies we found within our systematic review explored interventions at such lower levels of prejudice. However, more empirical studies are necessary to explore online interventions that address online behavior of individuals who have already advanced to more extreme forms of prejudice‐motivated violence.

Finally, given the scarcity of experimental (random assignment) and quasi‐experimental evaluations of online hate speech/cyberhate interventions, researchers may consider conducting a qualitative evidence synthesis (Barnett‐Page & Thomas, 2009; Thorne et al., 2004). A qualitative evidence synthesis integrates the findings from multiple primary qualitative studies. Findings from qualitative evidence synthesis are generally more robust and useful than those from individual primary qualitative studies as they bring together evidence from multiple studies, thus providing richer data than offered by a single study. Most importantly, a qualitative evidence synthesis can identify patterns in the data and explore similarities and differences across settings. Such insights can be used alongside effectiveness evidence to inform all stages of developing an intervention, including identifying the relevant interventions and outcomes at the scoping stage, synthesizing, and evaluating evidence, formulating recommendations, and developing implementation considerations.

## CONTRIBUTIONS OF AUTHORS

### 1 Content

Steven Windisch and Elizabeth Jenaway have extensive background knowledge of terrorism, radicalization, violence, disengagement, and deradicalization. Susann Wiedlitzka has extensive background knowledge of hate crimes and hate speech, and has been involved in projects investigating online platforms and far‐right extremist responses to “in real life” attacks..

### 2 Systematic review methods

Ajima Olaghere has extensive expertise in statistical analyses. She has co‐authored two Campbell Systematic Reviews, one on youth curfews and the other on police‐initiated diversion of low‐risk youth.

### 3 Statistical analysis

Ajima Olaghere and Susann Wiedlitzka have extensive expertise in statistical analyses. Elizabeth Jenaway provided substantial assistance with data management and cleaning.

### 4 Information retrieval

Steven Windisch, Ajima Olaghere, Susann Wiedlitzka, and Elizabeth Jenaway all have experience performing systematic searches on various topics and retrieving studies and documents for review.

## DECLARATION OF INTERESTS

Ajima Olaghere is an editor for the Crime and Justice Coordinating Group within the Campbell Collaboration. She has recused herself in the review of the protocol and completed systematic review. Susann Wiedlitzka and the editor overseeing this review know each other on a personal and professional level. Susann Wiedlitzka has also started work on a new hate crime project with another Crime and Justice editor. The following steps have been taken to deal with this potential conflict of interest: Multiple layers of the review (CJCG Co‐chair review, EiC review, Campbell Methods Group review) are already in place due to this being a fast‐tracked review. In addition, David B. Wilson (Methods Editor) has reviewed and co‐signed action letters and associated materials and has been copied into any communications between the editors and the authors of this review. The editor with a current professional relationship with Susann Wiedlitzka recused herself from the editorial processes for the completed review, but oversaw the editorial process for the protocol, before her current working relationship with Susann Wiedlitzka.

## SOURCES OF SUPPORT

This review was supported by the Horizon 2020 (Grant No.: 699824), DHS Science and Technology Directorate, and the Five Research and Development (5RD) Countering Violent Extremism Network.

Internal support: We also received support from the University of Auckland School of Social Sciences Performance Based Research Fund (PBRF, Round 4, 2020 and 2021).

## Supporting information

Supporting information.
