# Supplementary material for: Online interventions for reducing hate speech and cyberhate: A systematic review
Source: Campbell Syst Rev. 2022 May 25;18(2):e1243. doi: 10.1002/cl2.1243 (PMC9133687; doi:10.1002/cl2.1243)
Supplement: Supplementary file 1 — Supporting information. [file CL2-18-e1243-s001.docx]

**Appendix A. Systematic Search Notes**

Note. Please note that the actual yield found across each source is the number prior to removing duplicates. The following search notes include a default search field for all databases, websites, and journals. Any notes including specific fields indicate the use of specific fields as filters. We provide search notes for our search from August to December 2020 below.

| **August to December 2020** | |
| --- | --- |
| **Field** | **Description** |
| Search date | 9-10 November 2020; 20 November 2020; 14 December 2020 |
| Initials | AMO, SW, SMW |
| Database/Website/Journal/Strategy | Hand searching |
| Final Search String | n/a |
| Reported Yield | 572 |
| Actual Yield | 113 |
| Notes | AMO added 60 references from two sources:   1. Blaya, C. (2019). Cyberhate: A review and content analysis of intervention strategies. Aggression and Violent Behavior, 45, 163-172. 2. Bliuc, A., Faulkner, N., Jakubowicz, A., & McGarty, C. (2018). Online networks of racial hate: A systematic review of 10 years of research on cyber-racism. Computers in Human Behavior, 87, 75-86.   SW added 41 references from two sources:   1. Strachan, A. L. (2014). Interventions to counter hate speech. GSDRC Applied Research Services, 1-8. (15 sources added) only imported references from section 5 on Countering Extremism Online 2. Winter, C., Neumann, P., Meleagrou-Hitchens, A., Ranstorp, M., Vidino, L., & Fürst, J. (2020). Online extremism: Research trends in internet activism, radicalization, and counter-strategies. International Journal of Conflict and Violence, 14(2), 1-20. doi:10.4119/ijcv-3809 (26 sources added)   SMW added 12 references from two sources:   1. Brown, I., & Cowls, J. (2015). Check the web: assessing the ethics and politics of policing the Internet for extremist material. Voxpol: Report http://dx. doi. org/http://voxpol. eu/category/publications/vox-pol-publications. 2. Hassan, G., Brouillette-Alarie, S., Alava, S., Frau-Meigs, D., Lavoie, L., Fetiu, A., ... & Sieckelinck, S. (2018). Exposure to extremist online content could lead to violent radicalization: A systematic review of empirical evidence. International journal of developmental science, 12(1-2), 71-88. |
| Search date | 28 December 2020 |
| Initials | AMO |
| Database/Website/Journal/Strategy | EBSCOHost Research Databases (<https://www.ebsco.com/products/research-databases>) |
| Final Search String | AB (online OR “social media” OR internet OR Twitter OR Facebook OR 8Chan OR 8Kun OR Gab OR Telegram OR TikTok OR Reddit OR WhatsApp OR Instagram OR “social networking site*” OR cybervictimization OR “online incivility”) AND (“hate speech” OR cyberhate OR extrem* narrative* OR racis* OR radical* OR speech OR ideolog* OR islamophobi* OR homophobi* OR transphobi* OR misogyny OR disablism OR discrim* OR terror*) AND (interven* OR option* OR strategy* OR “counter narrative*” OR “nudge” OR “norm* intervention” OR “norm* nudge” OR counternarrative* OR “alternative narrative*” OR campaign* OR counter* OR peer-to-peer OR prevent* OR disrupt* OR stop* OR fight* OR redirect* OR “censoring hate content”) AND (comparison* OR quantitative OR quasi- experiment*) |
| Reported Yield | 682 |
| Actual Yield | 501 (search engine removed: “Exact duplicates removed from the results.”) |
| Notes | Restrictions:   - January 1990 – December 2020 - English, German, Arabic, Persian - Searched within abstracts   EBSCOHost is a database aggregator. Through this aggregator we were able to simultaneously search the following databases:   - Academic Search Complete - Academic Search Ultimate (do not have via TU) - Communication and Mass Media Complete - Communication Abstracts - Criminal Justice Abstracts with Full Text - ERIC - Military & Government Collection - PsycARTICLES - Psychology and Behavioral Sciences Collection - PsycINFO   Search iterations:   1. without language restriction, default search bar (online OR “social media” OR internet OR Twitter OR Facebook OR 8Chan OR 8Kun OR Gab OR Telegram OR TikTok OR Reddit OR WhatsApp OR Instagram OR “social networking site*” OR cybervictimization OR “online incivility”) AND (“hate speech” OR cyberhate OR extrem* narrative* OR racis* OR radical* OR speech OR ideolog* OR islamophobi* OR homophobi* OR transphobi* OR misogyny OR disablism OR discrim* OR terror*) AND (interven* OR option* OR strategy* OR “counter narrative*” OR “nudge” OR “norm* intervention” OR “norm* nudge” OR counternarrative* OR “alternative narrative*” OR campaign* OR counter* OR peer-to-peer OR prevent* OR disrupt* OR stop* OR fight* OR redirect* OR “censoring hate content”) AND (comparison* OR quantitative OR quasi-experiment* OR survey* OR interview* OR poll* OR mixed-methods OR individual-level OR group-level OR control* OR experiment* OR study OR studies OR evaluat* OR MTurk OR longitudinal OR random* OR “digital method*” OR “machine learning” OR “natural language processing” OR multisectoral OR review*); yield: 12,591 2. #1 + removed “expanders”; yield: 11,771 3. with language restriction (Arabic, English, German, Persian): (online OR “social media” OR internet OR Twitter OR Facebook OR 8Chan OR 8Kun OR Gab OR Telegram OR TikTok OR Reddit OR WhatsApp OR Instagram OR “social networking site*” OR cybervictimization OR “online incivility”) AND (“hate speech” OR cyberhate OR extrem* narrative* OR racis* OR radical* OR speech OR ideolog* OR islamophobi* OR homophobi* OR transphobi* OR misogyny OR disablism OR discrim* OR terror*) AND (interven* OR option* OR strategy* OR “counter narrative*” OR “nudge” OR “norm* intervention” OR “norm* nudge” OR counternarrative* OR “alternative narrative*” OR campaign* OR counter* OR peer-to-peer OR prevent* OR disrupt* OR stop* OR fight* OR redirect* OR “censoring hate content”) AND (comparison* OR quantitative OR quasi-experiment* OR survey* OR interview* OR poll* OR mixed-methods OR individual-level OR group-level OR control* OR experiment* OR study OR studies OR evaluat* OR MTurk OR longitudinal OR random* OR “digital method*” OR “machine learning” OR “natural language processing” OR multisectoral OR review*); yield: 11,433 4. with language restriction + search in abstract (Arabic, English, German, Persian): AB((online OR “social media” OR internet OR Twitter OR Facebook OR 8Chan OR 8Kun OR Gab OR Telegram OR TikTok OR Reddit OR WhatsApp OR Instagram OR “social networking site*” OR cybervictimization OR “online incivility”) AND (“hate speech” OR cyberhate OR extrem* narrative* OR racis* OR radical* OR speech OR ideolog* OR islamophobi* OR homophobi* OR transphobi* OR misogyny OR disablism OR discrim* OR terror*) AND (interven* OR option* OR strategy* OR “counter narrative*” OR “nudge” OR “norm* intervention” OR “norm* nudge” OR counternarrative* OR “alternative narrative*” OR campaign* OR counter* OR peer-to-peer OR prevent* OR disrupt* OR stop* OR fight* OR redirect* OR “censoring hate content”) AND (comparison* OR quantitative OR quasi-experiment* OR survey* OR interview* OR poll* OR mixed-methods OR individual-level OR group-level OR control* OR experiment* OR study OR studies OR evaluat* OR MTurk OR longitudinal OR random* OR “digital method*” OR “machine learning” OR “natural language processing” OR multisectoral OR review*)); yield: 3,932 5. AB (online OR “social media” OR internet OR Twitter OR Facebook OR 8Chan OR 8Kun OR Gab OR Telegram OR TikTok OR Reddit OR WhatsApp OR Instagram OR “social networking site*” OR cybervictimization OR “online incivility”) AND (“hate speech” OR cyberhate OR extrem* narrative* OR racis* OR radical* OR speech OR ideolog* OR islamophobi* OR homophobi* OR transphobi* OR misogyny OR disablism OR discrim* OR terror*) AND (interven* OR option* OR strategy* OR “counter narrative*” OR “nudge” OR “norm* intervention” OR “norm* nudge” OR counternarrative* OR “alternative narrative*” OR campaign* OR counter* OR peer-to-peer OR prevent* OR disrupt* OR stop* OR fight* OR redirect* OR “censoring hate content”) AND (comparison* OR quantitative OR quasi- experiment*); yield: 682 |
| Search date | 21 December 2020 |
| Initials | AMO |
| Database/Website/Journal/Strategy | ProQuest (<https://www.proquest.com/>) |
| Final Search String | AB((online OR “social media” OR internet OR “social networking site*” OR cybervictimization OR “online incivility”) AND (“hate speech” OR cyberhate OR extrem* narrative* OR racis* OR radical* OR speech OR ideolog* OR islamophobi* OR homophobi* OR transphobi* OR misogyny OR disablism OR discrim* OR terror*) AND (interven* OR option* OR strategy* OR “counter narrative*” OR “nudge” OR “norm* intervention” OR “norm* nudge” OR counternarrative* OR “alternative narrative*” OR campaign* OR counter* OR peer-to-peer OR prevent* OR disrupt* OR stop* OR fight* OR redirect* OR “censoring hate content”) AND (quasi-experiment* OR experiment*)) |
| Reported Yield | 228 |
| Actual Yield | 227 |
| Notes | Restrictions:   - After this date: 1/1/1990   ProQuest Central is a database aggregator. Through this aggregator we were able to simultaneously search the following databases:   - Applied Social Sciences Index & Abstracts (ASSIA) - Criminal Justice Database - Education Resources Information Center (ERIC) - International Bibliography of the Social Sciences (IBSS) - Gender Watch - National Criminal Justice Reference Service (NCJRS) - Public Affairs Information Service (PAIS) - Policy File Index - ProQuest Criminal Justice - ProQuest Dissertation & Theses Global - ProQuest Political Science Database - ProQuest Social Science Database - ProQuest Sociological Abstracts - ProQuest Sociology (Sociology database) - Worldwide Political Science Abstracts   Search iterations:   1. (online OR “social media” OR internet OR Twitter OR Facebook OR 8Chan OR 8Kun OR Gab OR Telegram OR TikTok OR Reddit OR WhatsApp OR Instagram OR “social networking site*” OR cybervictimization OR “online incivility”) AND (“hate speech” OR cyberhate OR extrem* narrative* OR racis* OR radical* OR speech OR ideolog* OR islamophobi* OR homophobi* OR transphobi* OR misogyny OR disablism OR discrim* OR terror*) AND (interven* OR option* OR strategy* OR “counter narrative*” OR “nudge” OR “norm* intervention” OR “norm* nudge” OR counternarrative* OR “alternative narrative*” OR campaign* OR counter* OR peer-to-peer OR prevent* OR disrupt* OR stop* OR fight* OR redirect* OR “censoring hate content”) AND (comparison* OR quantitative OR quasi-experiment* OR survey* OR interview* OR poll* OR mixed-methods OR individual-level OR group-level OR control* OR experiment* OR study OR studies OR evaluat* OR MTurk OR longitudinal OR random* OR “digital method*” OR “machine learning” OR “natural language processing” OR multisectoral OR review*); yield: 834,498 2. AB((online OR “social media” OR internet OR Twitter OR Facebook OR 8Chan OR 8Kun OR Gab OR Telegram OR TikTok OR Reddit OR WhatsApp OR Instagram OR “social networking site*” OR cybervictimization OR “online incivility”) AND (“hate speech” OR cyberhate OR extrem* narrative* OR racis* OR radical* OR speech OR ideolog* OR islamophobi* OR homophobi* OR transphobi* OR misogyny OR disablism OR discrim* OR terror*) AND (interven* OR option* OR strategy* OR “counter narrative*” OR “nudge” OR “norm* intervention” OR “norm* nudge” OR counternarrative* OR “alternative narrative*” OR campaign* OR counter* OR peer-to-peer OR prevent* OR disrupt* OR stop* OR fight* OR redirect* OR “censoring hate content”) AND (comparison* OR quantitative OR quasi-experiment* OR survey* OR interview* OR poll* OR mixed-methods OR individual-level OR group-level OR control* OR experiment* OR study OR studies OR evaluat* OR MTurk OR longitudinal OR random* OR “digital method*” OR “machine learning” OR “natural language processing” OR multisectoral OR review*)); yield: 2,714 3. AB((online OR “social media” OR internet OR “social networking site*” OR cybervictimization OR “online incivility”) AND (“hate speech” OR cyberhate OR extrem* narrative* OR racis* OR radical* OR speech OR ideolog* OR islamophobi* OR homophobi* OR transphobi* OR misogyny OR disablism OR discrim* OR terror*) AND (interven* OR option* OR strategy* OR “counter narrative*” OR “nudge” OR “norm* intervention” OR “norm* nudge” OR counternarrative* OR “alternative narrative*” OR campaign* OR counter* OR peer-to-peer OR prevent* OR disrupt* OR stop* OR fight* OR redirect* OR “censoring hate content”) AND (quantitative OR quasi- experiment* OR experiment* OR study)); yield: 1,803 4. AB((online OR “social media” OR internet OR “social networking site*” OR cybervictimization OR “online incivility”) AND (“hate speech” OR cyberhate OR extrem* narrative* OR racis* OR radical* OR islamophobi* OR homophobi* OR transphobi* OR misogyny OR disablism OR terror*) AND (interven* OR “counter narrative*” OR “nudge” OR “norm* intervention” OR “norm* nudge” OR counternarrative* OR “alternative narrative*” OR campaign* OR counter* OR peer-to- peer OR prevent* OR redirect* OR “censoring hate content”) AND (quasi-experiment* OR experiment*)); yield: 44 5. AB((online OR “social media” OR internet OR “social networking site*” OR cybervictimization OR “online incivility”) AND (“hate speech” OR cyberhate OR extrem* narrative* OR racis* OR radical* OR speech OR ideolog* OR islamophobi* OR homophobi* OR transphobi* OR misogyny OR disablism OR discrim* OR terror*) AND (interven* OR option* OR strategy* OR “counter narrative*” OR “nudge” OR “norm* intervention” OR “norm* nudge” OR counternarrative* OR “alternative narrative*” OR campaign* OR counter* OR peer-to-peer OR prevent* OR disrupt* OR stop* OR fight* OR redirect* OR “censoring hate content”) AND (quasi-experiment* OR experiment*)); yield: 228 |
| Search date | 3 December 2020 |
| Initials | HN |
| Database/Website/Journal/Strategy | Academic One File (<https://www.gale.com/c/academic-onefile>) |
| Final Search String | Search limited to Keywords: (online OR “social media” OR internet OR Twitter OR Facebook OR 8Chan OR 8Kun OR Gab OR Telegram OR TikTok OR Reddit OR WhatsApp OR Instagram OR “social networking site*” OR cybervictimization OR “online incivility”) AND (“hate speech” OR cyberhate OR extrem* narrative* OR racis* OR radical* OR speech OR ideolog* OR islamophobi* OR homophobi* OR transphobi* OR misogyny OR disablism OR discrim* OR terror*) AND (interven* OR option* OR strategy* OR “counter narrative*” OR nudge OR norm* intervention OR “norm* nudge” OR counternarrative* OR “alternative narrative*” OR campaign* OR counter* OR peer-to-peer OR prevent* OR disrupt* OR stop* OR fight* OR redirect* OR “censoring hate content”) |
| Reported Yield | 1,118 |
| Actual Yield | 223 |
| Notes | The documents were sorted by relevance and the first 200 were imported manually. The remaining 918 documents were reviewed and those which seemed relevant imported. The criterion for identifying a relevant reference was finding one of the keywords in the title or the abstract.  Search iterations:   1. (online OR “social media” OR internet OR Twitter OR Facebook OR 8Chan OR 8Kun OR Gab OR Telegram OR TikTok OR Reddit OR WhatsApp OR Instagram OR “social networking site*” OR cybervictimization OR “online incivility”) AND (“hate speech” OR cyberhate OR extrem* narrative* OR racis* OR radical* OR speech OR ideolog* OR islamophobi* OR homophobi* OR transphobi* OR misogyny OR disablism OR discrim* OR terror*) AND (interven* OR option* OR strategy* OR “counter narrative*” OR nudge OR norm* intervention OR “norm* nudge” OR counternarrative* OR “alternative narrative*” OR campaign* OR counter* OR peer-to-peer OR prevent* OR disrupt* OR stop* OR fight* OR redirect* OR “censoring hate content”) AND (comparison* OR quantitative OR quasi-experiment* OR survey* OR interview* OR poll* OR mixed-methods OR individual-level OR group-level OR control* OR experiment* OR study OR studies OR evaluat* OR MTurk OR longitudinal OR random* OR “digital method*” OR “machine learning” OR “natural language processing” OR multisectoral OR review*) Results: 206,542 2. Search limited to Abstract: (online OR “social media” OR internet OR Twitter OR Facebook OR 8Chan OR 8Kun OR Gab OR Telegram OR TikTok OR Reddit OR WhatsApp OR Instagram OR “social networking site*” OR cybervictimization OR “online incivility”) AND (“hate speech” OR cyberhate OR extrem* narrative* OR racis* OR radical* OR speech OR ideolog* OR islamophobi* OR homophobi* OR transphobi* OR misogyny OR disablism OR discrim* OR terror*) AND (interven* OR option* OR strategy* OR “counter narrative*” OR nudge OR norm* intervention OR “norm* nudge” OR counternarrative* OR “alternative narrative*” OR campaign* OR counter* OR peer-to-peer OR prevent* OR disrupt* OR stop* OR fight* OR redirect* OR “censoring hate content”) Results: 356 3. Search limited to Keywords: (online OR “social media” OR internet OR Twitter OR Facebook OR 8Chan OR 8Kun OR Gab OR Telegram OR TikTok OR Reddit OR WhatsApp OR Instagram OR “social networking site*” OR cybervictimization OR “online incivility”) AND (“hate speech” OR cyberhate OR extrem* narrative* OR racis* OR radical* OR speech OR ideolog* OR islamophobi* OR homophobi* OR transphobi* OR misogyny OR disablism OR discrim* OR terror*) AND (interven* OR option* OR strategy* OR “counter narrative*” OR nudge OR norm* intervention OR “norm* nudge” OR counternarrative* OR “alternative narrative*” OR campaign* OR counter* OR peer-to-peer OR prevent* OR disrupt* OR stop* OR fight* OR redirect* OR “censoring hate content”) Results: 1118 4. Search limited to Keywords: (online OR “social media” OR internet OR Twitter OR Facebook OR 8Chan OR 8Kun OR Gab OR Telegram OR TikTok OR Reddit OR WhatsApp OR Instagram OR “social networking site*” OR cybervictimization OR “online incivility”) AND (“hate speech” OR cyberhate OR extrem* narrative* OR racis* OR radical* OR speech OR ideolog* OR islamophobi* OR homophobi* OR transphobi* OR misogyny OR disablism OR discrim* OR terror*) AND (interven* OR option* OR strategy* OR “counter narrative*” OR nudge OR norm* intervention OR “norm* nudge” OR counternarrative* OR “alternative narrative*” OR campaign* OR counter* OR peer-to-peer OR prevent* OR disrupt* OR stop* OR fight* OR redirect* OR “censoring hate content”) AND (comparison* OR quantitative OR quasi-experiment* OR survey* OR interview* OR poll* OR mixed-methods OR individual-level OR group-level OR control* OR experiment* OR study OR studies OR evaluat* OR MTurk OR longitudinal OR random* OR “digital method*” OR “machine learning” OR “natural language processing” OR multisectoral OR review*) Results: 715 |
| Search date | 3 December 2021 |
| Initials | HN |
| Database/Website/Journal/Strategy | Australian Federal Police Digest (AFPD) (<https://search.informit.org/ourcollections/indexes/afpd>) |
| Final Search String | Search limited to Abstract: (online OR “social media” OR internet OR Twitter OR Facebook OR 8Chan OR 8Kun OR Gab OR Telegram OR TikTok OR Reddit OR WhatsApp OR Instagram OR “social networking site*” OR cybervictimization OR “online incivility”) AND (“hate speech” OR cyberhate OR extrem* narrative* OR racis* OR radical* OR speech OR ideolog* OR islamophobi* OR homophobi* OR transphobi* OR misogyny OR disablism OR discrim* OR terror*) AND (interven* OR option* OR strategy* OR “counter narrative*” OR nudge OR norm* intervention OR “norm* nudge” OR counternarrative* OR “alternative narrative*” OR campaign* OR counter* OR peer-to-peer OR prevent* OR disrupt* OR stop* OR fight* OR redirect* OR “censoring hate content”) AND (comparison* OR quantitative OR quasi-experiment* OR survey* OR interview* OR poll* OR mixed-methods OR individual-level OR group-level OR control* OR experiment* OR study OR studies OR evaluat* OR MTurk OR longitudinal OR random* OR “digital method*” OR “machine learning” OR “natural language processing” OR multisectoral OR review*)  Retrieved the first 500, sorted by relevance |
| Reported Yield | 52,311 |
| Actual Yield | 497 |
| Notes | Search iterations:   1. Search in All Fields: (online OR “social media” OR internet OR Twitter OR Facebook OR 8Chan OR 8Kun OR Gab OR Telegram OR TikTok OR Reddit OR WhatsApp OR Instagram OR “social networking site*” OR cybervictimization OR “online incivility”) AND (“hate speech” OR cyberhate OR extrem* narrative* OR racis* OR radical* OR speech OR ideolog* OR islamophobi* OR homophobi* OR transphobi* OR misogyny OR disablism OR discrim* OR terror*) AND (interven* OR option* OR strategy* OR “counter narrative*” OR nudge OR norm* intervention OR “norm* nudge” OR counternarrative* OR “alternative narrative*” OR campaign* OR counter* OR peer-to- peer OR prevent* OR disrupt* OR stop* OR fight* OR redirect* OR “censoring hate content”) AND (comparison* OR quantitative OR quasi-experiment* OR survey* OR interview* OR poll* OR mixed-methods OR individual-level OR group-level OR control* OR experiment* OR study OR studies OR evaluat* OR MTurk OR longitudinal OR random* OR “digital method*” OR “machine learning” OR “natural language processing” OR multisectoral OR review*) Results: 187,427 2. Search limited to Abstract: (online OR “social media” OR internet OR Twitter OR Facebook OR 8Chan OR 8Kun OR Gab OR Telegram OR TikTok OR Reddit OR WhatsApp OR Instagram OR “social networking site*'“ OR cybervictimization OR “online incivility”) AND (“hate speech” OR cyberhate OR extrem* narrative* OR racis* OR radical* OR speech OR ideolog* OR islamophobi* OR homophobi* OR transphobi* OR misogyny OR disablism OR discrim* OR terror*) AND (interven* OR option* OR strategy* OR “counter narrative*” OR nudge OR norm* intervention OR “norm* nudge” OR counternarrative* OR “alternative narrative*” OR campaign* OR counter* OR peer-to-peer OR prevent* OR disrupt* OR stop* OR fight* OR redirect* OR “censoring hate content”) AND (comparison* OR quantitative OR quasi-experiment* OR survey* OR interview* OR poll* OR mixed-methods OR individual-level OR group-level OR control* OR experiment* OR study OR studies OR evaluat* OR MTurk OR longitudinal OR random* OR “digital method*” OR “machine learning” OR “natural language processing” OR multisectoral OR review*) Results: 52,311 |
| Search date | 7 December 2020 |
| Initials | HN |
| Database/Website/Journal/Strategy | ArticleFirst (FirstSearch) (<https://firstsearch-oclc-org.libproxy.temple.edu/WebZ/FSPrefs?entityjsdetect=:javascript=true:screensize=large:sessionid=fsap06pxm1-1680-ky50e160-8jdful:entitypagenum=1:0>) |
| Final Search String | Search limited to Keyword: (online OR “social media” OR internet OR Twitter OR Facebook OR 8Chan OR 8Kun OR Gab OR Telegram OR TikTok OR Reddit OR WhatsApp OR Instagram OR “social networking site*” OR cybervictimization OR “online incivility”) AND (“hate speech” OR cyberhate OR extrem* narrative* OR racis* OR radical* OR speech OR ideolog* OR islamophobi* OR homophobi* OR transphobi* OR misogyny OR disablism OR discrim* OR terror*) |
| Reported Yield | 2,253 |
| Actual Yield | 560 |
| Notes | Retrieved approximately the first 500, sorted by relevance  Search iterations:   1. Search limited to Keyword: (online OR “social media” OR internet OR Twitter OR Facebook OR 8Chan OR 8Kun OR Gab OR Telegram OR TikTok OR Reddit OR WhatsApp OR Instagram OR “social networking site*” OR cybervictimization OR “online incivility”) AND (“hate speech” OR cyberhate OR extrem* narrative* OR racis* OR radical* OR speech OR ideolog* OR islamophobi* OR homophobi* OR transphobi* OR misogyny OR disablism OR discrim* OR terror*) AND (interven* OR option* OR strategy* OR “counter narrative*” OR nudge OR norm* intervention OR “norm* nudge” OR counternarrative* OR “alternative narrative*” OR campaign* OR counter* OR peer-to-peer OR prevent* OR disrupt* OR stop* OR fight* OR redirect* OR “censoring hate content”)   *Error: Your search contained a term with wildcard characters that matched too many terms. Please try to add more letters to make your term more specific.*   1. Search limited to Keyword: (online OR “social media” OR internet OR Twitter OR Facebook OR 8Chan OR 8Kun OR Gab OR Telegram OR TikTok OR Reddit OR WhatsApp OR Instagram OR “social networking site*” OR cybervictimization OR “online incivility”) AND (“hate speech” OR cyberhate OR extrem* narrative* OR racis* OR radical* OR speech OR ideolog* OR islamophobi* OR homophobi* OR transphobi* OR misogyny OR disablism OR discrim* OR terror*) AND (interven* OR nudge OR norm* OR campaign* OR counter* OR prevent*)   *Error: Your search contained a term with wildcard characters that matched too many terms. Please try to add more letters to make your term more specific*.   1. Search limited to Keyword: (online OR “social media” OR internet OR Twitter OR Facebook OR 8Chan OR 8Kun OR Gab OR Telegram OR TikTok OR Reddit OR WhatsApp OR Instagram OR “social networking site*” OR cybervictimization OR “online incivility”) AND (“hate speech” OR cyberhate OR extrem* narrative* OR racis* OR radical* OR speech OR ideolog* OR islamophobi* OR homophobi* OR transphobi* OR misogyny OR disablism OR discrim* OR terror*) AND (interven* OR counter* OR prevent*) Results: 22 2. Search limited to Keyword: (online OR “social media” OR internet OR Twitter OR Facebook OR 8Chan OR 8Kun OR Gab OR Telegram OR TikTok OR Reddit OR WhatsApp OR Instagram OR “social networking site*” OR cybervictimization OR “online incivility”) AND (“hate speech” OR cyberhate OR extrem* narrative* OR racis* OR radical* OR speech OR ideolog* OR islamophobi* OR homophobi* OR transphobi* OR misogyny OR disablism OR discrim* OR terror*) Results: 2,253 |
| Search date | 25 - 26 November 2020 |
| Initials | EL |
| Database/Website/Journal/Strategy | Cambridge Journals Online (<https://www.cambridge.org/core/>) |
| Final Search String | (online radical* OR online terror* OR online extrem* OR “hate speech” OR cyberhate OR “social media” OR internet OR Twitter OR Facebook OR 8Chan OR 8Kun OR Gab OR Telegram OR TikTok OR Reddit OR WhatsApp OR Instagram OR “social networking site*” OR extrem* narrative* OR racis* OR radical* OR dangerous speech OR ideology OR islamophobia OR homophobia OR transphobia OR misogyny OR disablism AND interven* OR option* OR strategy* OR “counter narrative*” OR counternarrative* OR “alternative narrative*” OR campaign* OR counter* OR peer-to-peer OR prevent* OR disrupt* OR stop* OR fight* OR redirect*) |
| Reported Yield | 207,604 |
| Actual Yield | 1,065 (Retrieved first 1000 sorted by relevance; first 50 pages) |
| Notes | Each page was uploaded via Zotero connector, page was refreshed and the list was reloaded when the following error occurred “An error occurred while saving this item. See Troubleshooting Translator Issues for more information.” Thus there may be repeats. Despite doing up to 5 attempts, the error kept occurring. The default search field was used as there is no option to refine the search field to all search fields, keywords, abstract, title, author, etc. and the database did not indicate the search fields they use for the default search.  Search iterations:   1. date range: published after 1989 and published before 2021 sorted by relevance: (online radical* OR online terror* OR online extrem* OR “hate speech” OR cyberhate OR “social media” OR internet OR Twitter OR Facebook OR 8Chan OR 8Kun OR Gab OR Telegram OR TikTok OR Reddit OR WhatsApp OR Instagram OR “social networking site*” OR extrem* narrative* OR racis* OR radical* OR dangerous speech OR ideology OR islamophobia OR homophobia OR transphobia OR misogyny OR disablism)   Results: 140,352  Results refined with 'only show content I have access to': 106,865   1. (narrative*” OR campaign* OR counter* OR peer-to-peer OR prevent* OR disrupt* OR stop* OR fight* OR redirect*)   Results: 206,865  Results refined with 'only show content I have access to': 158,059   1. date range: published after 1989 and published before 2021 sorted by relevance )online radical* OR online terror* OR online extrem* OR “hate speech” OR cyberhate OR “social media” OR internet OR Twitter OR Facebook OR 8Chan OR 8Kun OR Gab OR Telegram OR TikTok OR Reddit OR WhatsApp OR Instagram OR “social networking site*” OR extrem* narrative* OR racis* OR radical* OR dangerous speech OR ideology OR islamophobia OR homophobia OR transphobia OR misogyny OR disablism AND interven* OR option* OR strategy* OR “counter narrative*” OR counternarrative* OR “alternative narrative*” OR campaign* OR counter* OR peer-to-peer OR prevent* OR disrupt* OR stop* OR fight* OR redirect* AND comparison* OR quantitative OR qualitative OR quasi-experiment* OR survey* OR interview* OR poll* OR mixed-methods OR individual-level OR group-level OR control* OR experiment* OR study OR studies OR evaluat* OR MTurk OR longitudinal OR random* OR “digital method*” OR “machine learning” OR “natural language processing” OR multisectoral OR review*)   Results: 589,936  Results refined with 'only show content I have access to': 436,787   1. (narrative*” OR campaign* OR counter* OR peer-to-peer OR prevent* OR disrupt* OR stop* OR fight* OR redirect* AND comparison* OR quantitative OR qualitative OR quasi-experiment* OR survey* OR interview* OR poll* OR mixed-methods OR individual-level OR group-level OR control* OR experiment* OR study OR studies OR evaluat* OR MTurk OR longitudinal OR random* OR “digital method*” OR “machine learning” OR “natural language processing” OR multisectoral OR review* AND improvements in affect* OR reduced content OR reduced creation OR reduced consumption OR reduce visiting hate sites OR reduced posting messages OR blocked accounts OR flagged accounts OR reporting OR flagging OR reduce anger OR emotional states OR reduce fear OR reduce emotional unrest OR reduce depression OR reduce anxiety OR reduce mood swings OR perpetration OR victimization OR digital footprint OR improvements OR adverse effects OR economic issues)   Results: 734,965  Results refined with 'only show content I have access to': 555,645   1. (online radical* OR online terror* OR online extrem* OR “hate speech” OR cyberhate OR “social media” OR internet OR Twitter OR Facebook OR 8Chan OR 8Kun OR Gab OR Telegram OR TikTok OR Reddit OR WhatsApp OR Instagram OR “social networking site*” OR extrem* narrative* OR racis* OR radical* OR dangerous speech OR ideology OR islamophobia OR homophobia OR transphobia OR misogyny OR disablism AND interven* OR option* OR strategy* OR “counter narrative*” OR counternarrative* OR “alternative narrative*” OR campaign* OR counter* OR peer-to-peer OR prevent* OR disrupt* OR stop* OR fight* OR redirect)   Results: 207,604  Results refined with 'only show content I have access to': 145,579 |
| Search date | 16 August 2020; 24 November 2020 |
| Initials | SW |
| Database/Website/Journal/Strategy | CINCH: Australian Criminology Database (via Informit) (<https://www.aic.gov.au/cinch>) |
| Final Search String | Search limited to Keyword: (online OR “social media” OR internet OR Twitter OR Facebook OR 8Chan OR 8Kun OR Gab OR Telegram OR TikTok OR Reddit OR WhatsApp OR Instagram OR “social networking site*” OR cybervictimization OR “online incivility”) AND (“hate speech” OR cyberhate OR extrem* narrative* OR racis* OR radical* OR speech OR ideolog* OR islamophobi* OR homophobi* OR transphobi* OR misogyny OR disablism OR discrim* OR terror*) |
| Reported Yield | 442 |
| Actual Yield | 442 |
| Notes | Year: 1990-2020  Search iterations:   1. Search limited to Keyword: (online OR “social media” OR internet OR Twitter OR Facebook OR 8Chan OR 8Kun OR Gab OR Telegram OR TikTok OR Reddit OR WhatsApp OR Instagram OR “social networking site*” OR cybervictimization OR “online incivility”) AND (“hate speech” OR cyberhate OR extrem* narrative* OR racis* OR radical* OR speech OR ideolog* OR islamophobi* OR homophobi* OR transphobi* OR misogyny OR disablism OR discrim* OR terror*) AND (interven* OR option* OR strategy* OR “counter narrative*” OR “nudge” OR “norm* intervention” OR “norm* nudge” OR counternarrative* OR “alternative narrative*” OR campaign* OR counter* OR peer-to-peer OR prevent* OR disrupt* OR stop* OR fight* OR redirect* OR “censoring hate content”) AND (comparison* OR quantitative OR quasi-experiment* OR survey* OR interview* OR poll* OR mixed-methods OR individual-level OR group-level OR control* OR experiment* OR study OR studies OR evaluat* OR MTurk OR longitudinal OR random* OR “digital method*” OR “machine learning” OR “natural language processing” OR multisectoral OR review*) - 113 2. Search limited to Keyword: (online OR “social media” OR internet OR Twitter OR Facebook OR 8Chan OR 8Kun OR Gab OR Telegram OR TikTok OR Reddit OR WhatsApp OR Instagram OR “social networking site*” OR cybervictimization OR “online incivility”) AND (“hate speech” OR cyberhate OR extrem* narrative* OR racis* OR radical* OR speech OR ideolog* OR islamophobi* OR homophobi* OR transphobi* OR misogyny OR disablism OR discrim* OR terror*) AND (interven* OR option* OR strategy* OR “counter narrative*” OR “nudge” OR “norm* intervention” OR “norm* nudge” OR counternarrative* OR “alternative narrative*” OR campaign* OR counter* OR peer-to-peer OR prevent* OR disrupt* OR stop* OR fight* OR redirect* OR “censoring hate content”) – 235 3. Search limited to Keyword: (online OR “social media” OR internet OR Twitter OR Facebook OR 8Chan OR 8Kun OR Gab OR Telegram OR TikTok OR Reddit OR WhatsApp OR Instagram OR “social networking site*” OR cybervictimization OR “online incivility”) AND (“hate speech” OR cyberhate OR extrem* narrative* OR racis* OR radical* OR speech OR ideolog* OR islamophobi* OR homophobi* OR transphobi* OR misogyny OR disablism OR discrim* OR terror*) – 442 |
| Search date | 16 November 2020; 21 December 2020 |
| Initials | HN, AMO |
| Database/Website/Journal/Strategy | Columbia International Affairs Online (CIAO) (<https://cup.columbia.edu/reference/ciao>) |
| Final Search String | “hate speech” |
| Reported Yield | 11 |
| Actual Yield | 11 |
| Notes | Search Iteration:   1. (online radical* OR online terror* OR online extrem* OR “hate speech” ...) AND (extrem* narrative* OR racis* OR radical* OR dangerous speech OR ideology OR islamophobia OR homophobia OR transphobia OR misogyny OR disablism) AND (interven* OR option* OR strategy* OR “counter narrative*” ...) AND (comparison* OR quantitative OR qualitative OR quasi-experiment* OR survey* OR interview* ...) Results: NO Results 2. (online radical* OR online terror* OR online extrem* OR “hate speech” ...) AND (extrem* narrative* OR racis* OR radical* OR dangerous speech OR ideology OR islamophobia OR homophobia OR transphobia OR misogyny OR disablism) AND (interven* OR option* OR strategy* OR “counter narrative*” ...) Results: NO Results 3. (online radical* OR online terror* OR online extrem* OR “hate speech” ...) AND (extrem* narrative* OR racis* OR radical* OR dangerous speech OR ideology OR islamophobia OR homophobia OR transphobia OR misogyny OR disablism) Results: NO Results 4. (online radical* OR online terror* OR online extrem* OR “hate speech” ...) Results: NO Results 5. “hate speech” Results: 11   AMO pulled in the 11 items from the website. |
| Search date | 21 December 2020 |
| Initials | SMW |
| Database/Website/Journal/Strategy | Declassified Documents Reference System (<https://www.library.ucsb.edu/research/db/99>) |
| Final Search String | (hate speech AND internet AND campaign) |
| Reported Yield | 52 |
| Actual Yield | 0 |
| Notes | Performed search using terms above but did not pull down retrieved documents. Screened each study by title and abstract and planned to include only potentially relevant titles. After an exhaustive search, it does not appear that there are any scholarly articles available from this website. |
| Search date | 10 December 2020 |
| Initials | EMJ |
| Database/Website/Journal/Strategy | Don M. Gottfredson Library of Criminal Justice Gray Literature Database (<http://njlaw.rutgers.edu/cj/gray/search.php>) |
| Final Search String | hate speech in keyword |
| Reported Yield | 15 |
| Actual Yield | 15 |
| Notes | Searching for terms in full text and adding quotations, asterisks, and more than one line seemed to create problems for the search as a “problem running the query” statement would appear or it would not show any results without saying the 0 results or “problem running query”.  Search Iteration:   1. (online OR “social media” OR internet OR Twitter OR Facebook OR 8Chan OR 8Kun OR Gab OR Telegram OR TikTok OR Reddit OR WhatsApp OR Instagram OR “social networking site*” OR cybervictimization OR \”online incivility\”) AND (\”hate speech\” OR cyberhate OR extrem* narrative* OR racis* OR radical* OR speech OR ideolog* OR islamophobi* OR homophobi* OR transphobi* OR misogyny OR disablism OR discrim* OR terror*) AND (interven* OR option* OR strategy* OR “counter narrative*” OR “nudge” OR “norm* intervention” OR “norm* nudge” OR counternarrative* OR “alternative narrative*” OR campaign* OR counter* OR peer-to-peer OR prevent* OR disrupt* OR stop* OR fight* OR redirect* OR \”censoring hate content”) AND (comparison* OR quantitative OR quasi-experiment* OR survey* OR interview* OR poll* OR mixed-methods OR individual-level OR group-level OR control* OR experiment* OR study OR studies OR evaluat* OR MTurk OR longitudinal OR random* OR “digital method*” OR “machine learning” OR “natural language processing” OR multisectoral OR review*) in fulltext - yielded “Problem running the query” result 2. (online OR “social media” OR internet OR Twitter OR Facebook OR 8Chan OR 8Kun OR Gab OR Telegram OR TikTok OR Reddit OR WhatsApp OR Instagram OR “social networking site*” OR cybervictimization OR \”online incivility\”) in fulltext (\”hate speech\” OR cyberhate OR extrem* narrative* OR racis* OR radical* OR speech OR ideolog* OR islamophobi* OR homophobi* OR transphobi* OR misogyny OR disablism OR discrim* OR terror*) in fulltext (interven* OR option* OR strategy* OR “counter narrative*” OR “nudge” OR “norm* intervention” OR “norm* nudge” OR counternarrative* OR “alternative narrative*” OR campaign* OR counter* OR peer-to-peer OR prevent* OR disrupt* OR stop* OR fight* OR redirect* OR \”censoring hate content”) in fulltext (comparison* OR quantitative OR quasi-experiment* OR survey* OR interview* OR poll* OR mixed-methods OR individual-level OR group-level OR control* OR experiment* OR study OR studies OR evaluat* OR MTurk OR longitudinal OR random* OR “digital method*” OR “machine learning” OR “natural language processing” OR multisectoral OR review*) in fulltext - “Problem running the query” result 3. (online OR social media OR internet OR Twitter OR Facebook OR 8Chan OR 8Kun OR Gab OR Telegram OR TikTok OR Reddit OR WhatsApp OR Instagram OR social networking site OR cybervictimization OR online incivility) in fulltext (hate speech OR cyberhate OR extremist narrative OR extremism OR racism OR radical OR speech OR ideology OR islamophobia OR homophobia OR transphobia OR misogyny OR disablism OR discrimination OR terrorism) in fulltext (intervention OR option OR strategy OR counter narrative OR nudge OR norm intervention OR norm nudge OR counternarrative OR alternative narrative OR campaign OR counter OR peer-to- peer OR prevent OR disruption OR stop OR fight OR redirection OR censoring hate content) in fulltext (comparison OR quantitative OR quasi-experiment OR survey OR interview OR poll OR mixed- methods OR individual-level OR group-level OR control OR experiment OR study OR studies OR evaluation OR MTurk OR longitudinal OR random OR digital method OR machine learning OR natural language processing OR multisectoral OR review) in fulltext - yielded “Problem running the query” result 4. (online OR social media OR internet OR Twitter OR Facebook OR 8Chan OR 8Kun OR Gab OR Telegram OR TikTok OR Reddit OR WhatsApp OR Instagram OR social networking site OR cybervictimization OR online incivility) in title (hate speech OR cyberhate OR extremist narrative OR extremism OR racism OR radical OR speech OR ideology OR islamophobia OR homophobia OR transphobia OR misogyny OR disablism OR discrimination OR terrorism) in title (intervention OR option OR strategy OR counter narrative OR nudge OR norm intervention OR norm nudge OR counternarrative OR alternative narrative OR campaign OR counter OR peer-to- peer OR prevent OR disruption OR stop OR fight OR redirection OR censoring hate content) in title (comparison OR quantitative OR quasi-experiment OR survey OR interview OR poll OR mixed- methods OR individual-level OR group-level OR control OR experiment OR study OR studies OR evaluation OR MTurk OR longitudinal OR random OR digital method OR machine learning OR natural language processing OR multisectoral OR review) in title - yielded 0 results 5. (online OR social media OR internet OR Twitter OR Facebook OR 8Chan OR 8Kun OR Gab OR Telegram OR TikTok OR Reddit OR WhatsApp OR Instagram OR social networking site OR cybervictimization OR online incivility) in title (hate speech OR cyberhate OR extremist narrative OR extremism OR racism OR radical OR speech OR ideology OR islamophobia OR homophobia OR transphobia OR misogyny OR disablism OR discrimination OR terrorism) in title (intervention OR option OR strategy OR counter narrative OR nudge OR norm intervention OR norm nudge OR counternarrative OR alternative narrative OR campaign OR counter OR peer-to- peer OR prevent OR disruption OR stop OR fight OR redirection OR censoring hate content) in title - yielded 0 results 6. (online OR social media OR internet OR Twitter OR Facebook OR 8Chan OR 8Kun OR Gab OR Telegram OR TikTok OR Reddit OR WhatsApp OR Instagram OR social networking site OR cybervictimization OR online incivility) in title (hate speech OR cyberhate OR extremist narrative OR extremism OR racism OR radical OR speech OR ideology OR islamophobia OR homophobia OR transphobia OR misogyny OR disablism OR discrimination OR terrorism) in title - yielded 0 results 7. (online OR social media OR internet OR Twitter OR Facebook OR 8Chan OR 8Kun OR Gab OR Telegram OR TikTok OR Reddit OR WhatsApp OR Instagram OR social networking site OR cybervictimization OR online incivility) - yielded 0 results 8. (hate speech OR cyberhate OR extremist narrative OR extremism OR racism OR radical OR speech OR ideology OR islamophobia OR homophobia OR transphobia OR misogyny OR disablism OR discrimination OR terrorism) in title - yielded 0 results 9. hate speech in title online in title intervention in title - yielded 0 results 10. online hate speech in title - yielded 2 results 11. hate speech in keyword online in keyword - yielded 0 results 12. online hate speech in keyword - yielded 0 results 13. hate speech in keyword - yielded 15 results 14. hate speech in title - yielded 12 results |
| Search date | 23 November 2020 |
| Initials | HJC |
| Database/Website/Journal/Strategy | European Commission (<https://ec.europa.eu>) |
| Final Search String | “hate speech” cyberhate extrem* narrative* racis* radical* speech ideolog* islamophobi* misogyny terror* |
| Reported Yield | 1,007 |
| Actual Yield | 351 – issues with importing results and because many results were not relevant, results were screened and only those that appeared possibly relevant were imported |
| Notes | The basic search results did not yield many items on the European Commission website and when accessing the results it did yield, the results were linked to the EU publications database. Therefore, the search was run on the EU publications database: <https://op.europa.eu/en/home>. The EU Publications expert search function provided poor results. The advanced search works better but unfortunately has limited functionality and does not allow very long search strings. The advanced search was also limited because filtering by search field and by paper type (i.e. scholarly article or report) was not available, and the filters that were available were not particularly beneficial in reducing the yield. At the EU publications office home page, the following search strings were run as listed in the one search box under through the advanced search.   1. online “social media” internet Twitter Facebook 8Chan 8Kun Gab Telegram TikTok Reddit WhatsApp Instagram “social networking site*” cybervictimization “online incivility”   Results were filtered by searching for “any of these words”, searching only the “EU publications” collection, and setting the date range between 01/01/1990 to until 23/11/2020 (date of search). First search yield: 130,340 results   1. Used the “find in results” search function with the following search string...   “hate speech” cyberhate extrem* narrative* racis* radical* speech ideolog* islamophobi* misogyny terror*” –  This resulted in a more manageable second search yield of 22,885 results.   1. Filtered by the languages of English, German, Arabic, and Persian. Which resulted in a third search yield of 17,127 results. The first 500 items sorted by relevance and examined. Many were not relevant, so only the items identified as being relevant or possibly relevant by doing a quick sort by looking at the title, partial abstract, and subject were imported. 2. Fourth search yield: 1007   A fourth search was run just using the search string:  “hate speech” cyberhate extrem* narrative* racis* radical* speech ideolog* islamophobi* misogyny terror*  All of the items located in the third search were also in the results of the fourth search. For some reports, such as the individual “Country reports” on discrimination only the latest editions of these reports were imported, whereas there were several from different years. |
| Search date | 24 November 2020 |
| Initials | HJC |
| Database/Website/Journal/Strategy | Global Issues in Context (<https://www.gale.com/c/in-context-global-issues>) |
| Final Search String | Search limited to Basic Search field: (online OR “social media” OR internet OR Twitter OR Facebook OR 8Chan OR 8Kun OR Gab OR Telegram OR TikTok OR Reddit OR WhatsApp OR Instagram OR “social networking site*” OR cybervictimization OR “online incivility”) AND (“hate speech” OR cyberhate OR extrem* narrative* OR racis* OR radical* OR speech OR ideolog* OR islamophobi* OR homophobi* OR transphobi* OR misogyny OR disablism OR discrim* OR terror*) |
| Reported Yield | 184 |
| Actual Yield | 186 |
| Notes | The database was accessed through the University of Auckland library gateway. The search was completed using the “basic search” field, which meant that the results had “have these terms in the title, subject tagging, or first 50 words”. Results were filtered by date the dates 31/12/1989 and 24/12/2020. All the other filters were left blank. This database contains a wide range of resource types: academic journals, viewpoints, primary sources, reference, biographies, videos, audio, news, magazines, and websites. The overwhelming majority of search results were news articles. Only the items under “Academic Journals” were collected.  Search iterations:   1. First search yield: 25   Search limited to Basic Search: (online OR “social media” OR internet OR Twitter OR Facebook OR 8Chan OR 8Kun OR Gab OR Telegram OR TikTok OR Reddit OR WhatsApp OR Instagram OR “social networking site*” OR cybervictimization OR “online incivility”) AND (“hate speech” OR cyberhate OR extrem* narrative* OR racis* OR radical* OR speech OR ideolog* OR islamophobi* OR homophobi* OR transphobi* OR misogyny OR disablism OR discrim* OR terror*) AND (interven* OR option* OR strategy* OR “counter narrative*” OR “nudge” OR “norm* intervention” OR “norm* nudge” OR counternarrative* OR “alternative narrative*” OR campaign* OR counter* OR peer- to-peer OR prevent* OR disrupt* OR stop* OR fight* OR redirect* OR censoring hate content”) AND (comparison* OR quantitative OR quasi-experiment* OR survey* OR interview* OR poll* OR mixed- methods OR individual-level OR group-level OR control* OR experiment* OR study OR studies OR evaluat* OR MTurk OR longitudinal OR random* OR “digital method*” OR “machine learning” OR “natural language processing” OR multisectoral OR review*)   1. Second search yield: 45   Search limited to Basic Search: (online OR “social media” OR internet OR Twitter OR Facebook OR 8Chan OR 8Kun OR Gab OR Telegram OR TikTok OR Reddit OR WhatsApp OR Instagram OR “social networking site*” OR cybervictimization OR “online incivility”) AND (“hate speech” OR cyberhate OR extrem* narrative* OR racis* OR radical* OR speech OR ideolog* OR islamophobi* OR homophobi* OR transphobi* OR misogyny OR disablism OR discrim* OR terror*) AND (interven* OR option* OR strategy* OR “counter narrative*” OR “nudge” OR “norm* intervention” OR “norm* nudge” OR counternarrative* OR “alternative narrative*” OR campaign* OR counter* OR peer- to-peer OR prevent* OR disrupt* OR stop* OR fight* OR redirect* OR censoring hate content”)   1. Third search yield: 184   Search limited to Basic Search: (online OR “social media” OR internet OR Twitter OR Facebook OR 8Chan OR 8Kun OR Gab OR Telegram OR TikTok OR Reddit OR WhatsApp OR Instagram OR “social networking site*” OR cybervictimization OR “online incivility”) AND (“hate speech” OR cyberhate OR extrem* narrative* OR racis* OR radical* OR speech OR ideolog* OR islamophobi* OR homophobi* OR transphobi* OR misogyny OR disablism OR discrim* OR terror*) |
| Search date | 23 November 2020 |
| Initials | HN |
| Database/Website/Journal/Strategy | Google Scholar (<https://scholar.google.com/>) |
| Final Search String | (online OR “social media” OR internet OR Twitter OR Facebook OR 8Chan OR 8Kun OR Gab OR Telegram OR TikTok OR Reddit OR WhatsApp OR Instagram OR “social networking site*” OR cybervictimization OR “online incivility”) AND (“hate speech” OR cyberhate OR extrem* narrative* OR racis* OR radical* OR speech OR ideolog* OR islamophobi* OR homophobi* OR transphobi* OR misogyny OR disablism OR discrim* OR terror*) |
| Reported Yield | 25,200 |
| Actual Yield | 587 |
| Notes | Retrieved first 500 references sorted by relevance.  Search iterations:   1. (online OR “social media” OR internet OR Twitter OR Facebook OR 8Chan OR 8Kun OR Gab OR Telegram OR TikTok OR Reddit OR WhatsApp OR Instagram OR “social networking site*” OR cybervictimization OR “online incivility”) AND (“hate speech” OR cyberhate OR extrem* narrative* OR racis* OR radical* OR speech OR ideolog* OR islamophobi* OR homophobi* OR transphobi* OR misogyny OR disablism OR discrim* OR terror*) AND (interven* OR option* OR strategy* OR “counter narrative*” OR “nudge” OR “norm* intervention” OR “norm* nudge” OR counternarrative* OR “alternative narrative*” OR campaign* OR counter* OR peer-to-peer OR prevent* OR disrupt* OR stop* OR fight* OR redirect* OR “censoring hate content”) AND (comparison* OR quantitative OR quasi- experiment* OR survey* OR interview* OR poll* OR mixed-methods OR individual-level OR group-level OR control* OR experiment* OR study OR studies OR evaluat* OR MTurk OR longitudinal OR random* OR “digital method*” OR “machine learning” OR “natural language processing” OR multisectoral OR review*) Results: About 25,200 |
| Search date | 29 September – 2 October 2020; 5 – 7 October 2020 |
| Initials | EMJ |
| Database/Website/Journal/Strategy | Govinfo (<https://www.govinfo.gov/>) |
| Final Search String | (“online radical*” OR “online terror*” OR “online extrem*” OR “hate speech” OR cyberhate) AND (extrem* narrative* OR racis* OR radical*) AND (interven* OR option* OR strategy* OR counter*) |
| Reported Yield | 205 |
| Actual Yield | 200 |
| Notes | Date range: 1/1/1990 - 12/31/2020 and selected collection (Additional Government Publications, Congressional Committee Prints, Congressional Documents, Congressional Reports, Education Reports from ERIC, GAO Reports and Comptroller General Decisions). Have to manually add all publications Zotero. There were a few duplicates within the search which lead to the actual search yield being 5 less than the located search yield. All searches used the search field of full-text.  Search Iteration:   1. Date range: 1/1/1990 - 12/31/2020 and all collections with search terms: (online OR “social media” OR internet OR Twitter OR Facebook OR 8chan OR 8Kun OR Gab OR Telegram OR TikTok OR Reddit OR WhatsApp OR Instagram OR “social networking site*” OR “cybervictimization” OR “online incivility”) AND (“hate speech” OR cyberhate OR extrem* OR narrative OR racis* OR radical* OR speech OR ideolog* OR islamophobi* OR homophobi* OR transphobi* OR misogyny OR disablism OR discrim* OR terror*) AND (interven* OR option* OR strategy* OR “counter narrative*” OR “nudge” OR “norm* intervention” OR “norm* nudge” OR counternarrative* OR “alternative narrative*” OR campaign* OR counter* OR peer-to-peer OR prevent* OR disrupt* OR stop* OR fight* OR redirect* OR “censoring hate content”) AND (comparison* OR quantitative OR quasi-experiment* OR survey* OR interview* OR poll* OR mixed-methods OR individual-level OR group-level OR control* OR experiment* OR study OR studies OR evaluat* OR MTurk OR longitudinal OR random* OR “digital method*” OR “machine learning” OR “natural language processing” OR multisectoral OR review*) – yielded 0 results 2. Date range: 1/1/1990 - 12/31/2020 and all collections with search terms: (“online radical*” OR “online terror*” OR “online extrem*” OR “hate speech” OR cyberhate OR “social media” OR internet OR Twitter OR Facebook OR 8Chan OR 8Kun OR Gab OR Telegram OR TikTok OR Reddit OR WhatsApp OR Instagram OR “social networking site*”) AND (extrem* narrative* OR racis* OR radical* OR “dangerous speech” OR ideology OR islamophobia OR homophobia OR transphobia OR misogyny OR disablism) AND (interven* OR option* OR strategy* OR “counter narrative*” OR counternarrative* OR “alternative narrative*” OR campaign* OR counter* OR peer-to-peer OR prevent* OR disrupt* OR stop* OR fight* OR redirect*) AND (comparison* OR quantitative OR qualitative OR quasi-experiment* OR survey* OR interview* OR poll* OR mixed-methods OR individual-level OR group-level OR control* OR experiment* OR study OR studies OR evaluat* OR MTurk OR longitudinal OR random* OR “digital method*” OR “machine learning” OR “natural language processing” OR multisectoral OR review*) – yielded 0 results 3. Date range: 1/1/1990 - 12/31/2020 and all collections with search terms: (“online radical*” OR “online terror*” OR “online extrem*” OR “hate speech” OR cyberhate OR “social media” OR internet OR Twitter OR Facebook OR 8Chan OR 8Kun OR Gab OR Telegram OR TikTok OR Reddit OR WhatsApp OR Instagram OR “social networking site*”) AND (extrem* narrative* OR racis* OR radical* OR “dangerous speech” OR ideology OR islamophobia OR homophobia OR transphobia OR misogyny OR disablism) AND (interven* OR option* OR strategy* OR “counter narrative*” OR counternarrative* OR “alternative narrative*” OR campaign* OR counter* OR peer-to-peer OR prevent* OR disrupt* OR stop* OR fight* OR redirect*) – yielded 33,148 results 4. Date range: 1/1/1990 - 12/31/2020 and all collections with search terms: (“online radical*” OR “online terror*” OR “online extrem*” OR “hate speech” OR cyberhate OR “social media” OR internet OR Twitter OR Facebook OR 8Chan OR 8Kun OR Gab OR Telegram OR TikTok OR Reddit OR WhatsApp OR Instagram OR “social networking site*”) AND (extrem* narrative* OR racis* OR radical* OR “dangerous speech” OR ideology OR islamophobia OR homophobia OR transphobia OR misogyny OR disablism) – yielded 33,879 results 5. Date range: 1/1/1990 - 12/31/2020 and all collections with search terms: (“online radical*” OR “online terror*” OR “online extrem*” OR “hate speech” OR cyberhate OR “social media” OR internet OR Twitter OR Facebook OR 8Chan OR 8Kun OR Gab OR Telegram OR TikTok OR Reddit OR WhatsApp OR Instagram OR “social networking site*”) – yielded 458,256 results 6. Date range: 1/1/1990 - 12/31/2020 and all collections with search terms: (“online radical*” OR “online terror*” OR “online extrem*” OR “hate speech” OR cyberhate) AND (extrem* narrative* OR racis* OR radical*) AND (interven* OR option* OR strategy* OR counter*) – yielded 7,913 results 7. Date range: 1/1/1990 - 12/31/2020 and all collections with search terms: (“hate speech”) AND (online) AND (radical) AND (“social media”) AND (study) - yielded 222 results 8. Date range: 1/1/1990 - 12/31/2020 and selected collection (Additional Government Publications, Congressional Committee Prints, Congressional Documents, Congressional Reports, Education Reports from ERIC, GAO Reports and Comptroller General Decisions) with search terms: (“online radical*” OR “online terror*” OR “online extrem*” OR “hate speech” OR cyberhate) AND (extrem* narrative* OR racis* OR radical*) AND (interven* OR option* OR strategy* OR counter*) – yielded 205 results |
| Search date | 16 November 2020 |
| Initials | HJC |
| Database/Website/Journal/Strategy | HeinOnline (<https://home.heinonline.org/>) |
| Final Search String | Search limited to Article Title/Section Title: (online OR “social media” OR internet OR Twitter OR Facebook OR 8Chan OR 8Kun OR Gab OR Telegram OR TikTok OR Reddit OR WhatsApp OR Instagram OR “social networking site*” OR cybervictimization OR “online incivility”) AND (“hate speech” OR cyberhate OR extrem* narrative* OR racis* OR radical* OR speech OR ideolog* OR islamophobi* OR homophobi* OR transphobi* OR misogyny OR disablism OR discrim* OR terror*) |
| Reported Yield | 535 |
| Actual Yield | 531 |
| Notes | Used the search field “Text”/”Full Text” for the first few searches with the date range 1989 - 2021. All “Section Types” were left at the default - which meant “Contents” was unchecked. It also meant that “Include external articles (articles outside of HeinOnline) in the search results” was checked. All searches were sorted by the default “Relevance”. Some of the items did not have authors as they were from law journals with no listed author, others had no authors as they were transcripts of forums or symposiums and had no listed author. All items were collected apart from 4 duplicates in the HeinOnline search results.  Search iterations:   1. yield: 0   (online OR “social media” OR internet OR Twitter OR Facebook OR 8Chan OR 8Kun OR Gab OR Telegram OR TikTok OR Reddit OR WhatsApp OR Instagram OR “social networking site*” OR cybervictimization OR “online incivility”) AND (“hate speech” OR cyberhate OR extrem* narrative* OR racis* OR radical* OR speech OR ideolog* OR islamophobi* OR homophobi* OR transphobi* OR misogyny OR disablism OR discrim* OR terror*) AND (interven* OR option* OR strategy* OR “counter narrative*” OR “nudge” OR “norm* intervention” OR “norm* nudge” OR counternarrative* OR “alternative narrative*” OR campaign* OR counter* OR peer- to-peer OR prevent* OR disrupt* OR stop* OR fight* OR redirect* OR censoring hate content”) AND (comparison* OR quantitative OR quasi-experiment* OR survey* OR interview* OR poll* OR mixed- methods OR individual-level OR group-level OR control* OR experiment* OR study OR studies OR evaluat* OR MTurk OR longitudinal OR random* OR “digital method*” OR “machine learning” OR “natural language processing” OR multisectoral OR review*)   1. yield: 0   (online OR “social media” OR internet OR Twitter OR Facebook OR 8Chan OR 8Kun OR Gab OR Telegram OR TikTok OR Reddit OR WhatsApp OR Instagram OR “social networking site*” OR cybervictimization OR “online incivility”) AND (“hate speech” OR cyberhate OR extrem* narrative* OR racis* OR radical* OR speech OR ideolog* OR islamophobi* OR homophobi* OR transphobi* OR misogyny OR disablism OR discrim* OR terror*) AND (interven* OR option* OR strategy* OR “counter narrative*” OR “nudge” OR “norm* intervention” OR “norm* nudge” OR counternarrative* OR “alternative narrative*” OR campaign* OR counter* OR peer- to-peer OR prevent* OR disrupt* OR stop* OR fight* OR redirect* OR censoring hate content”)   1. yield: 439,830   (online OR “social media” OR internet OR Twitter OR Facebook OR 8Chan OR 8Kun OR Gab OR Telegram OR TikTok OR Reddit OR WhatsApp OR Instagram OR “social networking site*” OR cybervictimization OR “online incivility”) AND (“hate speech” OR cyberhate OR extrem* narrative* OR racis* OR radical* OR speech OR ideolog* OR islamophobi* OR homophobi* OR transphobi* OR misogyny OR disablism OR discrim* OR terror*)  For the following search queries, changed the field from “Text” to “Article Title”   1. yield: 0   (online OR “social media” OR internet OR Twitter OR Facebook OR 8Chan OR 8Kun OR Gab OR Telegram OR TikTok OR Reddit OR WhatsApp OR Instagram OR “social networking site*” OR cybervictimization OR “online incivility”) AND (“hate speech” OR cyberhate OR extrem* narrative* OR racis* OR radical* OR speech OR ideolog* OR islamophobi* OR homophobi* OR transphobi* OR misogyny OR disablism OR discrim* OR terror*) AND (interven* OR option* OR strategy* OR “counter narrative*” OR “nudge” OR “norm* intervention” OR “norm* nudge” OR counternarrative* OR “alternative narrative*” OR campaign* OR counter* OR peer- to-peer OR prevent* OR disrupt* OR stop* OR fight* OR redirect* OR censoring hate content”) AND (comparison* OR quantitative OR quasi-experiment* OR survey* OR interview* OR poll* OR mixed- methods OR individual-level OR group-level OR control* OR experiment* OR study OR studies OR evaluat* OR MTurk OR longitudinal OR random* OR “digital method*” OR “machine learning” OR “natural language processing” OR multisectoral OR review*)   1. yield: 0   (online OR “social media” OR internet OR Twitter OR Facebook OR 8Chan OR 8Kun OR Gab OR Telegram OR TikTok OR Reddit OR WhatsApp OR Instagram OR “social networking site*” OR cybervictimization OR “online incivility”) AND (“hate speech” OR cyberhate OR extrem* narrative* OR racis* OR radical* OR speech OR ideolog* OR islamophobi* OR homophobi* OR transphobi* OR misogyny OR disablism OR discrim* OR terror*) AND (interven* OR option* OR strategy* OR “counter narrative*” OR “nudge” OR “norm* intervention” OR “norm* nudge” OR counternarrative* OR “alternative narrative*” OR campaign* OR counter* OR peer- to-peer OR prevent* OR disrupt* OR stop* OR fight* OR redirect* OR censoring hate content”)   1. yield: 535 (515 + 20 results “from Index to Foreign Legal Periodicals”)   (online OR “social media” OR internet OR Twitter OR Facebook OR 8Chan OR 8Kun OR Gab OR Telegram OR TikTok OR Reddit OR WhatsApp OR Instagram OR “social networking site*” OR cybervictimization OR “online incivility”) AND (“hate speech” OR cyberhate OR extrem* narrative* OR racis* OR radical* OR speech OR ideolog* OR islamophobi* OR homophobi* OR transphobi* OR misogyny OR disablism OR discrim* OR terror*) |
| Search date | 8 – 11 September 2020; 16 – 18 September 2020; 21 – 23 September 2020 |
| Initials | EMJ |
| Database/Website/Journal/Strategy | Homeland Security Digital Library (HSDL) (<https://www.hsdl.org/c/>) |
| Final Search String | See notes |
| Reported Yield | 651 |
| Actual Yield | 616 |
| Notes | Search in “all of these words” box: online radical social media counter study - in “exact phrase” box hate speech   - Restrictions: Year, 01/01/1990 – 31/12/2020   Have to manually add all publications Zotero. Several publications were no longer available or were entire journals, so the actual yield was different then the located yield. A few of the links to the documents brought up an Error:404 page. Therefore, google was used to search the host website for that document. Several were no longer available, the rest were brought into the Zotero folder.  Search Iteration:   1. First search in “any of these words” box: (“online radical*” OR “online terror*” OR “online extrem*” OR “hate speech” OR cyberhate OR “social media” OR internet OR Twitter OR Facebook OR 8Chan OR 8Kun OR Gab OR Telegram OR TikTok OR Reddit OR WhatsApp OR Instagram OR “social networking site*”) AND (extrem* narrative* OR racis* OR radical* OR “dangerous speech” OR ideology OR islamophobia OR homophobia OR transphobia OR misogyny OR disablism) AND (interven* OR option* OR strategy* OR “counter narrative*” OR counternarrative* OR “alternative narrative*” OR campaign* OR counter* OR peer-to-peer OR prevent* OR disrupt* OR stop* OR fight* OR redirect*) AND (comparison* OR quantitative OR qualitative OR quasi- experiment* OR survey* OR interview* OR poll* OR mixed-methods OR individual-level OR group-level OR control* OR experiment* OR study OR studies OR evaluat* OR MTurk OR longitudinal OR random* OR “digital method*” OR “machine learning” OR “natural language processing” OR multisectoral OR review*) - yielded 189,927 results 2. Second search in “any of these words” box: “online radical*” “online terror*” “online extrem*” “hate speech” cyberhate “social media” internet Twitter Facebook 8Chan 8Kun Gab Telegram TikTok Reddit WhatsApp Instagram “social networking site*” - yielded 153,265 results 3. Third search in “any of these words” box: online radical* terror* extrem* hate speech cyberhate social media internet - yielded 154,746 results 4. Fourth search in “all of these words” box: hate speech social media counternarrative - yielded 119 results 5. Fifth search in “all of these words” box: hate speech social media counter - yielded 3,959 results 6. Sixth search in “all of these words” box: hate speech social media counter* OR interven* - yielded 1,974 results 7. Seventh search in “all of these words” box: social media counter study - in “exact phrase” box hate speech - yielded 921 results 8. Eighth search in “all of these words” box: online social media counter study - in “exact phrase” box hate speech - yielded 819 results 9. Ninth search in “all of these words” box: online cyberhate social media counter study - in “exact phrase” box hate speech - yielded 38 results 10. Tenth search in “all of these words” box: online radical social media counter study - in “exact phrase” box hate speech - yielded 651 results |
| Search date | 17 August 2020; 24 November 2020 |
| Initials | SW |
| Database/Website/Journal/Strategy | Index New Zealand: INNZ (<https://natlib.govt.nz/collections/a-z/index-new-zealand-innz>) |
| Final Search String | Search limited to All Fields: (online OR “social media” OR internet OR Twitter OR Facebook OR 8Chan OR 8Kun OR Gab OR Telegram OR TikTok OR Reddit OR WhatsApp OR Instagram OR “social networking site*” OR cybervictimization OR “online incivility”) AND (“hate speech” OR cyberhate OR extrem* narrative* OR racis* OR radical* OR speech OR ideolog*) |
| Reported Yield | 925 |
| Actual Yield | 925 |
| Notes | Restrictions:   - Final search excludes newspaper articles (104) = 925   All searches used search field “all fields”  Search iterations:   1. Full string: (online OR “social media” OR internet OR Twitter OR Facebook OR 8Chan OR 8Kun OR Gab OR Telegram OR TikTok OR Reddit OR WhatsApp OR Instagram OR “social networking site*” OR cybervictimization OR “online incivility”) AND (“hate speech” OR cyberhate OR extrem* narrative* OR racis* OR radical* OR speech OR ideolog* OR islamophobi* OR homophobi* OR transphobi* OR misogyny OR disablism OR discrim* OR terror*) AND (interven* OR option* OR strategy* OR “counter narrative*” OR “nudge” OR “norm* intervention” OR “norm* nudge” OR counternarrative* OR “alternative narrative*” OR campaign* OR counter* OR peer-to-peer OR prevent* OR disrupt* OR stop* OR fight* OR redirect* OR “censoring hate content”) AND (comparison* OR quantitative OR quasi-experiment* OR survey* OR interview* OR poll* OR mixed-methods OR individual-level OR group-level OR control* OR experiment* OR study OR studies OR evaluat* OR MTurk OR longitudinal OR random* OR “digital method*” OR “machine learning” OR “natural language processing” OR multisectoral OR review*) – *too many wildcards* 2. First three: (online OR “social media” OR internet OR Twitter OR Facebook OR 8Chan OR 8Kun OR Gab OR Telegram OR TikTok OR Reddit OR WhatsApp OR Instagram OR “social networking site*” OR cybervictimization OR “online incivility”) AND (“hate speech” OR cyberhate OR extrem* narrative* OR racis* OR radical* OR speech OR ideolog* OR islamophobi* OR homophobi* OR transphobi* OR misogyny OR disablism OR discrim* OR terror*) AND (interven* OR option* OR strategy* OR “counter narrative*” OR “nudge” OR “norm* intervention” OR “norm* nudge” OR counternarrative* OR “alternative narrative*” OR campaign* OR counter* OR peer-to-peer OR prevent* OR disrupt* OR stop* OR fight* OR redirect* OR “censoring hate content”) – *too many wildcards* 3. First two: (online OR “social media” OR internet OR Twitter OR Facebook OR 8Chan OR 8Kun OR Gab OR Telegram OR TikTok OR Reddit OR WhatsApp OR Instagram OR “social networking site*” OR cybervictimization OR “online incivility”) AND (“hate speech” OR cyberhate OR extrem* narrative* OR racis* OR radical* OR speech OR ideolog* OR islamophobi* OR homophobi* OR transphobi* OR misogyny OR disablism OR discrim* OR terror*) – *too many wildcards* 4. First one: (online OR “social media” OR internet OR Twitter OR Facebook OR 8Chan OR 8Kun OR Gab OR Telegram OR TikTok OR Reddit OR WhatsApp OR Instagram OR “social networking site*” OR cybervictimization OR “online incivility”) – 36,326 results 5. First part + partial third part: (online OR “social media” OR internet OR Twitter OR Facebook OR 8Chan OR 8Kun OR Gab OR Telegram OR TikTok OR Reddit OR WhatsApp OR Instagram OR “social networking site*” OR cybervictimization OR “online incivility”) AND (interven* OR counter* OR prevent* OR “nudge”) – 1,553 6. (online OR “social media” OR internet OR Twitter OR Facebook OR 8Chan OR 8Kun OR Gab OR Telegram OR TikTok OR Reddit OR WhatsApp OR Instagram OR “social networking site*” OR cybervictimization OR “online incivility”) AND (“hate speech” OR cyberhate OR extrem* narrative* OR racis* OR radical* OR speech OR ideolog*) - 1,029 excluding newspaper articles (-104) – yields 925 |
| Search date | 17 December 2020 |
| Initials | HJC |
| Database/Website/Journal/Strategy | Ingenta Connect (<https://www.ingentaconnect.com/>) |
| Final Search String | Search limited to Article title, keywords or abstract: (“hate speech” OR cyberhate OR extrem* narrative* OR racis* OR radical* OR speech OR ideolog* OR islamophobi* OR homophobi* OR transphobi* OR misogyny OR disablism OR discrim* OR terror*) |
| Reported Yield | 1,442 |
| Actual Yield | 1,336 |
| Notes | The search function only allowed a limited number of characters and did not allow additional lines so each of the string tiers were searched separately. Used advance search – which was limited in the number of characters it allowed to run, and therefore, was restricted to running the individual search strings below.   1. First search yield: 6   Search limited to Article title, keywords or abstract: (online OR “social media” OR internet OR Twitter OR Facebook OR 8Chan OR 8Kun OR Gab OR Telegram OR TikTok OR Reddit OR WhatsApp OR Instagram OR “social networking site*” OR cybervictimization OR “online incivility”)   1. Second search yield: 1442   Search limited to Article title, keywords or abstract: (“hate speech” OR cyberhate OR extrem* narrative* OR racis* OR radical* OR speech OR ideolog* OR islamophobi* OR homophobi* OR transphobi* OR misogyny OR disablism OR discrim* OR terror*) |
| Search date | 28 December 2020 |
| Initials | SMW |
| Database/Website/Journal/Strategy | Journals@Ovid (<https://ovidsp-dc2-ovid-com.libproxy.temple.edu/>) |
| Final Search String | (internet OR hate speech OR cyberhate OR narrative OR radical OR counternarrative OR censoring hate content) |
| Reported Yield  Actual Yield  Notes | 4,808  547  Retrieved first ~500 references sorted by relevance.  Year restriction: 1990-2020  Search iterations: The search string did not allow for any special characters such as quotation marks or asterisks or paratheses.   1. Search limited to titles: (online OR social media OR internet OR Twitter OR Facebook OR 8Chan OR 8Kun OR Gab OR Telegram OR TikTok OR Reddit OR WhatsApp OR Instagram OR social networking site OR cybervictimization OR online incivility AND hate speech OR cyberhate OR narrative OR radical OR speech OR misogyny OR disablism OR terror AND option OR nudge OR norm intervention OR norm nudge OR counternarrative OR alternative narrative OR peer-to-peer OR prevent OR disrupt OR stop OR fight OR redirect OR censoring hate content AND comparison OR quantitative OR quasi- experiment OR survey OR interview OR poll OR mixed-methods OR individual-level OR group-level OR control OR experiment OR study OR studies OR MTurk OR longitudinal OR random OR digital method OR machine learning OR natural language processing OR multisectoral OR review)   Yield: 7,119,675   1. search limited to titles: (online OR social media OR internet OR social networking site OR cybervictimization OR online incivility AND hate speech OR cyberhate OR narrative OR radical OR speech OR misogyny OR disablism OR terror AND option OR nudge OR norm intervention OR norm nudge OR counternarrative OR alternative narrative OR peer-to-peer OR prevent OR disrupt OR stop OR fight OR redirect OR censoring hate content)   Yield: 24,826   1. search limited to titles and articles with abstracts: (internet OR social networking site OR cybervictimization OR online incivility AND hate speech OR cyberhate OR narrative OR radical OR speech OR counternarrative OR alternative narrative OR censoring hate content)   Yield: 14,902   1. search limited to abstract: (internet OR hate speech OR cyberhate OR narrative OR radical OR counternarrative OR censoring hate content)   Yield: 4,808 |
| Search date | 15 December 2020 |
| Initials | SMW |
| Database/Website/Journal/Strategy | JSTOR (<https://www.jstor.org/>) |
| Final Search String | (Internet AND hate speech AND intervention AND campaign AND counter) |
| Reported Yield | 723 |
| Actual Yield | 545 |
| Notes | JSTOR limits the search string to 200 characters.  Retrieved first ~500 references sorted by relevance.  Search iterations:   1. Located Yield = 106,633   (“online radical*” OR “online terror*” OR “online extrem*” OR “hate speech” OR cyberhate OR “social media” OR internet) AND (extrem* narrative* OR racis* OR radical*)   1. Located Yield = 341,366   (“online radical*” OR “online terror*” OR “online extrem*” OR “hate speech” OR cyberhate OR “social media” OR internet)   1. Located Yield = 723   (internet AND hate speech AND intervention AND campaign AND counter) |
| Search date | 15 December 2020; 27 December 2020 |
| Initials | AMO, SMW |
| Database/Website/Journal/Strategy | LLMC Digital |
| Final Search String | Hate speech |
| Reported Yield | 93 |
| Actual Yield | 0 |
| Notes | Search iterations:   1. Advanced search, wildcard: (online OR “social media” OR internet OR Twitter OR Facebook OR 8Chan OR 8Kun OR Gab OR Telegram OR TikTok OR Reddit OR WhatsApp OR Instagram OR “social networking site*” OR cybervictimization OR “online incivility”)   AND  (“hate speech” OR cyberhate OR extrem* narrative* OR racis* OR radical* OR speech OR ideolog* OR islamophobi* OR homophobi* OR transphobi* OR misogyny OR disablism OR discrim* OR terror*)  AND  (interven* OR option* OR strategy* OR “counter narrative*” OR “nudge” OR “norm* intervention” OR “norm* nudge” OR counternarrative* OR “alternative narrative*” OR campaign* OR counter* OR peer- to-peer OR prevent* OR disrupt* OR stop* OR fight* OR redirect* OR censoring hate content”); yield = 0   1. Advanced search, exact: (online OR “social media” OR internet OR Twitter OR Facebook OR 8Chan OR 8Kun OR Gab OR Telegram OR TikTok OR Reddit OR WhatsApp OR Instagram OR “social networking site*” OR cybervictimization OR “online incivility”)   AND  (“hate speech” OR cyberhate OR extrem* narrative* OR racis* OR radical* OR speech OR ideolog* OR islamophobi* OR homophobi* OR transphobi* OR misogyny OR disablism OR discrim* OR terror*)  AND  (interven* OR option* OR strategy* OR “counter narrative*” OR “nudge” OR “norm* intervention” OR “norm* nudge” OR counternarrative* OR “alternative narrative*” OR campaign* OR counter* OR peer- to-peer OR prevent* OR disrupt* OR stop* OR fight* OR redirect* OR censoring hate content”); yield = 0   1. Advanced search, exact: hate speech AND online AND intervention; yield = 0 2. Advanced search, exact: hate speech; yield = 93   Note: Performed search using terms above but could not pull documents into Zotero. Thus, each study was screened by title and abstract and planned to only include potentially relevant titles. After an exhaustive search, it does not appear that there are any scholarly articles available from this website. |
| Search date | 1 December 2020 |
| Initials | HJC |
| Database/Website/Journal/Strategy | Multicultural Australia and Immigration Studies - Aboriginal and Torres Strait Islander Subset (MAIS-ATSIS) (<https://www.informit.org/index-product-details/MAIS>) |
| Final Search String | Search field set to “All fields” and “All terms”: (online OR “social media” OR internet OR Twitter OR Facebook OR 8Chan OR 8Kun OR Gab OR Telegram OR TikTok OR Reddit OR WhatsApp OR Instagram OR “social networking site*” OR cybervictimization OR “online incivility”) AND (“hate speech” OR cyberhate OR extrem* narrative* OR racis* OR radical* OR speech OR ideolog* OR islamophobi* OR homophobi* OR transphobi* OR misogyny OR disablism OR discrim* OR terror*) AND (interven* OR option* OR strategy* OR “counter narrative*” OR “nudge” OR “norm* intervention” OR “norm* nudge” OR counternarrative* OR “alternative narrative*” OR campaign* OR counter* OR peer- to-peer OR prevent* OR disrupt* OR stop* OR fight* OR redirect* OR censoring hate content”) AND (comparison* OR quantitative OR quasi-experiment* OR survey* OR interview* OR poll* OR mixed- methods OR individual-level OR group-level OR control* OR experiment* OR study OR studies OR evaluat* OR MTurk OR longitudinal OR random* OR “digital method*” OR “machine learning” OR “natural language processing” OR multisectoral OR review*) |
| Reported Yield | 1,811 |
| Actual Yield | 1,000 |
| Notes | The database was accessed through the University of Auckland library website (as it requires access through an institution). The advanced search option was used, searched with the default “All Fields” and “All terms”. Year range 1989 - 2021. Retrieved first 1000 references sorted by relevance.   1. First search yield: 1811   (online OR “social media” OR internet OR Twitter OR Facebook OR 8Chan OR 8Kun OR Gab OR Telegram OR TikTok OR Reddit OR WhatsApp OR Instagram OR “social networking site*” OR cybervictimization OR “online incivility”) AND (“hate speech” OR cyberhate OR extrem* narrative* OR racis* OR radical* OR speech OR ideolog* OR islamophobi* OR homophobi* OR transphobi* OR misogyny OR disablism OR discrim* OR terror*) AND (interven* OR option* OR strategy* OR “counter narrative*” OR “nudge” OR “norm* intervention” OR “norm* nudge” OR counternarrative* OR “alternative narrative*” OR campaign* OR counter* OR peer- to-peer OR prevent* OR disrupt* OR stop* OR fight* OR redirect* OR censoring hate content”) AND (comparison* OR quantitative OR quasi-experiment* OR survey* OR interview* OR poll* OR mixed- methods OR individual-level OR group-level OR control* OR experiment* OR study OR studies OR evaluat* OR MTurk OR longitudinal OR random* OR “digital method*” OR “machine learning” OR “natural language processing” OR multisectoral OR review*) – I pulled the first 1000 sorted by relevance. |
| Search date | 13 November 2020; 18 November 2020; 22 November2020 |
| Initials | EMJ |
| Database/Website/Journal/Strategy | Oxford Journals Online (<https://academic.oup.com/journals/>) |
| Final Search String | Search field set to All: online AND hate speech AND intervention AND study |
| Reported Yield | 422 |
| Actual Yield | 422 |
| Notes | Restrictions:   - Article type: Research Article - Published: January 1990 to December 2020   Search field was set to “all” which includes abstract, full text, keywords, and title.  Search iterations:   1. tried to search whole search string and website kept crashing or would say 0 2. (online OR “social media” OR internet OR Twitter OR Facebook OR 8Chan OR 8Kun OR Gab OR Telegram OR TikTok OR Reddit OR WhatsApp OR Instagram OR “social networking site*” OR cybervictimization OR “online incivility”) – yielded 1,184,659 results 3. (online OR “social media” OR internet) AND (“hate speech” OR cyberhate OR extrem* narrative* OR radical* OR ideolog* OR discrim* OR terror*) AND (interven* OR option* OR strategy* OR “counter narrative*”) AND (comparison* OR study OR evaluat* OR review*) – yielded 0 results 4. (online OR “social media” OR internet) AND (“hate speech” OR cyberhate OR extrem* narrative* OR radical* OR ideolog* OR discrim* OR terror*) AND (interven* OR option* OR strategy* OR “counter narrative*”) – yielded 0 results 5. (online OR “social media” OR internet) AND (“hate speech” OR cyberhate OR extrem* narrative* OR radical* OR ideolog* OR discrim* OR terror*) – yielded 0 results 6. (online OR “social media” OR internet) – yielded 0 results 7. (online OR “social media” OR internet) – yielded 1,049,692 results 8. (“hate speech” OR cyberhate OR extrem* narrative* OR racis* OR radical* OR speech OR ideolog* OR islamophobi* OR homophobi* OR transphobi* OR misogyny OR disablism OR discrim* OR terror*) Journal articles format – yielded 915,272 results 9. online AND hate speech AND intervention AND study – yielded 421 results |
| Search date | 15 – 16 October 2020; 13 November 2020; 22 December 2020 |
| Initials | EMJ |
| Database/Website/Journal/Strategy | Oxford Scholarship Online (<https://oxford.universitypressscholarship.com/>) |
| Final Search String | (online OR “social media” OR internet OR Twitter OR Facebook OR 8Chan OR 8Kun OR Gab OR Telegram OR TikTok OR Reddit OR WhatsApp OR Instagram OR “social networking site*” OR cybervictimization OR “online incivility”) AND (“hate speech” OR cyberhate OR extrem* narrative* OR racis* OR radical* OR speech OR ideolog* OR islamophobi* OR homophobi* OR transphobi* OR misogyny OR disablism OR discrim* OR terror*) |
| Reported Yield | 119 |
| Actual Yield | 119 |
| Notes | The entire search string was put into the one search bar at the top of the page. This was a basic search and only looked at Oxford Scholarship Online, it did not include all partner presses. Because this was a basic search, the search fields used by the database were not specified. When each iteration was put as a new search term under advanced search, tens of thousands results are produced. The results were limited to unlocked, free and open access availability.  Search Iteration:   1. (online OR “social media” OR internet OR Twitter OR Facebook OR 8Chan OR 8Kun OR Gab OR Telegram OR TikTok OR Reddit OR WhatsApp OR Instagram OR “social networking site*” OR cybervictimization OR “online incivility”) AND (“hate speech” OR cyberhate OR extrem* narrative* OR racis* OR radical* OR speech OR ideolog* OR islamophobi* OR homophobi* OR transphobi* OR misogyny OR disablism OR discrim* OR terror*) – yielded 119 results |
| Search date | 28 – 30 October 2020 |
| Initials | EMJ |
| Database/Website/Journal/Strategy | Project Muse (<https://muse.jhu.edu/>) |
| Final Search String | (“hate speech” OR terror OR radical OR extremism OR extremist) AND (counter) AND (online) |
| Reported Yield | 226 |
| Actual Yield | 226 |
| Notes | The first 5 search terms were included in brackets in one search bar and the other two terms were in their own search bars. The search field was set to content. All 226 results were imported.  Search Iteration:   1. (“online radical*” OR “online terror*” OR “online extrem*” OR “hate speech” OR cyberhate OR “social media” OR internet OR Twitter OR Facebook OR 8Chan OR 8Kun OR Gab OR Telegram OR TikTok OR Reddit OR WhatsApp OR Instagram OR “social networking site*”) AND (extrem* narrative* OR racis* OR radical* OR “dangerous speech” OR ideology OR islamophobia OR homophobia OR transphobia OR misogyny OR disablism) AND (interven* OR option* OR strategy* OR “counter narrative*” OR counternarrative* OR “alternative narrative*” OR campaign* OR counter* OR peer-to-peer OR prevent* OR disrupt* OR stop* OR fight* OR redirect*) AND (comparison* OR quantitative OR qualitative OR quasi-experiment* OR survey* OR interview* OR poll* OR mixed-methods OR individual-level OR group-level OR control* OR experiment* OR study OR studies OR evaluat* OR MTurk OR longitudinal OR random* OR “digital method*” OR “machine learning” OR “natural language processing” OR multisectoral OR review*) – yielded 0 results 2. (“online radical*” OR “online terror*” OR “online extrem*” OR “hate speech” OR cyberhate OR “social media” OR internet OR Twitter OR Facebook OR 8Chan OR 8Kun OR Gab OR Telegram OR TikTok OR Reddit OR WhatsApp OR Instagram OR “social networking site*”) AND (extrem* narrative* OR racis* OR radical* OR “dangerous speech” OR ideology OR islamophobia OR homophobia OR transphobia OR misogyny OR disablism) AND (interven* OR option* OR strategy* OR “counter narrative*” OR counternarrative* OR “alternative narrative*” OR campaign* OR counter* OR peer-to-peer OR prevent* OR disrupt* OR stop* OR fight* OR redirect*) – yielded 0 results 3. (“online radical*” OR “online terror*” OR “online extrem*” OR “hate speech” OR cyberhate OR “social media” OR internet OR Twitter OR Facebook OR 8Chan OR 8Kun OR Gab OR Telegram OR TikTok OR Reddit OR WhatsApp OR Instagram OR “social networking site*”) AND (extrem* narrative* OR racis* OR radical* OR “dangerous speech” OR ideology OR islamophobia OR homophobia OR transphobia OR misogyny OR disablism) – yielded 0 results 4. (“online radical*” OR “online terror*” OR “online extrem*” OR “hate speech” OR cyberhate OR “social media” OR internet OR Twitter OR Facebook OR 8Chan OR 8Kun OR Gab OR Telegram OR TikTok OR Reddit OR WhatsApp OR Instagram OR “social networking site*”) – yielded 0 results 5. “hate speech” – yielded 2139 results 6. (“hate speech” OR terror OR radical OR extremism OR extremist) AND (counter) AND (online) – yielded 226 results |
| Search date | 2 December 2020 |
| Initials | EL |
| Database/Website/Journal/Strategy | PsychiatryOnline (<https://psychiatryonline.org/>) |
| Final Search String | (online radical* OR online terror* OR online extrem* OR “hate speech” OR cyberhate OR “social media” OR internet OR Twitter OR Facebook OR 8Chan OR 8Kun OR Gab OR Telegram OR TikTok OR Reddit OR WhatsApp OR Instagram OR “social networking site*”) |
| Reported Yield | 1,507 |
| Actual Yield | 539 |
| Notes | Each search iteration included the filters of the Date Range of 1990 to 2020 and only books was selected for the Content Type. The references had to be individually uploaded to Zotero and many of the yielded results were irrelevant to the study. In order to pull in actual articles or reports, the results were screened by title and description. Potentially eligible titles were saved to Zotero for later screening. Accordingly, the smaller number of references in this folder reflect this process and explains the difference from the actual yield.  Search Iterations:   1. (online OR “social media” OR internet OR Twitter OR Facebook OR 8Chan OR 8Kun OR Gab OR Telegram OR TikTok OR Reddit OR WhatsApp OR Instagram OR “social networking site*” OR cybervictimization OR “online incivility”) AND (“hate speech” OR cyberhate OR extrem* narrative* OR racis* OR radical* OR speech OR ideolog* OR islamophobi* OR homophobi* OR transphobi* OR misogyny OR disablism OR discrim* OR terror*) AND (interven* OR option* OR strategy* OR “counter narrative*” OR “nudge” OR “norm* intervention” OR “norm* nudge” OR counternarrative* OR “alternative narrative*” OR campaign* OR counter* OR peer-to-peer OR prevent* OR disrupt* OR stop* OR fight* OR redirect* OR censoring hate content”) AND (comparison* OR quantitative OR quasi- experiment* OR survey* OR interview* OR poll* OR mixed-methods OR individual-level OR group-level OR control* OR experiment* OR study OR studies OR evaluat* OR MTurk OR longitudinal OR random* OR “digital method*” OR “machine learning” OR “natural language processing” OR multisectoral OR review*) – yielded 12 results 2. (online OR “social media” OR internet OR Twitter OR Facebook OR 8Chan OR 8Kun OR Gab OR Telegram OR TikTok OR Reddit OR WhatsApp OR Instagram OR “social networking site*” OR cybervictimization OR “online incivility”) AND (“hate speech” OR cyberhate OR extrem* narrative* OR racis* OR radical* OR speech OR ideolog* OR islamophobi* OR homophobi* OR transphobi* OR misogyny OR disablism OR discrim* OR terror*) AND (interven* OR option* OR strategy* OR “counter narrative*” OR “nudge” OR “norm* intervention” OR “norm* nudge” OR counternarrative* OR “alternative narrative*” OR campaign* OR counter* OR peer-to-peer OR prevent* OR disrupt* OR stop* OR fight* OR redirect* OR censoring hate content”) – yielded 30 results 3. (online OR “social media” OR internet OR Twitter OR Facebook OR 8Chan OR 8Kun OR Gab OR Telegram OR TikTok OR Reddit OR WhatsApp OR Instagram OR “social networking site*” OR cybervictimization OR “online incivility”) AND (“hate speech” OR cyberhate OR extrem* narrative* OR racis* OR radical* OR speech OR ideolog* OR islamophobi* OR homophobi* OR transphobi* OR misogyny OR disablism OR discrim* OR terror*) – yielded 685 results 4. (online OR “social media” OR internet OR Twitter OR Facebook OR 8Chan OR 8Kun OR Gab OR Telegram OR TikTok OR Reddit OR WhatsApp OR Instagram OR “social networking site*” OR cybervictimization OR “online incivility”) – yielded 7,235 results 5. (online radical* OR online terror* OR online extrem* OR “hate speech” OR cyberhate OR “social media” OR internet OR Twitter OR Facebook OR 8Chan OR 8Kun OR Gab OR Telegram OR TikTok OR Reddit OR WhatsApp OR Instagram OR “social networking site*”) – yielded 1,507 results |
| Search date | 11 November 2020 |
| Initials | HJC |
| Database/Website/Journal/Strategy | SAGE Journals Online (<https://journals.sagepub.com/>) |
| Final Search String | Search field set to Anywhere: (online OR “social media” OR internet OR Twitter OR Facebook OR 8Chan OR 8Kun OR Gab OR Telegram OR TikTok OR Reddit OR WhatsApp OR Instagram OR “social networking site*” OR cybervictimization OR “online incivility”) AND (“hate speech” OR cyberhate OR extrem* narrative* OR racis* OR radical* OR speech OR ideolog* OR islamophobi* OR homophobi* OR transphobi* OR misogyny OR disablism OR discrim* OR terror*) AND (interven* OR option* OR strategy* OR “counter narrative*” OR “nudge” OR “norm* intervention” OR “norm* nudge” OR counternarrative* OR “alternative narrative*” OR campaign* OR counter* OR peer- to-peer OR prevent* OR disrupt* OR stop* OR fight* OR redirect* OR censoring hate content”) AND (comparison* OR quantitative OR quasi-experiment* OR survey* OR interview* OR poll* OR mixed- methods OR individual-level OR group-level OR control* OR experiment* OR study OR studies OR evaluat* OR MTurk OR longitudinal OR random* OR “digital method*” OR “machine learning” OR “natural language processing” OR multisectoral OR review*) |
| Reported Yield | 4,475 |
| Actual Yield | 500 |
| Notes | SAGE Journals Online included the following journals of interest for the project that we were able to simultaneously search:   - Sociology (Sage Full-Text Journal Collection)   The search field of “anywhere” includes search fields of title, author, keywords, and abstract.  Restrictions: 1990 – 2020   1. First search yield: 4602   Searched with 'anywhere' for field  (online OR “social media” OR internet OR Twitter OR Facebook OR 8Chan OR 8Kun OR Gab OR Telegram OR TikTok OR Reddit OR WhatsApp OR Instagram OR “social networking site*” OR cybervictimization OR “online incivility”) AND (“hate speech” OR cyberhate OR extrem* narrative* OR racis* OR radical* OR speech OR ideolog* OR islamophobi* OR homophobi* OR transphobi* OR misogyny OR disablism OR discrim* OR terror*) AND (interven* OR option* OR strategy* OR “counter narrative*” OR “nudge” OR “norm* intervention” OR “norm* nudge” OR counternarrative* OR “alternative narrative*” OR campaign* OR counter* OR peer- to-peer OR prevent* OR disrupt* OR stop* OR fight* OR redirect* OR censoring hate content”) AND  (comparison* OR quantitative OR quasi-experiment* OR survey* OR interview* OR poll* OR mixed- methods OR individual-level OR group-level OR control* OR experiment* OR study OR studies OR evaluat* OR MTurk OR longitudinal OR random* OR “digital method*” OR “machine learning” OR “natural language processing” OR multisectoral OR review*)   1. Fourth search yield: 165   Tried a fourth search by abstract using the following search string...  (online OR “social media” OR internet OR Twitter OR Facebook OR 8Chan OR 8Kun OR Gab OR Telegram OR TikTok OR Reddit OR WhatsApp OR Instagram OR “social networking site*” OR cybervictimization OR “online incivility”) AND (“hate speech” OR cyberhate OR extrem* narrative* OR racis* OR radical* OR speech OR ideolog* OR islamophobi* OR homophobi* OR transphobi* OR misogyny OR disablism OR discrim* OR terror*)   1. Fifth search yield: 4475   For the fifth and last search, the search field was set to the 'anywhere' field and within the 1990-2020 year range. This yielded a substantial 4475, so the first 500 references were imported after sorting by the default 'relevance'.  (online OR “social media” OR internet OR Twitter OR Facebook OR 8Chan OR 8Kun OR Gab OR Telegram OR TikTok OR Reddit OR WhatsApp OR Instagram OR “social networking site*” OR cybervictimization OR “online incivility”) AND (“hate speech” OR cyberhate OR extrem* narrative* OR racis* OR radical* OR speech OR ideolog* OR islamophobi* OR homophobi* OR transphobi* OR misogyny OR disablism OR discrim* OR terror*) AND (interven* OR option* OR strategy* OR “counter narrative*” OR “nudge” OR “norm* intervention” OR “norm* nudge” OR counternarrative* OR “alternative narrative*” OR campaign* OR counter* OR peer- to-peer OR prevent* OR disrupt* OR stop* OR fight* OR redirect* OR censoring hate content”) AND (comparison* OR quantitative OR quasi-experiment* OR survey* OR interview* OR poll* OR mixed- methods OR individual-level OR group-level OR control* OR experiment* OR study OR studies OR evaluat* OR MTurk OR longitudinal OR random* OR “digital method*” OR “machine learning” OR “natural language processing” OR multisectoral OR review*) |
| Search date | 2 - 4 November 2020; 10 November 2020; 12-13 November 2020 |
| Initials | EMJ |
| Database/Website/Journal/Strategy | Sage Knowledge ebook collection (<https://sk.sagepub.com>) |
| Final Search String | Search field set to Full Text: online AND hate AND speech |
| Reported Yield | 1,484 |
| Actual Yield | 39 |
| Notes | Each search iteration included the filters of the Date Range of 1990 to 2020 and only books was selected for the Content Type. The references had to be individually uploaded to Zotero and many of the yielded results were irrelevant to the study. In order to pull in actual articles or reports, the results were screened by title and description. Potentially eligible titles were saved to Zotero for later screening. Accordingly, the smaller number of references in this folder reflect this process and explains the difference from the actual yield.  Search Iterations:   1. (online OR “social media” OR internet OR Twitter OR Facebook OR 8Chan OR 8Kun OR Gab OR Telegram OR TikTok OR Reddit OR WhatsApp OR Instagram OR “social networking site*” OR cybervictimization OR “online incivility”) AND (“hate speech” OR cyberhate OR extrem* narrative* OR racis* OR radical* OR speech OR ideolog* OR islamophobi* OR homophobi* OR transphobi* OR misogyny OR disablism OR discrim* OR terror*) AND (interven* OR option* OR strategy* OR “counter narrative*” OR “nudge” OR “norm* intervention” OR “norm* nudge” OR counternarrative* OR “alternative narrative*” OR campaign* OR counter* OR peer-to-peer OR prevent* OR disrupt* OR stop* OR fight* OR redirect* OR censoring hate content”) AND (comparison* OR quantitative OR quasi- experiment* OR survey* OR interview* OR poll* OR mixed-methods OR individual-level OR group-level OR control* OR experiment* OR study OR studies OR evaluat* OR MTurk OR longitudinal OR random* OR “digital method*” OR “machine learning” OR “natural language processing” OR multisectoral OR review*) in fulltext – yielded 27 results 2. (online OR “social media” OR internet OR Twitter OR Facebook OR 8Chan OR 8Kun OR Gab OR Telegram OR TikTok OR Reddit OR WhatsApp OR Instagram OR “social networking site*” OR cybervictimization OR “online incivility”) AND (“hate speech” OR cyberhate OR extrem* narrative* OR racis* OR radical* OR speech OR ideolog* OR islamophobi* OR homophobi* OR transphobi* OR misogyny OR disablism OR discrim* OR terror*) AND (interven* OR option* OR strategy* OR “counter narrative*” OR “nudge” OR “norm* intervention” OR “norm* nudge” OR counternarrative* OR “alternative narrative*” OR campaign* OR counter* OR peer-to-peer OR prevent* OR disrupt* OR stop* OR fight* OR redirect* OR censoring hate content”) in fulltext – yielded 39 results 3. (online OR “social media” OR internet OR Twitter OR Facebook OR 8Chan OR 8Kun OR Gab OR Telegram OR TikTok OR Reddit OR WhatsApp OR Instagram OR “social networking site*” OR cybervictimization OR “online incivility”) AND (“hate speech” OR cyberhate OR extrem* narrative* OR racis* OR radical* OR speech OR ideolog* OR islamophobi* OR homophobi* OR transphobi* OR misogyny OR disablism OR discrim* OR terror*) in fulltext – yielded 46 results 4. (online OR “social media” OR internet OR Twitter OR Facebook OR 8Chan OR 8Kun OR Gab OR Telegram OR TikTok OR Reddit OR WhatsApp OR Instagram OR “social networking site*” OR cybervictimization OR “online incivility”) in fulltext – yielded 70 results 5. online AND hate AND speech in fulltext – yielded 1,443 results |
| Search date | 19 August 2020 |
| Initials | SMW |
| Database/Website/Journal/Strategy | ScienceDirect (<https://www.sciencedirect.com/>) |
| Final Search String | Search field set to find articles with these terms: hate AND media AND speech AND online |
| Reported Yield | 1,065 |
| Actual Yield | 700 |
| Notes | Retrieved first 700 references sorted by relevance. In the advanced search option, only one bar for a search string was permitted and the search only allowed up to 500 characters within the string so the first search iteration did not include evaluation terms. The search field of find articles with these terms searches all parts of the document (i.e. full text, abstract, title, etc.) for instances of the term(s).  Search iterations:   1. Located yield: 132,578   Search field set to find articles with these terms: (“online radical*” OR “online terror*” OR “online extrem*” OR “hate speech” OR cyberhate OR “social media” OR internet OR Twitter OR Facebook OR 8Chan OR 8Kun OR Gab OR Telegram OR TikTok OR Reddit OR WhatsApp OR Instagram OR “social networking site*”) AND (extrem* narrative* OR racis* OR radical* OR “dangerous speech” OR ideology OR islamophobia OR homophobia OR transphobia OR misogyny OR disablism) AND (interven* OR option* OR strategy* OR “counter narrative*” OR counternarrative* OR “alternative narrative*” OR campaign* OR counter* OR peer-to-peer OR prevent* OR disrupt* OR stop* OR fight* OR redirect*)   1. Located yield: 54,122   Search field set to find articles with these terms: (“online radical*” OR “online terror*” OR “online extrem*” OR “hate speech” OR cyberhate OR “social media” OR internet OR Twitter OR Facebook OR 8Chan OR 8Kun OR Gab OR Telegram OR TikTok OR Reddit OR WhatsApp OR Instagram OR “social networking site*”) AND (extrem* narrative* OR racis* OR radical* OR “dangerous speech” OR ideology OR islamophobia OR homophobia OR transphobia OR misogyny OR disablism)   1. Located yield: 14,782   Search field set to find articles with these terms: (“online radical*” OR “online terror*” OR “online extrem*” OR “hate speech” OR cyberhate OR “social media” OR internet OR Twitter OR Facebook OR 8Chan OR 8Kun OR Gab OR Telegram OR TikTok OR Reddit OR WhatsApp OR Instagram OR “social networking site*”)   1. Located yield: 1,065   Search field set to find articles with these terms: hate AND media AND speech AND online |
| Search date | 13 November 2020 |
| Initials | EMJ |
| Database/Website/Journal/Strategy | Scopus (<https://www.scopus.com/home.uri>) |
| Final Search String | Searched within article title, abstract, keywords: (online OR “social media” OR internet OR twitter OR facebook OR 8chan OR 8kun OR gab OR telegram OR tiktok OR reddit OR whatsapp OR instagram OR “social networking site*” OR cybervictimization OR “online incivility”) AND (“hate speech” OR cyberhate OR extrem* AND narrative* OR racis* OR radical* OR speech OR ideolog* OR islamophobi* OR homophobi* OR transphobi* OR misogyny OR disablism OR discrim* OR terror*) AND (interven* OR option* OR strategy* OR “counter narrative*” OR “nudge” OR “norm* intervention” OR “norm* nudge” OR counternarrative* OR “alternative narrative*” OR campaign* OR counter* OR peer-to-peer OR prevent* OR disrupt* OR stop* OR fight* OR redirect) AND (comparison* OR quantitative OR quasi-experiment* OR survey* OR interview* OR poll* OR mixed-methods OR individual-level OR group-level OR control* OR experiment* OR study OR studies OR evaluat* OR mturk OR longitudinal OR random* OR “review*”) |
| Reported Yield | 448 |
| Actual Yield | 448 |
| Notes | Due to a character limit for each search string, a few search terms had to be excluded from the third and fourth search string. Searched within article title, abstract, keywords, as a search in All fields came back with 43,045 hits. The publication year was restricted to 1990-2020. All yielded results were brought in. |
| Search date | 13 November 2020 |
| Initials | EMJ |
| Database/Website/Journal/Strategy | Social Science Research Network (SSRN) (<https://www.ssrn.com/index.cfm/en/>) |
| Final Search String | online hate speech |
| Reported Yield | 106 |
| Actual Yield | 106 |
| Notes | In the advanced search option, only one bar for a search string was permitted and the search only allowed up to 210 characters within the string. All yielded results were brought into Zotero.  Search iteration:   1. (online OR “social media” OR internet OR Twitter OR Facebook OR 8Chan OR 8Kun OR Gab OR Telegram OR TikTok OR Reddit OR WhatsApp OR Instagram OR “social networking site*” OR cybervictimization OR “online incivility”) AND (“hate speech” OR cyberhate) – 0 yielded results 2. (online OR “social media” OR internet) AND (“hate speech” or radical* or extrem*) AND (interven* OR “counter narrative*” OR strategy*) AND (comparison* OR study OR review*) – 0 yielded results 3. (online OR “social media” OR internet OR Twitter OR Facebook OR 8Chan OR 8Kun OR Gab OR Telegram OR TikTok OR Reddit OR WhatsApp OR Instagram OR “social networking site*” OR cybervictimization OR “online incivility”) – 0 yielded results 4. online hate speech – 106 yielded results |
| Search date | 13 November 2020 |
| Initials | EMJ |
| Database/Website/Journal/Strategy | Social Sciences Citation Index (SSCI) (<https://www-webofscience-com.libproxy.temple.edu/wos/woscc/basic-search>) |
| Final Search String | Search field set to all fields: (online OR “social media” OR internet OR Twitter OR Facebook OR 8Chan OR 8Kun OR Gab OR Telegram OR TikTok OR Reddit OR WhatsApp OR Instagram OR “social networking site*” OR cybervictimization OR “online incivility”) AND (“hate speech” OR cyberhate OR extrem* narrative* OR racis* OR radical* OR speech OR ideolog* OR islamophobi* OR homophobi* OR transphobi* OR misogyny OR disablism OR discrim* OR terror*) AND (interven* OR option* OR strategy* OR “counter narrative*” OR “nudge” OR “norm* intervention” OR “norm* nudge” OR counternarrative* OR “alternative narrative*” OR campaign* OR counter* OR peer-to-peer OR prevent* OR disrupt* OR stop* OR fight* OR redirect* OR “censoring hate content”) AND (comparison* OR quantitative OR quasi-experiment* OR survey* OR interview* OR poll* OR mixed-methods OR individual-level OR group-level OR control* OR experiment* OR study OR studies OR evaluat* OR MTurk OR longitudinal OR random* OR “digital method*” OR “machine learning” OR “natural language processing” OR multisectoral OR review*) |
| Reported Yield | 1,203 |
| Actual Yield | 1,203 |
| Notes | Year restriction: 1990 – 2020  Exported the results 500 at a time to Refworks and then uploaded to Zotero. All yielded results were brought into Zotero.  Search field of all fields includes fields such as topic, title, author, publication titles, abstract, keywords, and web of science categories. |
| Search date | 18 November 2020 |
| Initials | HN |
| Database/Website/Journal/Strategy | Sociological Abstracts (<https://about.proquest.com/en/products-services/socioabs-set-c/>) |
| Final Search String | Search field set to abstract: (online OR “social media” OR internet OR Twitter OR Facebook OR 8Chan OR 8Kun OR Gab OR Telegram OR TikTok OR Reddit OR WhatsApp OR Instagram OR “social networking site*” OR cybervictimization OR “online incivility”) AND (“hate speech” OR cyberhate OR extrem* narrative* OR racis* OR radical* OR speech OR ideolog* OR islamophobi* OR homophobi* OR transphobi* OR misogyny OR disablism OR discrim* OR terror*) AND (interven* OR option* OR strategy* OR “counter narrative*” OR “nudge” OR “norm* intervention” OR “norm* nudge” OR counternarrative* OR “alternative narrative*” OR campaign* OR counter* OR peer-to-peer OR prevent* OR disrupt* OR stop* OR fight* OR redirect* OR “censoring hate content”) |
| Reported Yield | 487 |
| Actual Yield | 484 |
| Notes | Search iterations:   1. (online OR “social media” OR internet OR Twitter OR Facebook OR 8Chan OR 8Kun OR Gab OR Telegram OR TikTok OR Reddit OR WhatsApp OR Instagram OR “social networking site*” OR cybervictimization OR “online incivility”) AND (“hate speech” OR cyberhate OR extrem* narrative* OR racis* OR radical* OR speech OR ideolog* OR islamophobi* OR homophobi* OR transphobi* OR misogyny OR disablism OR discrim* OR terror*) AND (interven* OR option* OR strategy* OR “counter narrative*” OR “nudge” OR “norm* intervention” OR “norm* nudge” OR counternarrative* OR “alternative narrative*” OR campaign* OR counter* OR peer-to-peer OR prevent* OR disrupt* OR stop* OR fight* OR redirect* OR “censoring hate content”) AND (comparison* OR quantitative OR quasi- experiment* OR survey* OR interview* OR poll* OR mixed-methods OR individual-level OR group-level OR control* OR experiment* OR study OR studies OR evaluat* OR MTurk OR longitudinal OR random* OR “digital method*” OR “machine learning” OR “natural language processing” OR multisectoral OR review*) Results: 41,307 2. Search field set to abstract: (online OR “social media” OR internet OR Twitter OR Facebook OR 8Chan OR 8Kun OR Gab OR Telegram OR TikTok OR Reddit OR WhatsApp OR Instagram OR “social networking site*” OR cybervictimization OR “online incivility”) AND (“hate speech” OR cyberhate OR extrem* narrative* OR racis* OR radical* OR speech OR ideolog* OR islamophobi* OR homophobi* OR transphobi* OR misogyny OR disablism OR discrim* OR terror*) AND (interven* OR option* OR strategy* OR “counter narrative*” OR “nudge” OR “norm* intervention” OR “norm* nudge” OR counternarrative* OR “alternative narrative*” OR campaign* OR counter* OR peer-to-peer OR prevent* OR disrupt* OR stop* OR fight* OR redirect* OR “censoring hate content”) AND (comparison* OR quantitative OR quasi-experiment* OR survey* OR interview* OR poll* OR mixed-methods OR individual-level OR group-level OR control* OR experiment* OR study OR studies OR evaluat* OR MTurk OR longitudinal OR random* OR “digital method*” OR “machine learning” OR “natural language processing” OR multisectoral OR review*) Results: 299 3. Search field set to abstract: (online OR “social media” OR internet OR Twitter OR Facebook OR 8Chan OR 8Kun OR Gab OR Telegram OR TikTok OR Reddit OR WhatsApp OR Instagram OR “social networking site*” OR cybervictimization OR “online incivility”) AND (“hate speech” OR cyberhate OR extrem* narrative* OR racis* OR radical* OR speech OR ideolog* OR islamophobi* OR homophobi* OR transphobi* OR misogyny OR disablism OR discrim* OR terror*) AND (interven* OR option* OR strategy* OR “counter narrative*” OR “nudge” OR “norm* intervention” OR “norm* nudge” OR counternarrative* OR “alternative narrative*” OR campaign* OR counter* OR peer-to-peer OR prevent* OR disrupt* OR stop* OR fight* OR redirect* OR “censoring hate content”) Results: 487 |
| Search date | 29 December 2020; 30 December 2020 |
| Initials | SW |
| Database/Website/Journal/Strategy | SpringerLink (<https://link.springer.com/>) |
| Final Search String | hate speech AND intervention AND online |
| Reported Yield | 2,322 |
| Actual Yield | 1,000 |
| Notes | Search iterations: in main search field which searches in multiple fields (however, the database does not indicate which search fields it uses to conduct the basic or advanced search)   1. Full string: (online OR “social media” OR internet OR Twitter OR Facebook OR 8Chan OR 8Kun OR Gab OR Telegram OR TikTok OR Reddit OR WhatsApp OR Instagram OR “social networking site*” OR cybervictimization OR “online incivility”) AND (“hate speech” OR cyberhate OR extrem* narrative* OR racis* OR radical* OR speech OR ideolog* OR islamophobi* OR homophobi* OR transphobi* OR misogyny OR disablism OR discrim* OR terror*) AND (interven* OR option* OR strategy* OR “counter narrative*” OR “nudge” OR “norm* intervention” OR “norm* nudge” OR counternarrative* OR “alternative narrative*” OR campaign* OR counter* OR peer-to-peer OR prevent* OR disrupt* OR stop* OR fight* OR redirect* OR “censoring hate content”) AND (comparison* OR quantitative OR quasi-experiment* OR survey* OR interview* OR poll* OR mixed-methods OR individual-level OR group-level OR control* OR experiment* OR study OR studies OR evaluat* OR MTurk OR longitudinal OR random* OR “digital method*” OR “machine learning” OR “natural language processing” OR multisectoral OR review*) - 38,911 hits 2. hate speech AND intervention AND online - 2,322 - not possible to import multiple references at the same time, had to click on each and download into Zotero. Sorted by relevancy, imported the first 1000. |
| Search date | 26 August 2020 |
| Initials | SMW |
| Database/Website/Journal/Strategy | Taylor & Francis Online (<https://www.tandfonline.com/>) |
| Final Search String | Search field set to “anywhere”: (hate AND speech AND “social media” AND intervention) |
| Reported Yield | 1,946 |
| Actual Yield | 1,919 |
| Notes | Year restriction: 1990 – 2020  Taylor & Francis included several journals of interest for the project that we were able to simultaneously search the following journals:   - Behavioral Sciences of Terrorism and Political Aggression - Critical Studies on Terrorism - Dynamics of Asymmetric Conflict - Intelligence and National Security - Studies in Conflict & Terrorism - Terrorism and Political Violence   Search iterations: The search function only allowed a limited number of characters and did not allow additional lines, each of the string tiers had to be searched separately. Used advance search - which was limited in the number of characters. Therefore, restricted to running the individual search strings below.  The search field of “anywhere” includes search fields of title, author, keywords, and abstract.   1. Located yield: 0   (“online radical*” OR “online terror*” OR “online extrem*” OR “hate speech” OR cyberhate OR “social media” OR internet OR Twitter OR Facebook OR 8Chan OR 8Kun OR Gab OR Telegram OR TikTok OR Reddit OR WhatsApp OR Instagram OR “social networking site*”) AND (extrem* narrative* OR racis* OR radical* OR “dangerous speech” OR ideology OR islamophobia OR homophobia OR transphobia OR misogyny OR disablism) AND (interven* OR option* OR strategy* OR “counter narrative*” OR counternarrative* OR “alternative narrative*” OR campaign* OR counter* OR peer-to-peer OR prevent* OR disrupt* OR stop* OR fight* OR redirect*)   1. Located yield: 0   (“online radical*” OR “online terror*” OR “online extrem*” OR “hate speech” OR cyberhate OR “social media” OR internet OR Twitter OR Facebook OR 8Chan OR 8Kun OR Gab OR Telegram OR TikTok OR Reddit OR WhatsApp OR Instagram OR “social networking site*”) AND (extrem* narrative* OR racis* OR radical* OR “dangerous speech” OR ideology OR islamophobia OR homophobia OR transphobia OR misogyny OR disablism)   1. Located yield: 3,456   (“online radical*” OR “online terror*” OR “online extrem*” OR “hate speech” OR cyberhate OR “social media” OR internet OR Twitter OR Facebook OR 8Chan OR 8Kun OR Gab OR Telegram OR TikTok OR Reddit OR WhatsApp OR Instagram OR “social networking site*”)   1. Located yield: 1,946   (hate AND speech AND “social media” AND intervention) |
| Search date | 19 November 2020 |
| Initials | HN |
| Database/Website/Journal/Strategy | Web of Science (<https://www-webofscience-com.libproxy.temple.edu/wos/woscc/basic-search>) |
| Final Search String | Search field set to all fields: (online OR “social media” OR internet OR Twitter OR Facebook OR 8Chan OR 8Kun OR Gab OR Telegram OR TikTok OR Reddit OR WhatsApp OR Instagram OR “social networking site*” OR cybervictimization OR “online incivility”) AND (“hate speech” OR cyberhate OR extrem* narrative* OR racis* OR radical* OR speech OR ideolog* OR islamophobi* OR homophobi* OR transphobi* OR misogyny OR disablism OR discrim* OR terror*) AND (interven* OR option* OR strategy* OR “counter narrative*” OR “nudge” OR “norm* intervention” OR “norm* nudge” OR counternarrative* OR “alternative narrative*” OR campaign* OR counter* OR peer-to-peer OR prevent* OR disrupt* OR stop* OR fight* OR redirect* OR “censoring hate content”) AND (comparison* OR quantitative OR quasi- experiment* OR survey* OR interview* OR poll* OR mixed-methods OR individual-level OR group-level OR control* OR experiment* OR study OR studies OR evaluat* OR MTurk OR longitudinal OR random* OR “digital method*” OR “machine learning” OR “natural language processing” OR multisectoral OR review*) |
| Reported Yield | 7,489 |
| Actual Yield | 468 |
| Notes | Retrieved first ~500 references sorted by relevance  Search field of all fields includes fields such as topic, title, author, publication titles, abstract, keywords, and web of science categories.  Search iterations:   1. Search field set to all fields: (online OR “social media” OR internet OR Twitter OR Facebook OR 8Chan OR 8Kun OR Gab OR Telegram OR TikTok OR Reddit OR WhatsApp OR Instagram OR “social networking site*” OR cybervictimization OR “online incivility”) AND (“hate speech” OR cyberhate OR extrem* narrative* OR racis* OR radical* OR speech OR ideolog* OR islamophobi* OR homophobi* OR transphobi* OR misogyny OR disablism OR discrim* OR terror*) AND (interven* OR option* OR strategy* OR “counter narrative*” OR “nudge” OR “norm* intervention” OR “norm* nudge” OR counternarrative* OR “alternative narrative*” OR campaign* OR counter* OR peer-to-peer OR prevent* OR disrupt* OR stop* OR fight* OR redirect* OR “censoring hate content”) AND (comparison* OR quantitative OR quasi- experiment* OR survey* OR interview* OR poll* OR mixed-methods OR individual-level OR group-level OR control* OR experiment* OR study OR studies OR evaluat* OR MTurk OR longitudinal OR random* OR “digital method*” OR “machine learning” OR “natural language processing” OR multisectoral OR review*) Results: 7,489 |
| Search date | 28 December 2020 |
| Initials | SMW |
| Database/Website/Journal/Strategy | Wiley Online Library (<https://onlinelibrary.wiley.com/>) |
| Final Search String | Search field set to Abstract: (hate and intervention and online) |
| Reported Yield | 616 |
| Actual Yield | 553 |
| Notes | Restrictions:   - January 1990 - December 2020 - English, German, Arabic, Persian - Searched within abstracts   Search iterations:   1. Located yield = 6,840,285   Search field set to Abstract: (“online radical*” OR “online terror*” OR “online extrem*” OR “hate speech” OR cyberhate OR “social media” OR internet OR Twitter OR Facebook OR 8Chan OR 8Kun OR Gab OR Telegram OR TikTok OR Reddit OR WhatsApp OR Instagram OR “social networking site*”) AND (extrem* narrative* OR racis* OR radical* OR “dangerous speech” OR ideology OR islamophobia OR homophobia OR transphobia OR misogyny OR disablism) AND (interven* OR option* OR strategy* OR “counter narrative*” OR counternarrative* OR “alternative narrative*” OR campaign* OR counter* OR peer-to-peer OR prevent* OR disrupt* OR stop* OR fight* OR redirect*) AND (comparison* OR quantitative OR quasi-experiment* OR survey* OR interview* OR poll* OR mixed-methods OR individual-level OR group-level OR control* OR experiment* OR study OR studies OR evaluat* OR MTurk OR longitudinal OR random* OR “digital method*” OR “machine learning” OR “natural language processing” OR multisectoral OR review*)   1. Located Yield = 2,435,649   Search field set to Abstract: (“online radical*” OR “online terror*” OR “online extrem*” OR “hate speech” OR cyberhate OR “social media” OR internet OR Twitter OR Facebook OR 8Chan OR 8Kun OR Gab OR Telegram OR TikTok OR Reddit OR WhatsApp OR Instagram OR “social networking site*”) AND (extrem* narrative* OR racis* OR radical* OR “dangerous speech” OR ideology OR islamophobia OR homophobia OR transphobia OR misogyny OR disablism) AND (interven* OR option* OR strategy* OR “counter narrative*” OR counternarrative* OR “alternative narrative*” OR campaign* OR counter* OR peer-to-peer OR prevent* OR disrupt* OR stop* OR fight* OR redirect*)   1. Located Yield = 235,913   Search field set to Abstract: (“online radical*” OR “online terror*” OR “online extrem*” OR “hate speech” OR cyberhate OR “social media” OR internet OR Twitter OR Facebook OR 8Chan OR 8Kun OR Gab OR Telegram OR TikTok OR Reddit OR WhatsApp OR Instagram OR “social networking site*”) AND (extrem* narrative* OR racis* OR radical* OR “dangerous speech” OR ideology OR islamophobia OR homophobia OR transphobia OR misogyny OR disablism)   1. Located Yield = 159,927   Search field set to Abstract: (“online radical*” OR “online terror*” OR “online extrem*” OR “hate speech” OR cyberhate OR “social media” OR internet)   1. Located Yield = 616   Search field set to Abstract: (“online radical*” OR cyberhate OR “social media” OR “dangerous speech”) |
| Search date | 9 November 2020 |
| Initials | AMO |
| Database/Website/Journal/Strategy | WorldCat & WorldCat Dissertations (<https://www.worldcat.org/>) |
| Final Search String | Search field set to “Keyword”: (online OR “social media” OR “social networking site” OR cybervictimization OR “online incivility”) AND (“hate speech” OR cyberhate OR “extremist narrative” OR racism OR radical OR speech OR ideology) AND (intervention OR option OR strategy OR “counter narrative” OR nudge OR norm intervention OR norm nudge OR counternarrative OR “alternative narrative” OR campaign OR counter OR peer-to-peer OR prevent OR disrupt OR stop OR fight OR redirect OR “censoring hate content”) AND (comparison OR quantitative OR qualitative OR quasi-experiment OR mixed-methods OR experiment) |
| Reported Yield | 1,115 |
| Actual Yield | 882 |
| Notes | Restrictions   - Year: 1989-2021 - Keyword search   Search iterations:   1. Search field set to “Keyword”: (online OR social media OR internet OR Twitter OR Facebook OR 8Chan OR 8Kun OR Gab OR Telegram OR TikTok OR Reddit OR WhatsApp OR Instagram OR social networking site OR cybervictimization OR online incivility) AND (hate speech OR cyberhate OR extremist narrative OR racism OR radical OR speech OR ideology OR islamophobia OR homophobia OR transphobia OR misogyny OR disablism OR discrimination OR terrorism) AND (intervention OR option OR strategy OR counter narrative OR nudge OR norm intervention OR norm nudge OR counternarrative OR alternative narrative OR campaign OR counter OR peer-to-peer OR prevent OR disrupt OR stop OR fight OR redirect OR censoring hate content) AND (comparison OR quantitative OR qualitative OR quasi-experiment OR mixed-methods OR experiment); yield: 2,167 2. Search field set to “Keyword”: (online OR “social media” OR social networking site OR cybervictimization OR online incivility) AND (hate speech OR cyberhate OR extremist narrative OR racism OR radical OR speech OR ideology OR islamophobia OR homophobia OR transphobia OR misogyny OR disablism OR discrimination OR terrorism) AND (intervention OR option OR strategy OR counter narrative OR nudge OR norm intervention OR norm nudge OR counternarrative OR alternative narrative OR campaign OR counter OR peer-to-peer OR prevent OR disrupt OR stop OR fight OR redirect OR censoring hate content) AND (comparison OR quantitative OR qualitative OR quasi-experiment OR mixed-methods OR experiment); yield: 1,646 3. Search field set to “Keyword”: (online OR “social media” OR “social networking site” OR cybervictimization OR “online incivility”) AND (“hate speech” OR cyberhate OR “extremist narrative” OR racism OR radical OR speech OR ideology) AND (intervention OR option OR strategy OR “counter narrative” OR nudge OR norm intervention OR norm nudge OR counternarrative OR “alternative narrative” OR campaign OR counter OR peer-to-peer OR prevent OR disrupt OR stop OR fight OR redirect OR “censoring hate content”) AND (comparison OR quantitative OR qualitative OR quasi-experiment OR mixed-methods OR experiment); yield: 1,115 |
| Search date | 21 August 2020 |
| Initials | AMO |
| Database/Website/Journal/Strategy | Annual Review of Criminology (<https://www.annualreviews.org/journal/criminol>) |
| Final Search String | n/a |
| Reported Yield | 66 |
| Actual Yield | 66 |
| Notes | Only four volumes exist. Imported all four volumes and did not perform a search. |
| Search date | 19 August 2020 |
| Initials | SMW |
| Database/Website/Journal/Strategy | Annual Review of Sociology (<https://www.annualreviews.org/journal/soc>) |
| Final Search String | (radicalization AND hate AND speech AND internet) |
| Reported Yield | 52 |
| Actual Yield | 52 |
| Notes | Very little available for project, even single word searches (i.e., radicalization, hate, intervention) came back sparse. |
| Search date | 5 September 2020 |
| Initials | SMW |
| Database/Website/Journal/Strategy | Journal for Deradicalization (<https://journals.sfu.ca/jd/index.php/jd>) |
| Final Search String | n/a |
| Reported Yield | 179 |
| Actual Yield | 178 |
| Notes | No search string was used as every article from the journal was imported. While some of the articles may appear to be written in German or another language, the abstracts have been translated. These articles were still imported for screening. |
| Search date | 2 September 2020 |
| Initials | EMJ |
| Database/Website/Journal/Strategy | Journal of Hate Studies (<https://jhs.press.gonzaga.edu/>) |
| Final Search String | n/a |
| Reported Yield | 156 |
| Actual Yield | 156 |
| Notes | There were only 156 articles across all 15 issues, all of the articles published within this journal were imported. |
| Search date | 19 November 2020 |
| Initials | HN |
| Database/Website/Journal/Strategy | Journal of Policing, Intelligence and Counter Terrorism (<https://www.tandfonline.com/toc/rpic20/current>) |
| Final Search String | Search field set to “anywhere”: (online OR “social media” OR internet OR Twitter OR Facebook OR 8Chan OR 8Kun OR Gab OR Telegram OR TikTok OR Reddit OR WhatsApp OR Instagram OR “social networking site*” OR cybervictimization OR “online incivility”) |
| Reported Yield | 126 |
| Actual Yield | 114 |
| Notes | Search iterations:   1. Search field set to “anywhere”: (online OR “social media” OR internet OR Twitter OR Facebook OR 8Chan OR 8Kun OR Gab OR Telegram OR TikTok OR Reddit OR WhatsApp OR Instagram OR “social networking site*” OR cybervictimization OR “online incivility”) AND (“hate speech” OR cyberhate OR extrem* narrative* OR racis* OR radical* OR speech OR ideolog* OR islamophobi* OR homophobi* OR transphobi* OR misogyny OR disablism OR discrim* OR terror*) AND (interven* OR option* OR strategy* OR “counter narrative*” OR “nudge” OR “norm* intervention” OR “norm* nudge” OR counternarrative* OR “alternative narrative*” OR campaign* OR counter* OR peer-to-peer OR prevent* OR disrupt* OR stop* OR fight* OR redirect* OR “censoring hate content”) AND (comparison* OR quantitative OR quasi- experiment* OR survey* OR interview* OR poll* OR mixed-methods OR individual-level OR group-level OR control* OR experiment* OR study OR studies OR evaluat* OR MTurk OR longitudinal OR random* OR “digital method*” OR “machine learning” OR “natural language processing” OR multisectoral OR review*) Results: 102 2. Search field set to “anywhere”: (online OR “social media” OR internet OR Twitter OR Facebook OR 8Chan OR 8Kun OR Gab OR Telegram OR TikTok OR Reddit OR WhatsApp OR Instagram OR “social networking site*” OR cybervictimization OR “online incivility”) AND (“hate speech” OR cyberhate OR extrem* narrative* OR racis* OR radical* OR speech OR ideolog* OR islamophobi* OR homophobi* OR transphobi* OR misogyny OR disablism OR discrim* OR terror*) AND (interven* OR option* OR strategy* OR “counter narrative*” OR “nudge” OR “norm* intervention” OR “norm* nudge” OR counternarrative* OR “alternative narrative*” OR campaign* OR counter* OR peer-to-peer OR prevent* OR disrupt* OR stop* OR fight* OR redirect* OR “censoring hate content”) Results: 102 3. Search field set to “anywhere”: (online OR “social media” OR internet OR Twitter OR Facebook OR 8Chan OR 8Kun OR Gab OR Telegram OR TikTok OR Reddit OR WhatsApp OR Instagram OR “social networking site*” OR cybervictimization OR “online incivility”) AND (“hate speech” OR cyberhate OR extrem* narrative* OR racis* OR radical* OR speech OR ideolog* OR islamophobi* OR homophobi* OR transphobi* OR misogyny OR disablism OR discrim* OR terror*) Results: 102 4. Search field set to “anywhere”: (online OR “social media” OR internet OR Twitter OR Facebook OR 8Chan OR 8Kun OR Gab OR Telegram OR TikTok OR Reddit OR WhatsApp OR Instagram OR “social networking site*” OR cybervictimization OR “online incivility”) Results: 126   Notes on contributors, book reviews, and editorials were filtered out, resulting in an actual yield of 114 resources to import |
| Search date | 15 December 2020 |
| Initials | SW |
| Database/Website/Journal/Strategy | Perspectives on Terrorism (<https://www.universiteitleiden.nl/perspectives-on-terrorism>) |
| Final Search String | (hate speech) AND pt:(Perspectives on Terrorism) |
| Reported Yield | 99 |
| Actual Yield | 99 |
| Notes | Search iterations:   1. Full search string: ((((online OR “social media” OR internet OR Twitter OR Facebook OR 8Chan OR 8Kun OR Gab OR Telegram OR TikTok OR Reddit OR WhatsApp OR Instagram OR “social networking site*” OR cybervictimization OR “online incivility”) AND (“hate speech” OR cyberhate OR extrem* narrative* OR racis* OR radical* OR speech OR ideolog* OR islamophobi* OR homophobi* OR transphobi* OR misogyny OR disablism OR discrim* OR terror*)) AND (interven* OR option* OR strategy* OR “counter narrative*” OR “nudge” OR “norm* intervention” OR “norm* nudge” OR counternarrative* OR “alternative narrative*” OR campaign* OR counter* OR peer-to-peer OR prevent* OR disrupt* OR stop* OR fight* OR redirect* OR “censoring hate content”)) AND (comparison* OR quantitative OR quasi-experiment* OR survey* OR interview* OR poll* OR mixed-methods OR individual-level OR group-level OR control* OR experiment* OR study OR studies OR evaluat* OR MTurk OR longitudinal OR random* OR “digital method*” OR “machine learning” OR “natural language processing” OR multisectoral OR review*)) - Error: Your keywords are generating a search query that is too long. The limit is 200 characters. Please try fewer or shorter keywords. 2. First three: (((online OR “social media” OR internet OR Twitter OR Facebook OR 8Chan OR 8Kun OR Gab OR Telegram OR TikTok OR Reddit OR WhatsApp OR Instagram OR “social networking site*” OR cybervictimization OR “online incivility”) AND (“hate speech” OR cyberhate OR extrem* narrative* OR racis* OR radical* OR speech OR ideolog* OR islamophobi* OR homophobi* OR transphobi* OR misogyny OR disablism OR discrim* OR terror*)) AND (interven* OR option* OR strategy* OR “counter narrative*” OR “nudge” OR “norm* intervention” OR “norm* nudge” OR counternarrative* OR “alternative narrative*” OR campaign* OR counter* OR peer-to-peer OR prevent* OR disrupt* OR stop* OR fight* OR redirect* OR “censoring hate content”)) - Error: Your keywords are generating a search query that is too long. The limit is 200 characters. Please try fewer or shorter keywords. 3. First two: ((online OR “social media” OR internet OR Twitter OR Facebook OR 8Chan OR 8Kun OR Gab OR Telegram OR TikTok OR Reddit OR WhatsApp OR Instagram OR “social networking site*” OR cybervictimization OR “online incivility”) AND (“hate speech” OR cyberhate OR extrem* narrative* OR racis* OR radical* OR speech OR ideolog* OR islamophobi* OR homophobi* OR transphobi* OR misogyny OR disablism OR discrim* OR terror*)) - Error: Your keywords are generating a search query that is too long. The limit is 200 characters. Please try fewer or shorter keywords. 4. First part: (online OR “social media” OR internet OR Twitter OR Facebook OR 8Chan OR 8Kun OR Gab OR Telegram OR TikTok OR Reddit OR WhatsApp OR Instagram OR “social networking site*” OR cybervictimization OR “online incivility”) - Error: Your keywords are generating a search query that is too long. The limit is 200 characters. Please try fewer or shorter keywords. 5. Simple one: ((online) AND (hate speech)) AND pt:(Perspectives on Terrorism) - 81 search results 6. (hate speech) AND pt:(Perspectives on Terrorism) – 99 search results |
| Search date | 19 November 2020; 22 – 23 November 2020 |
| Initials | AMO, SW, HJC |
| Database/Website/Journal/Strategy | Anti-Defamation League (ADL) Combating Hate – CYBERHATE (<https://www.adl.org/>) |
| Final Search String | n/a |
| Reported Yield | 79 |
| Actual Yield | 79 |
| Notes | Navigated to site reports via the following path: Research & Tools >>> Resource Library and then filtered by “type” and selected “reports.”  AMO: Two links were not for reports, but were a synopsis of events. Total number of reports imported: 10  SW: Included the last three reports.  HJC: Initially, ran a search on 17/11/20 but ran into trouble downloading and accessing the resource items due to being locked out of the website. At the next team meeting, the decision was made to collect all of the items in the “reports” category and divided the 75 items among the team members. 10 reports were imported by this researcher.  HJC (1/12/2020): All of the items were checked and the details were updated where required. All of the reports from the ADL website are now imported. |
| Search date | 25 November 2020 |
| Initials | HN |
| Database/Website/Journal/Strategy | Building Respect on the Internet by Combating Hate Speech (BRICkS) (<https://www.bricks-project.eu>) |
| Final Search String | n/a |
| Reported Yield | 9 |
| Actual Yield | 5 |
| Notes | Retrieved all relevant documents available on the website. |
| Search date | 27 November 2020 |
| Initials | HN |
| Database/Website/Journal/Strategy | Council of Europe (<https://www.coe.int/en/web/portal>) |
| Final Search String | (online OR “social media” OR internet OR Twitter OR Facebook OR 8Chan OR 8Kun OR Gab OR Telegram OR TikTok OR Reddit OR WhatsApp OR Instagram OR “social networking site*” OR cybervictimization OR “online incivility”) AND (“hate speech” OR cyberhate OR extrem* narrative* OR racis* OR radical* OR speech OR ideolog* OR islamophobi* OR homophobi* OR transphobi* OR misogyny OR disablism OR discrim* OR terror*) AND (interven* OR option* OR strategy* OR “counter narrative*” OR “nudge” OR “norm* intervention” OR “norm* nudge” OR counternarrative* OR “alternative narrative*” OR campaign* OR counter* OR peer-to-peer OR prevent* OR disrupt* OR stop* OR fight* OR redirect* OR “censoring hate content”) AND (comparison* OR quantitative OR quasi- experiment* OR survey* OR interview* OR poll* OR mixed-methods OR individual-level OR group-level OR control* OR experiment* OR study OR studies OR evaluat* OR MTurk OR longitudinal OR random* OR “digital method*” OR “machine learning” OR “natural language processing” OR multisectoral OR review*) |
| Reported Yield | 15,564 (Sorted by relevance and retrieved the first 500) |
| Actual Yield | 509 |
| Revised Yield | 188 |
| Notes | Search iterations:   1. (online OR “social media” OR internet OR Twitter OR Facebook OR 8Chan OR 8Kun OR Gab OR Telegram OR TikTok OR Reddit OR WhatsApp OR Instagram OR “social networking site*” OR cybervictimization OR “online incivility”) AND (“hate speech” OR cyberhate OR extrem* narrative* OR racis* OR radical* OR speech OR ideolog* OR islamophobi* OR homophobi* OR transphobi* OR misogyny OR disablism OR discrim* OR terror*) AND (interven* OR option* OR strategy* OR “counter narrative*” OR “nudge” OR “norm* intervention” OR “norm* nudge” OR counternarrative* OR “alternative narrative*” OR campaign* OR counter* OR peer-to-peer OR prevent* OR disrupt* OR stop* OR fight* OR redirect* OR “censoring hate content”) AND (comparison* OR quantitative OR quasi- experiment* OR survey* OR interview* OR poll* OR mixed-methods OR individual-level OR group-level OR control* OR experiment* OR study OR studies OR evaluat* OR MTurk OR longitudinal OR random* OR “digital method*” OR “machine learning” OR “natural language processing” OR multisectoral OR review*) Results: 15,564   SW notes: SMW and I went back and looked at each PDF and other formatted documents. Before creating parent items, we decided to screen out unrelated documents. |
| Search date | 31 December 2020 |
| Initials | SW |
| Database/Website/Journal/Strategy | Counter Narrative Handbook |
| Final Search String | n/a |
| Reported Yield | n/a |
| Actual Yield | n/a |
| Notes | Could not access website, but the handbook is an Institute for Strategic Dialogue publication. |
| Search date | 2 December 2020 |
| Initials | HN |
| Database/Website/Journal/Strategy | eMORE Project – Monitoring and Reporting Online Hate Speech in Europe: ECRI (<https://www.rissc.it/homepage/our-projects/emore-project/>) |
| Final Search String | n/a |
| Reported Yield | 7 |
| Actual Yield | 2 |
| Notes | Browsed the website and retrieved all related materials. |
| Search date | 15 September 2020 |
| Initials | HN |
| Database/Website/Journal/Strategy | European Commission against Racism and Intolerance (ECRI) – On combating hate Speech (<https://www.coe.int/en/web/european-commission-against-racism-and-intolerance/studies>) |
| Final Search String | n/a |
| Reported Yield | n/a |
| Actual Yield | n/a |
| Notes | This is a webpage that is a part of Council of Europe, and therefore, any relevant resources were gathered from our search of Council of Europe. |
| Search date | 18 – 19 November 2020 |
| Initials | EMJ |
| Database/Website/Journal/Strategy | Fundamental Rights Agency (<https://fra.europa.eu/en/about-fra>) |
| Final Search String | hate speech |
| Reported Yield | 30 |
| Actual Yield | 31 |
| Notes | Most of the results appeared to be irrelevant to the present study, especially when it was not restricted by product type. Restricted the search to the 4 product types of publications, easy-to-read reports, Fundamental Rights reports, and report/paper/summary.  Search iterations:   1. online AND (“hate speech” OR extrem* narrative* OR radical*)AND (interven* OR “counter narrative*”) AND (study OR review) – yielded 0 results 2. online AND “hate speech” AND interven* AND study – yielded 0 results 3. online AND “hate speech” AND interven* - yielded 0 results 4. online AND “hate speech” AND intervention – yielded 0 results 5. online “hate speech” intervention – yielded 0 results 6. online “hate speech” – yielded 1 result 7. hate speech – yielded 30 results |
| Search date | 25 November 2020 |
| Initials | HJC |
| Database/Website/Journal/Strategy | Hate Speech Watch (<http://archives.nohatespeechmovement.org/>) |
| Final Search String | n/a |
| Reported Yield | 42 |
| Actual Yield | 19 |
| Notes | The website was created as part of a “No Hate Speech Movement” campaign by the Council of Europe Youth Department. There seems to be little of value on the http://archives.nohatespeechmovement.org/ website itself. The only “reports” are just links to examples of hate speech online in the form of websites or social media which have been submitted by volunteers. However, when visiting the parent organization website https://www.coe.int/en/web/no-hate-campaign 42 publications by the organization could be located. There were a number of other resources which were not collected, including “action day” reports, “campaign tools”, and “educational materials”. Some of the links to both the publication and other types of resources were broken, so they couldn’t all be collected. Other reports were in languages outside the scope of this study such as Macedonian, Romanian, and Russian. |
| Search date | 24 August 2020 |
| Initials | AMO |
| Database/Website/Journal/Strategy | Home Office UK (<https://www.gov.uk/government/organisations/home-office>) |
| Final Search String | n/a |
| Reported Yield | 317 |
| Actual Yield | 1 |
| Notes | Did not perform a search, but navigated to research publications by navigating to: research and statistics >>> research. Screened each study by title and abstract and included only potentially relevant titles. |
| Search date | 15 December 2020 |
| Initials | SW |
| Database/Website/Journal/Strategy | Human Rights League (<https://www.hrl.sk/en>) |
| Final Search String | hate speech |
| Reported Yield | 7 |
| Actual Yield | 0 |
| Notes | No advanced search possible, so searched for hate speech and included none. |
| Search date | 1 December 2020 |
| Initials | HJC |
| Database/Website/Journal/Strategy | IN@CH – International Network Against Cyber Hate ([www.inach.net](http://www.inach.net)) |
| Final Search String | n/a |
| Reported Yield | 36 |
| Actual Yield | 34 |
| Notes | There was no search option, collected every item that was listed under “publications” on their website. |
| Search date | 15 December 2020 |
| Initials | SW |
| Database/Website/Journal/Strategy | INHOPE (<https://www.inhope.org/EN>) |
| Final Search String | n/a |
| Reported Yield | 0 |
| Actual Yield | 0 |
| Notes | Much of the articles on the site are focused on child sexual abuse and such content online. The only one that did seem relevant was not available in one of our languages. Screened each study by title and abstract and planned to include only potentially relevant titles. After an exhaustive search, it does not appear that there are any relevant scholarly articles available from this website. |
| Search date | 16 – 18 December 2020 |
| Initials | SW |
| Database/Website/Journal/Strategy | International Network for Hate Studies (INHS) online library (<https://internationalhatestudies.com/>) |
| Final Search String | n/a |
| Reported Yield | 547 |
| Actual Yield | 547 |
| Notes | Searched for “hate speech intervention” in the search box under online library with only one result which was imported. Then the online library was filtered by “hate speech” and everything was imported, which yielded 547 entries. |
| Search date | October 2020 |
| Initials | EMJ |
| Database/Website/Journal/Strategy | International Federation for Human Rights |
| Final Search String | n/a |
| Reported Yield | 0 |
| Actual Yield | 0 |
| Notes | Screened each study by title and abstract and included only potentially relevant titles. After an exhaustive search, it does not appear that there are any scholarly articles available from this website. |
| Search date | 1 December 2020 |
| Initials | HN |
| Database/Website/Journal/Strategy | International League Against Racism and Anti-Semitism (LICRA) (<https://www.inach.net/licra/>) |
| Final Search String | n/a |
| Reported Yield | n/a |
| Actual Yield | 73 |
| Notes | The original site is in French with no option to translate it to any other languages. Therefore, used Google Chrome as the browser as AMO recommended. There were no documents or reports on the websites. Therefore, only news was reviewed and retrieved the relevant ones.  SW note: These are not in a language we can screen, so we may need to just drop these. (31/12/2020)  SMW note: Moved items to trash as they did not fit into our language requirements (i.e., French). (1/1/2021) |
| Search date | 15 December 2020 |
| Initials | SMW |
| Database/Website/Journal/Strategy | Institute for the Student of Contemporary Antisemitism (iSCA) (<https://isca.indiana.edu/>) |
| Final Search String | (online OR internet OR hate speech OR cyberhate OR radical) |
| Reported Yield | n/a |
| Actual Yield | 20 |
| Notes | The website primarily offers courses and resources or antisemitism research. There were a couple of op-eds and short publications but very few were focuses on cyber or hate speech. There were a couple of webinars regarding social media and antisemitism but these didn’t appear relevant enough to include. Performed search using terms above but did not pull down retrieved documents. Screened each study by title and abstract and included only potentially relevant titles. |
| Search date | 16 December 2020 |
| Initials | SMW |
| Database/Website/Journal/Strategy | Irish Network Against Racism (<https://inar.ie/>) |
| Final Search String | (online OR internet OR hate speech OR cyberhate OR radical) |
| Reported Yield | n/a |
| Actual Yield | 3 |
| Notes | The website offered a lot of resources for reporting racism and hate crime... much less in terms of research and analyses. There were a couple of reports for “ireport” (an app for reporting racism, hate) but not much online material. The website contained a lot of webpages but most of the links and downloadable documents were out of date (most material only went back to 2018). Performed search using terms above but did not pull down retrieved documents. Screened each study by title and abstract and included only potentially relevant titles. |
| Search date | 3 December 2020; 6 December 2020 |
| Initials | SW |
| Database/Website/Journal/Strategy | The Institute for Strategic Dialogue (ISD) (<https://www.isdglobal.org/>) |
| Final Search String | No search string, all publications included |
| Reported Yield | 101 |
| Actual Yield | 100 |
| Notes | Located Yield: 96 + additional 5 items under “FREE Initiative: Full series of practical guides to respond to Far-Right threats”. Included all publications due to the importance of website for topic. |
| Search date | October 2020 |
| Initials | EMJ |
| Database/Website/Journal/Strategy | they can't – Fighting Antisemitism & Terrorism Online |
| Final Search String | n/a |
| Reported Yield | n/a |
| Actual Yield | n/a |
| Notes | This website no longer exists and therefore could not be searched. |
| Search date | 26 December 2020 |
| Initials | SMW |
| Database/Website/Journal/Strategy | Light on Project (<http://www.lighton-project.eu/site/main/page/home>) |
| Final Search String | n/a |
| Reported Yield | n/a |
| Actual Yield | n/a |
| Notes | Website is on longer active. Twitter was also searched and their account last posted in 2017. This organization doesn’t appear to still exist. |
| Search date | 20 November 2020 |
| Initials | EMJ |
| Database/Website/Journal/Strategy | MANDOLA – Monitoring and Detecting OnLine Hate Speech (<http://mandola-project.eu/>) |
| Final Search String | n/a |
| Reported Yield | 22 |
| Actual Yield | 22 |
| Notes | There were only 27 items on the publications page, so all of the deliverables were brought in, publications in journals and conferences, and working documents. There were 5 presentations, which were not brought in, which resulted in 22 references being recorded. |
| Search date | 16 December 2020 |
| Initials | SW |
| Database/Website/Journal/Strategy | Ministry of Justice, NZ (<https://www.justice.govt.nz/>) |
| Final Search String | n/a |
| Reported Yield | 37 |
| Actual Yield | 1 |
| Notes | Searched publications for “hate speech” with no results. Used the search box and included the 3 documents (2 were the same report) that included “hate speech”. Then navigated to: Home > Justice sector & policy > Research & data. Clicked on each tab and screened for studies by title and abstract. There were 35 evidence briefs with no potentially relevant titles. |
| Search date | 24 August 2020 |
| Initials | AMO |
| Database/Website/Journal/Strategy | Ministry of Justice, UK (<https://www.gov.uk/government/organisations/ministry-of-justice>) |
| Final Search String | n/a |
| Reported Yield | 233 |
| Actual Yield | 6 |
| Notes | Did not perform a search, but navigated to research publications by navigating to: research and statistics >>> research. Screened each study by title and abstract and included only potentially relevant titles. |
| Search date | 21 December 2020 |
| Initials | SW |
| Database/Website/Journal/Strategy | Department of Justice for each Australian state and territory (URLs below) |
| Final Search String | Hate/”hate speech” |
| Reported Yield | 32 |
| Actual Yield | 3 |
| Notes | Each state and territory has their own Department of Justice website. I went on each site and searched for relevant publications:   - ACT (<https://justice.act.gov.au/>) - hate or hate speech within the search box did not yield any results. There were no relevant publications when searching the “Justice Programs and Initiatives” tab. - NSW (<https://www.justice.nsw.gov.au/>) - hate in the search box yielded 20 results, which I scanned via the search function for relevancy, only 2 seemed relevant. - NT (<https://justice.nt.gov.au/>) - hate speech in the general search box had only 1 hit and it was a site related to reporting online extremism. Searching for just hate revealed a additional website on the topic of disability and was not relevant. - QLD (<https://www.justice.qld.gov.au/>) - searching for hate in the general search box yielded 4 hits, which were scanned for relevancy, but none were relevant. (“hate speech” yielded 0 results and hate speech without quotation marks 46 results with also including speech separately, so no relevancy. - SA (<https://www.agd.sa.gov.au/>) - hate speech in the general search box yielded one results which linked to the social media site. Hate by itself yielded 4 results, with three additional news sources. Nothing was relevant for import. - TAS (<https://www.justice.tas.gov.au/>) - no results for hate speech in search box, no results for hate in search box - VIC (<https://www.justice.vic.gov.au/>) - hate speech in general search box yielded one result, which linked to one report by the Human Rights Law Centre, which I included. Just hate as the search term, yielded an additional 2 results, none were relevant. - WA (<https://www.wa.gov.au/organisation/department-of-justice>) - no results for “hate speech”, without the quotation marks 42 results, but everything with speech is included. Just hate as a search term, yields 0 results. |
| Search date | 2 September 2020 |
| Initials | EMJ |
| Database/Website/Journal/Strategy | Online Antisemitism Taskforce (<https://www.antisemitismtaskforce.org/>) |
| Final Search String | n/a |
| Reported Yield | 0 |
| Actual Yield | 0 |
| Notes | After not finding any page or section with research on the website, I used google to search the website.   1. The search string “site:https://www.antisemitismtaskforce.org article” yielded no results. The search string “site:https://www.antisemitismtaskforce.org research” yielded no results. 2. The search string “site:https://www.antisemitismtaskforce.org “hate speech” yielded no results except for some blog posts. 3. The search string “site:https://www.antisemitismtaskforce.org hate speech” yielded no results except for some blog posts. 4. The search string “site:https://www.antisemitismtaskforce.org “online radical” yielded no results. The search string “site:https://www.antisemitismtaskforce.org “online extremism” yielded no results. 5. The search string “site:https://www.antisemitismtaskforce.org cyberhate” yielded no results. The search string “site:https://www.antisemitismtaskforce.org “extrem* narrative” yielded no results. 6. The search string “site:https://www.antisemitismtaskforce.org radical*” yielded no results. The search string “site:https://www.antisemitismtaskforce.org “counter narrative” yielded no results. 7. The search string “site:https://www.antisemitismtaskforce.org counter narrative” yielded no results. The search string “site:https://www.antisemitismtaskforce.org counter*” yielded no results. 8. After an exhaustive search, it does not appear that there are any scholarly articles available from this website. |
| Search date | 20 – 22 November 2020; 2 December 2020 |
| Initials | EMJ |
| Database/Website/Journal/Strategy | RAND (<https://www.rand.org/>) |
| Final Search String | “online radical” OR “online radicalization” OR “online terror” OR “online terrorism” OR “online extremism” OR “hate speech” OR cyberhate OR “social media” OR internet |
| Reported Yield | 396 |
| Actual Yield | 51 |
| Notes | Field: Any of these words  Limits: Year: 1/1/1990 - 12/31/2020  Advanced search, sorted by relevance, content type: research  Zotero could only save the search as a website. In order to pull in actual articles or reports, the results were screened by title and description. Potentially eligible titles were saved to Zotero for later screening.  Accordingly, the smaller number of references in this folder reflect this process and explains the difference from the actual yield.  Search iterations:   1. (online OR “social media” OR internet OR Twitter OR Facebook OR 8Chan OR 8Kun OR Gab OR Telegram OR TikTok OR Reddit OR WhatsApp OR Instagram OR “social networking site*” OR cybervictimization OR “online incivility”) AND (“hate speech” OR cyberhate OR extrem* narrative* OR racis* OR radical* OR speech OR ideolog* OR islamophobi* OR homophobi* OR transphobi* OR misogyny OR disablism OR discrim* OR terror*) AND (interven* OR option* OR strategy* OR “counter narrative*” OR “nudge” OR “norm* intervention” OR “norm* nudge” OR counternarrative* OR “alternative narrative*” OR campaign* OR counter* OR peer-to-peer OR prevent* OR disrupt* OR stop* OR fight* OR redirect* OR “censoring hate content”) AND (comparison* OR quantitative OR quasi-experiment* OR survey* OR interview* OR poll* OR mixed-methods OR individual-level OR group-level OR control* OR experiment* OR study OR studies OR evaluat* OR MTurk OR longitudinal OR random* OR “digital method*” OR “machine learning” OR “natural language processing” OR multisectoral OR review*) – within any of these words - yielded error page 2. (online OR “social media” OR internet OR Twitter OR Facebook OR 8Chan OR 8Kun OR Gab OR Telegram OR TikTok OR Reddit OR WhatsApp OR Instagram OR “social networking site*” OR cybervictimization OR “online incivility”) – within any of these words - yielded error page 3. (“hate speech” OR cyberhate OR extrem* narrative* OR racis* OR radical* OR speech OR ideolog* OR islamophobi* OR homophobi* OR transphobi* OR misogyny OR disablism OR discrim* OR terror*) – within any of these words – yielded error page 4. “hate speech” online – within all of these words – yielded 0 results 5. hate speech online – within all of these words – yielded 0 results 6. hate speech online – within all of these words – yielded 0 results 7. in all of these words: online AND in any of these words: hate OR terror OR extremism OR radical – filtered by content type research and 1/1990 - 12/2020 – yielded 200 results 8. “online radical” OR “online radicalization” OR “online terror” OR “online terrorism” OR “online extremism” OR “hate speech” OR cyberhate OR “social media” OR internet – filtered by content type research and 1/1990 - 12/2020 – yielded 396 results |
| Search date | 20 August 2020 |
| Initials | AMO |
| Database/Website/Journal/Strategy | RAND Europe (<https://www.rand.org/randeurope.html>) |
| Final Search String | n/a |
| Reported Yield | n/a |
| Actual Yield | 2 |
| Notes | Reviewed and screened publications under the following research topics: Defense and Security  Drugs, policing, and criminal justice Education policy, Science, technology, and innovation.  Did not complete a search of site as the search field is associated with the overall RAND website and not the sub website/page of RAND Europe. |
| Search date | 15 December 2020 |
| Initials | SMW |
| Database/Website/Journal/Strategy | Stand Up to Hate (<https://www.ed.ac.uk/literatures-languages-cultures/alwaleed/outreach-and-projects/stand-up>) |
| Final Search String | (hate speech AND online AND campaign) |
| Reported Yield | n/a |
| Actual Yield | 0 |
| Notes | Website is mostly blogs and news stories of hate crime instances in the UK. Any online mentions involves news events pertaining to gamers, Facebook initiatives Performed search using terms above but did not pull down retrieved documents. Screened each study by title and abstract and included only potentially relevant titles. After an exhaustive search, it does not appear that there are any scholarly articles available from this website. |
| Search date | 2 – 3 December 2020 |
| Initials | EMJ |
| Database/Website/Journal/Strategy | The Alan Turing Institute Online Hate Research Hub (<https://www.turing.ac.uk/research/research-programmes/public-policy/online-hate-research-hub>) |
| Final Search String | n/a |
| Reported Yield | 69 |
| Actual Yield | 69 |
| Notes | Under the Online Hate Research Hub, most of the resources were not publishable documents. The reports they listed under “Evidence on prevalence of online hate” were all downloaded, as were all of the reports/papers from the “Reading List for Online Hate and Abuse Research from the Alan Turing Institute.” |
| Search date | 16 December 2020 |
| Initials | SMW |
| Database/Website/Journal/Strategy | The Online Hate Prevention Institute (<https://ohpi.org.au/>) |
| Final Search String | (online AND campaign AND hate AND intervention) |
| Reported Yield | 315 |
| Actual Yield | 6 |
| Notes | OHPI offers news briefing related to online hate and racism... very little research. Performed search using terms above but did not pull down retrieved documents. Screened each study by title and abstract and included only potentially relevant titles. |
| Search date | October 2020 |
| Initials | EMJ |
| Database/Website/Journal/Strategy | Together against Hate on the Net |
| Final Search String | n/a |
| Reported Yield | n/a |
| Actual Yield | n/a |
| Notes | This website no longer exists and therefore could not be searched. |
| Search date | 21 December 2020 |
| Initials | SW |
| Database/Website/Journal/Strategy | UNESCO – Countering Online Hate Speech (<https://en.unesco.org/>) |
| Final Search String | (online OR “social media” OR internet OR Twitter OR Facebook OR 8Chan OR 8Kun OR Gab OR Telegram OR TikTok OR Reddit OR WhatsApp OR Instagram OR “social networking site*” OR cybervictimization OR “online incivility”) AND (“hate speech” OR cyberhate OR extrem* narrative* OR racis* OR radical* OR speech OR ideolog* OR islamophobi* OR homophobi* OR transphobi* OR misogyny OR disablism OR discrim* OR terror*) AND (interven* OR option* OR strategy* OR “counter narrative*” OR “nudge” OR “norm* intervention” OR “norm* nudge” OR counternarrative* OR “alternative narrative*” OR campaign* OR counter* OR peer-to-peer OR prevent* OR disrupt* OR stop* OR fight* OR redirect* OR “censoring hate content”) |
| Reported Yield | 517, when clicking through down to 28 |
| Actual Yield | 14 |
| Notes | Search iterations:   1. “hate speech” in search box yielded 27,500 results 2. (online OR “social media” OR internet OR Twitter OR Facebook OR 8Chan OR 8Kun OR Gab OR Telegram OR TikTok OR Reddit OR WhatsApp OR Instagram OR “social networking site*” OR cybervictimization OR “online incivility”) AND (“hate speech” OR cyberhate OR extrem* narrative* OR racis* OR radical* OR speech OR ideolog* OR islamophobi* OR homophobi* OR transphobi* OR misogyny OR disablism OR discrim* OR terror*) AND (interven* OR option* OR strategy* OR “counter narrative*” OR “nudge” OR “norm* intervention” OR “norm* nudge” OR counternarrative* OR “alternative narrative*” OR campaign* OR counter* OR peer-to-peer OR prevent* OR disrupt* OR stop* OR fight* OR redirect* OR “censoring hate content”) AND (comparison* OR quantitative OR quasi-experiment* OR survey* OR interview* OR poll* OR mixed-methods OR individual-level OR group-level OR control* OR experiment* OR study OR studies OR evaluat* OR MTurk OR longitudinal OR random* OR “digital method*” OR “machine learning” OR “natural language processing” OR multisectoral OR review*) - 226 results 3. (online OR “social media” OR internet OR Twitter OR Facebook OR 8Chan OR 8Kun OR Gab OR Telegram OR TikTok OR Reddit OR WhatsApp OR Instagram OR “social networking site*” OR cybervictimization OR “online incivility”) AND (“hate speech” OR cyberhate OR extrem* narrative* OR racis* OR radical* OR speech OR ideolog* OR islamophobi* OR homophobi* OR transphobi* OR misogyny OR disablism OR discrim* OR terror*) AND (interven* OR option* OR strategy* OR “counter narrative*” OR “nudge” OR “norm* intervention” OR “norm* nudge” OR counternarrative* OR “alternative narrative*” OR campaign* OR counter* OR peer-to-peer OR prevent* OR disrupt* OR stop* OR fight* OR redirect* OR “censoring hate content”) - 517 results. However, the number changes every time the page is changed or the search is redone. Any sources that were not a publication/report or were not in any of our search languages were not included. When clicking through the pages, it goes down to 28 hits. |
| Search date | 21 December 2020 |
| Initials | SW |
| Database/Website/Journal/Strategy | United Nations (<https://www.un.org/en/>) |
| Final Search String | “hate speech” AND “online intervention” |
| Reported Yield | 269 |
| Actual Yield | 23 |
| Notes | Entered “hate speech” AND “online intervention” into search box, yielding 269. A lot of sources were websites or news items. Scanned all to look for relevant reports or publications and added these. Many links however were no longer working. The following specific source we set out to incorporate was included: UN Committee on the Elimination of Racial Discrimination (CERD), General recommendation No. 35 : Combating racist hate speech, 26 September 2013, CERD/C/GC/35, available at: https://www.refworld.org/docid/53f457db4.html [accessed 22 December 2020] |
| Search date | 3 December 2020 |
| Initials | EMJ |
| Database/Website/Journal/Strategy | Urban Institute (<https://www.urban.org/>) |
| Final Search String | n/a |
| Reported Yield | 0 |
| Actual Yield | 0 |
| Notes | Screened each study by title and abstract and included only potentially relevant titles. After an exhaustive search, it does not appear that there are any scholarly articles available from this website.  Search iterations:   1. search all publications: (online OR “social media” OR internet OR Twitter OR Facebook OR 8Chan OR 8Kun OR Gab OR Telegram OR TikTok OR Reddit OR WhatsApp) - yielded 0 results 2. search all publications: hate speech - yielded 0 results 3. search all publications: hate - yielded 0 results 4. search all publications: online - yielded 5 results none of which were relevant 5. search all publications: internet - yielded 1 result which was not relevant 6. search all publications: social media - yielded 4 results none of which were relevant 7. search all publications: counter - yielded 6 results none of which were relevant   Switched search to google   1. site:https://www.urban.org/ “hate speech” – yielded 3 results none of which were relevant 2. site:https://www.urban.org/ hate speech – yielded 39 results none of which were relevant 3. site:https://www.urban.org/ counter – yielded 981 results - none of the results on the first few pages were relevant or publications 4. site:https://www.urban.org/ online – yielded 981 results - none of the results on the first few pages were relevant 5. site:https://www.urban.org/ online hate – yielded 137 results - none of the results on the first few pages were relevant |
| Search date | 26 August 2020; 09 September 2020; 14 September 2020; 25 October 2020; 28 - 29 October 2020; 11 November 2020; 13 – 19 November 2020 |
| Initials | SW |
| Database/Website/Journal/Strategy | VOX-Pol (<https://www.voxpol.eu/library/>) |
| Final Search String | n/a (import everything via website) |
| Reported Yield | 1,197 |
| Actual Yield | 1,124 |
| Notes | The online library had 1,148 Library Entries without entering any search terms on 26/08/20. Entering the search term string via abstract keywords and via tags yielded no results. Adding only one term: “online radical” into “search library” did work: 426 hits. Doesn’t import the references via the icon, may need to do this manually. Recommend importing all library entries, instead of a key word search, and then screen out the abstracts. The team decided to import all library entries given the relevance of the website. On 19/11/20, there were 1,197 references. |
| Search date | 27 December 2020 |
| Initials | SMW |
| Database/Website/Journal/Strategy | YouTube Creators for Change (<https://blog.youtube/news-and-events/introducing-youtube-creators-for-change>) |
| Final Search String | (hate AND hate speech) |
| Reported Yield | 0 |
| Actual Yield | 0 |
| Notes | YouTube creators for change did not have a “research” or “publication” section, therefore their blog was searched.  The blog consisted of news posts updating users to the new policies and collaborations implemented on YouTube to screen hateful content. Several resources for creators to check and make sure their work did not violate hate speech policies were found. An example was included in the folder. |

**Additional Search – March 2022**

Note. During the peer review process, we were asked to undertake an additional search of certain databases and to screen a larger number of references than we had done within our first search of these databases. This Appendix lays out the actions we took to adhere to this request of an additional search and includes notes on (1) the additional electronic searches, (2) additional systematic search notes, (3) an additional flowchart, and (4) additional information about DistillerSR’s AI stopping rule.

1. **Additional Electronic searches**

**Academic databases**

EBSCOHost platforms

*Search 2 (March 2022)*

Academic Search Complete

Communication Abstracts

Communication and Mass Media Complete

Criminal Justice Abstracts

Military and Government Collection

Psychology and Behavioural Sciences Collection

ProQuest platform

*Search 2 (March 2022)*

Applied Social Sciences Index & Abstracts (ASSIA)

Criminal Justice Database

Gender Watch

International Bibliography of the Social Sciences (IBSS)

National Criminal Justice Reference Service (NCJRS)

Policy File Index

ProQuest Dissertations & Theses Global

Political Science Database

Social Science Database

Sociological Abstracts

Sociology Database

Public Affairs Information Service (PAIS)

Worldwide Political Science Abstracts

Databases – Individually searched

*Search 2 (March 2022)*

Journals@Ovid (PsycINFO; PsycArticles)

Web of Science (BKCI-SSH; ESCI; CPCI-SSH; SSCI)

1. **Additional Systematic Search Notes**

| **Search 2 – March 2022** | |
| --- | --- |
| **Field** | **Description** |
| Search date | 17 – 24 March 2022 |
| Initials | EE, AMO, SMW, EMJ, SW |
| Database/Website/Journal/Strategy | EBSCOHost Research Databases (<https://www.ebsco.com/products/research-databases>) |
| Final Search String | Search 1 AND Search 2 AND Search 3 AND Search 4 |
| Reported Yield | 2429 |
| Actual Yield | 2429 |
| Notes | The search was limited to publication dates between 1 January 1990 and 31 December 2020. The following four searches were completed and then combined using the AND clause for the final search string:   1. TI (online OR "social media" OR internet OR Twitter OR Facebook OR 8chan OR 8Kun OR Gab OR Telegram OR TikTok OR Reddit OR WhatsApp OR Instagram OR "social networking site*" OR cybervictimization OR "online incivility") OR AB (online OR "social media" OR internet OR Twitter OR Facebook OR 8chan OR 8Kun OR Gab OR Telegram OR TikTok OR Reddit OR WhatsApp OR Instagram OR "social networking site*" OR cybervictimization OR "online incivility") OR KW (online OR "social media" OR internet OR Twitter OR Facebook OR 8chan OR 8Kun OR Gab OR Telegram OR TikTok OR Reddit OR WhatsApp OR Instagram OR "social networking site*" OR cybervictimization OR "online incivility") OR SU (online OR "social media" OR internet OR Twitter OR Facebook OR 8chan OR 8Kun OR Gab OR Telegram OR TikTok OR Reddit OR WhatsApp OR Instagram OR "social networking site*" OR cybervictimization OR "online incivility") 2. TI ("hate speech" OR cyberhate OR extrem* OR narrative OR racis* OR radical* OR speech OR ideolog* OR islamophobi* OR homophobi* OR transphobi* OR misogyny OR disablism OR discrim* OR terror*) OR AB ( "hate speech" OR cyberhate OR extrem* OR narrative OR racis* OR radical* OR speech OR ideolog* OR islamophobi* OR homophobi* OR transphobi* OR misogyny OR disablism OR discrim* OR terror*) OR KW ("hate speech" OR cyberhate OR extrem* OR narrative OR racis* OR radical* OR speech OR ideolog* OR islamophobi* OR homophobi* OR transphobi* OR misogyny OR disablism OR discrim* OR terror*) OR SU ("hate speech" OR cyberhate OR extrem* OR narrative OR racis* OR radical* OR speech OR ideolog* OR islamophobi* OR homophobi* OR transphobi* OR misogyny OR disablism OR discrim* OR terror*) 3. TI (interven* OR option* OR strategy* OR "counter narrative*" OR nudge* OR counternarrative* OR "alternative narrative*" OR campaign* OR counter*OR peer-to-peer OR prevent* OR disrupt* OR stop* OR fight* OR redirect* OR "censoring hate content") OR AB (interven* OR option* OR strategy* OR "counter narrative*" OR nudge* OR counternarrative* OR "alternative narrative*" OR campaign* OR counter*OR peer-to-peer OR prevent* OR disrupt* OR stop* OR fight* OR redirect* OR "censoring hate content") OR KW (interven* OR option* OR strategy* OR "counter narrative*" OR nudge* OR counternarrative* OR "alternative narrative*" OR campaign* OR counter*OR peer-to-peer OR prevent* OR disrupt* OR stop* OR fight* OR redirect* OR "censoring hate content") OR SU (interven* OR option* OR strategy* OR "counter narrative*" OR nudge* OR counternarrative* OR "alternative narrative*" OR campaign* OR counter*OR peer-to-peer OR prevent* OR disrupt* OR stop* OR fight* OR redirect* OR "censoring hate content") 4. TI (comparison* OR quantitative OR quasi-experiment* OR survey* OR interview* OR poll* OR mixed-methods OR individual-level OR group-level OR control* OR experiment* OR study OR studies OR evaluat* OR MTurk OR longitudinal OR random* OR "digital method*" OR "machine learning" OR "natural language processing" OR multisectoral OR review*) OR AB (comparison* OR quantitative OR quasi-experiment* OR survey* OR interview* OR poll* OR mixed-methods OR individual-level OR group-level OR control* OR experiment* OR study OR studies OR evaluat* OR MTurk OR longitudinal OR random* OR "digital method*" OR "machine learning" OR "natural language processing" OR multisectoral OR review*) OR KW (comparison* OR quantitative OR quasi-experiment* OR survey* OR interview* OR poll* OR mixed-methods OR individual-level OR group-level OR control* OR experiment* OR study OR studies OR evaluat* OR MTurk OR longitudinal OR random* OR "digital method*" OR "machine learning" OR "natural language processing" OR multisectoral OR review*) OR SU (comparison* OR quantitative OR quasi-experiment* OR survey* OR interview* OR poll* OR mixed-methods OR individual-level OR group-level OR control* OR experiment* OR study OR studies OR evaluat* OR MTurk OR longitudinal OR random* OR "digital method*" OR "machine learning" OR "natural language processing" OR multisectoral OR review*) |
| Search date | 17 – 24 March 2022 |
| Initials | EE, AMO, SMW, EMJ, SW |
| Database/Website/Journal/Strategy | ProQuest (<https://www.proquest.com/>) |
| Final Search String | (Search 1 AND Search 2 AND Search 3 AND Search 4) NOT (at.exact(“News” OR “General Information” OR “Obituary”) NOT stype.exact(“Newspapers” OR “Wire Feeds”)) |
| Reported Yield | 9297 |
| Actual Yield | 9297 |
| Notes | The search was limited to publication dates between 1 January 1990 and 31 December 2020. The following four searches were completed and then combined using the AND clause for the final search string:   1. ti(online OR "social media" OR internet OR Twitter OR Facebook OR 8chan OR 8Kun OR Gab OR Telegram OR TikTok OR Reddit OR WhatsApp OR Instagram OR "social networking site*" OR "cybervictimization" OR "online incivility") OR ab(online OR "social media" OR internet OR Twitter OR Facebook OR 8chan OR 8Kun OR Gab OR Telegram OR TikTok OR Reddit OR WhatsApp OR Instagram OR "social networking site*" OR "cybervictimization" OR "online incivility") OR mainsubject(online OR "social media" OR internet OR Twitter OR Facebook OR 8chan OR 8Kun OR Gab OR Telegram OR TikTok OR Reddit OR WhatsApp OR Instagram OR "social networking site*" OR "cybervictimization" OR "online incivility") 2. ti("hate speech" OR cyberhate OR extrem* OR narrative OR racis* OR radical* OR speech OR ideolog* OR islamophobi* OR homophobi* OR transphobi* OR misogyny OR disablism OR discrim* OR terror*) OR ab("hate speech" OR cyberhate OR extrem* OR narrative OR racis* OR radical* OR speech OR ideolog* OR islamophobi* OR homophobi* OR transphobi* OR misogyny OR disablism OR discrim* OR terror*) OR mainsubject("hate speech" OR cyberhate OR extrem* OR narrative OR racis* OR radical* OR speech OR ideolog* OR islamophobi* OR homophobi* OR transphobi* OR misogyny OR disablism OR discrim* OR terror*) 3. ti(interven* OR option* OR strategy* OR "counter narrative*" OR nudge* OR counternarrative* OR "alternative narrative*" OR campaign* OR counter*OR peer-to-peer OR prevent* OR disrupt* OR stop* OR fight* OR redirect* OR "censoring hate content") OR ab(interven* OR option* OR strategy* OR "counter narrative*" OR nudge* OR counternarrative* OR "alternative narrative*" OR campaign* OR counter*OR peer-to-peer OR prevent* OR disrupt* OR stop* OR fight* OR redirect* OR "censoring hate content") OR mainsubject(interven* OR option* OR strategy* OR "counter narrative*" OR nudge* OR counternarrative* OR "alternative narrative*" OR campaign* OR counter*OR peer-to-peer OR prevent* OR disrupt* OR stop* OR fight* OR redirect* OR "censoring hate content") 4. ti(comparison* OR quantitative OR quasi-experiment* OR survey* OR interview* OR poll* OR mixed-methods OR individual-level OR group-level OR control* OR experiment* OR study OR studies OR evaluat* OR MTurk OR longitudinal OR random* OR "digital method*" OR "machine learning" OR "natural language processing" OR multisectoral OR review*) OR ab(comparison* OR quantitative OR quasi-experiment* OR survey* OR interview* OR poll* OR mixed-methods OR individual-level OR group-level OR control* OR experiment* OR study OR studies OR evaluat* OR MTurk OR longitudinal OR random* OR "digital method*" OR "machine learning" OR "natural language processing" OR multisectoral OR review*) OR mainsubject(comparison* OR quantitative OR quasi-experiment* OR survey* OR interview* OR poll* OR mixed-methods OR individual-level OR group-level OR control* OR experiment* OR study OR studies OR evaluat* OR MTurk OR longitudinal OR random* OR "digital method*" OR "machine learning" OR "natural language processing" OR multisectoral OR review*) |
| Search date | 17 – 24 March 2022 |
| Initials | EE |
| Database/Website/Journal/Strategy | Web of Science (<https://www-webofscience-com.libproxy.temple.edu/wos/woscc/basic-search>) |
| Final Search String | Search 1 AND Search 2 AND Search 3 AND Search 4 |
| Reported Yield | 4145 |
| Actual Yield | 4145 |
| Notes | The search was limited to publication dates between 1 January 1990 and 31 December 2020. The following four searches were completed and then combined using the AND clause for the final search string:   1. TS = (online OR "social media" OR internet OR Twitter OR Facebook OR 8chan OR 8Kun OR Gab OR Telegram OR TikTok OR Reddit OR WhatsApp OR Instagram OR "social networking site*" OR "cybervictimization" OR "online incivility") 2. TS = ("hate speech" OR cyberhate OR extrem* OR narrative OR racis* OR radical* OR speech OR ideolog* OR islamophobi* OR homophobi* OR transphobi* OR misogyny OR disablism OR discrim* OR terror*) 3. TS = (interven* OR option* OR strategy* OR "counter narrative*" OR "nudge" OR "alternative narrative*" OR campaign* OR counter* OR peer-to-peer OR prevent* OR disrupt* OR stop* OR fight* OR redirect* OR "censoring hate") 4. TS = (comparison* OR quantitative OR quasi-experiment* OR survey* OR interview* OR poll* OR mixed-methods OR individual-level OR group-level OR control* OR experiment* OR study OR studies OR evaluat* OR MTurk OR longitudinal OR random* OR "digital method*" OR "machine learning" OR "natural language processing" OR multisectoral OR review*) |
| Search date | 17 – 24 March 2022 |
| Initials | EE |
| Database/Website/Journal/Strategy | Journals@Ovid (<https://ovidsp-dc2-ovid-com.libproxy.temple.edu/>) |
| Final Search String | Search 1 AND Search 2 AND Search 3 AND Search 4 |
| Reported Yield | 2112 |
| Actual Yield | 2112 |
| Notes | The search was limited to publication dates between 1990 and 2020 and 7 duplicates were removed from the search. The following four searches were completed and then combined using the AND clause for the final search string:   1. (online OR “social media” OR internet OR Twitter OR Facebook OR 8chan OR 8Kun OR Gab OR Telegram OR TikTok OR Reddit OR WhatsApp OR Instagram OR “social networking site*” OR “cybervictimization” OR “online incivility”) using search fields of AB, HW, ID, and TI 2. (“hate speech” OR cyberhate OR extrem* OR narrative OR racis* OR radical* OR speech OR ideolog* OR islamophobia* OR homophobi* OR transphobi* OR misogyny OR disablism OR discrim* OR terror*) using search fields of AB, HW, ID, and TI 3. (interven* OR option* OR strategy* OR “counter narrative*” OR nudge* OR counternarrative* OR “alternative narrative*” OR campaign* OR counter* OR peer-to-peer OR prevent* OR disrupt* OR stop* OR fight* OR redirect* OR “censoring hate content”) using search fields of AB, HW, ID, and TI 4. (comparison* OR quantitative OR quasi-experiment* OR survey* OR interview* OR poll* OR mixed-methods OR individual-level OR group-level OR control* OR experiment* OR study OR studies OR evaluat* OR MTurk OR longitudinal OR random* OR “digital method*” OR “machine learning” OR “natural language processing” OR multisectoral OR review*) using search fields of AB, HW, ID, and TI |

1. **DistillerSR’s AI software stopping rule**

We used DistillerSR software, which includes a machine learning feature that can learn from decisions made by human screeners during the title and abstract screening process. This AI feature pushes records forward based on prior screening decisions made by human screeners, it assesses the potential for any further studies being included, and it also provides an estimate of the percentage of potentially eligible studies within the data (Eggins et al., in press). According to Eggins and colleagues (in press), once a 95 percent estimate has been reached, iterative sets of 50 titles and abstracts should be screened until no more potentially eligible studies are included, at which stage the remaining records drop out of the screening process. We reached this stage of saturation after screening a total of 4,744 title and abstracts, the remaining 5,695 records were not processed any further.

Eggins, E., Wilson, D. B., Betts, J., Roetman, S., Chandler-Mather, N., Theroux., & Dawe, S. (in press). Psychological, pharmacological, and legal interventions for improving the outcomes of children with substance misusing parents. *Campbell Systematic Reviews*.

**Appendix B. Codebook Forms**

| **Study Level Coding Form**    This coding form is for each unique study. Note that a study may be reported in multiple manuscripts (publications, technical reports, etc.). Also, some reports may include the results for distinct studies, such as evaluations in different cities. Our unit-of-analysis for the meta-analysis is an independent study. No two studies should include any of the same participants. If there are multiple publications for the same study, use the most complete study as the primary study ID and all other related studies as cross reference IDs. (Note: -99 = "missing" / -98 = "not applicable" / -97 = "cannot tell"). | | | | |
| --- | --- | --- | --- | --- |
| **Identifiers** | | | |  |
| 1. | Reference ID | sl_studyid | | \|__\|__\|__\|__\|__\| |
| 2. | Other related references | sl_otherref | | \|__\|__\|__\|__\| |
| 3. | Modification date (dd/mm/yy) | sl_mdate | | \|__\|__\|__\|__\|__\|__\| |
|  |  |  | |  |
| **General Study Information** | |  | |  |
| 4. | Publication type  1. Book  2. Book chapter  3. Journal article (peer reviewed)  4. Journal article (not-peer reviewed)  5. Thesis-dissertation  6. Technical report  7. Conference paper  8. Government publication  9. Other (specify): __________ | sl_pubtype | | \|__\| |
| 5. | Language type of study | sl_language | | \|__\| |
|  | 1. English  2. German |  |  |  |
|  | 3. Other |  |  |  |
| 6. | Geographic location of study  1. North America  2. South America  3. Europe  4. Africa  5. Asia  6. Oceania  7. Unknown | sl_location | | \|__\| |
| 7. | Years of data collection |  | |  |
|  | Year data collection started | sl_datastart | | \|__\|__\|__\|__\| |
|  | Year data collection ended | sl_dataend | | \|__\|__\|__\|__\| |
| 8. | Intervention type | sl_inttype | | \|__\| |
|  | 1. Online only  2. Offline only  3. Mixed approach intervention  4. Unknown |  | |  |
| 9. | Researcher involvement  1. Researcher initiated intervention  2. Online platform-initiated intervention  3. Government initiated intervention  4. Non-profit/Advocacy Group  5. Unknown | sl_rinvolve | | \|__\| |
| 10. | Was this research funded by a grant or external agency | sl_funding | | \|__\| |
|  | 1. No  2. Yes  3. Unknown |  | |  |
|  |  |  | |  |
| **Research Design** | |  | |  |
| 11. | Unit of assignment to conditions  1. Individual  2. Incident (might include multiple comments)  3. Online platform  4. Online groups  5. Other (specify): __________  6. Unknown | sl_uoa | | \|__\| |
| 12. | Methodological approach | sl_method | | \|__\| |
|  | 1. Qualitative  2. Quantitative  3. Mixed methods |  |  |  |
| 13. | How subjects were assigned to condition (this is about assignment not sampling)  1. Randomly after matching, yoking, stratification, blocking, etc.  2. Randomly without matching  3. Regression discontinuity (quantitative cutting point defines groups)  4. Wait list control or other such quasi-random procedures (e.g., alternating cases)  5. Quasi-experimental, matched individual level  6. Quasi-experimental, matched group level (e.g., classrooms)  7. Quasi-experimental, statistical controls for baseline differences  8. Quasi-experimental, no statistical controls for baseline differences  9. Quasi-experimental, cohort design (historical controls)  10. Quasi-experimental, other (specify): __________ | sl_assign | | \|__\|__\| |
| 14. | If random assignment, did researchers:  1. Maintain the integrity of randomization or other assignment methods (no more than a few cases failed to end up in desired group)  2. Fail at randomization or other assignment methods  3. Unknown  4. Not applicable | sl_rndinteg | | \|__\| |
| 15. | [RISK OF BIAS] Is there any risk of selective outcome reporting bias? In other words, is there any evidence that the authors have not reported findings for all variables measured as part of this study?  1. Low risk  2. Some risk  3. High Risk | sl_selectrepb | | \|__\| |

| **Comparison Level Coding Form**    This coding form is for each treatment/comparison contrast coded from a study. For most studies, you will only code this form once. However, some studies may have two or more treatment conditions or two or more comparison conditions. In the coding below, it is critical to indicate if any of the treatment/comparison contrasts for a study share sample participants. For example, a study might have two distinct treatments but only one comparison group. In this case, these comparisons share sample participants (i.e., the same comparison condition). (Note: -99 = "missing" / -98 = "not applicable" / -97 = "cannot tell"). | | | |
| --- | --- | --- | --- |
| **Identifiers** | | |  |
| 1. | Reference ID | cl_studyid | \|__\|__\|__\|__\|__\| |
| 2. | Modification date (dd/mm/yy) | cl_datem | \|__\|__\|__\|__\|__\|__\| |
| 3. | Treatment group label | cl_txlabel | \|__\|__\|__\|__\|__\| |
| 4. | Control/comparison group label | cl_cglabel | \|__\|__\|__\|__\|__\| |
| **Sample Information** | |  |  |
| 5. | Treatment group sample size (at start of study before attrition) | cl_ctxn | \|__\|__\|__\|__\|__\| |
| 6. | Comparison group sample size (at start of study before attrition) | cl_ccgn | \|__\|__\|__\|__\|__\| |
| 7. | Mean or median age of sample | cl_meanage | \|__\|__\|.__\| |
| 8. | Youngest age in sample | cl_minage | \|__\|__\| |
| 9. | Oldest age in sample | cl_maxage | \|__\|__\| |
| 10. | Sex distribution for this treatment/comparison contrast  1. 76-100% Male  2. 51-75% Male  3. 26-50% Male  4. 0-25% Male  5. Unknown | cl_sex | \|__\| |
| 11. | Percent of this condition that is represented by whites? | cl_whites | \|__\| |
| 12. | Percent of this condition that is represented by Blacks/Africans/Caribbeans? | cl_blacks/africans/caribbeans | \|__\| |
| 13. | Percent of this condition that is represented by Hispanics (non-Whites)? | cl_hispanics (non-whites) | \|__\| |
| 14. | Percent of this condition that is represented by Asians? | cl_asians | \|__\| |
| 15. | Percent of this condition that is represented by mixed/multiple ethnic groups? | cl_mixed/multiple | \|__\| |
| 16. | Percent of this condition that is represented by other (specify)?: __________ | cl_other | \|__\| |
| **Nature of Treatment Condition** | |  |  |
| 17. | Type of intervention  1. Online hate detection only  2. Server shutdowns  3. Deletion of social media accounts  4. Responding to online hate  5. Modifying hateful content  6. Countering “fake news”  7. Twitter “fact” check  8. Other (specify): _________ | cl_inttype | \|__\| |
| 18. | Content of intervention  1. General online hate speech/cyberhate  2. Right-wing extremist content  3. Left-wing extremism content  4. Islamist extremist content  5. Multiple  6. Other (specify): _________  7. Unknown | cl_intcontent | \|__\| |
| 19a. | Location of intervention  1. Websites  2. Text messaging applications  3. Online and social media platforms  4. Other (specify): _________ | cl_intlocate | \|__\| |
| 19b. | If social media, which platform  1. Facebook  2. Instagram  3. TikTok  4. WhatsApp  5. Google  6. YouTube  7. Snapchat  8. Twitter  9. 4Chan  10. Gab  11. Other (specify): ______________  12. Not applicable | cl_platform | \|__\|__\| |
| 20. | Other elements of this condition: | cl_txother | \|__\| |
|  |  |  |  |
| **Nature of Comparison Condition** | |  |  |
| 21. | Type of comparison condition  1. No exposure  2. Comparison exposure  3. Other (specify): ______________ | cl_comptype | \|__\| |
| **Comparability of Conditions** | |  |  |
| 22.  23.  24. | Were the conditions compared for baseline equivalence on sex, either statistically or descriptively?  1. Statistically  2. Descriptively  3. Unknown  Were the conditions compared for baseline equivalence on race, either statistically or descriptively?  1. Statistically  2. Descriptively  3. Unknown  Were the conditions compared for baseline equivalence on age, either statistically or descriptively?  1. Statistically  2. Descriptively  3. Unknown | cl_baseddiffsex  cl_baseddiffrace  cl_baseddiffage | \|__\|  \|__\|  \|__\| |
| 25. | RISK OF BIAS ITEM: Based on the above, is there a risk of selection bias, that is, that the groups were different at baseline?  1. Low risk  2. Some risk  3. High risk | cl_selectbias | \|__\| |
| 26. | RISK OF BIAS ITEM: Is there a risk of general attrition bias for the primary outcome measure, that is, attrition in excess of 10%?  1. Low risk  2. Some risk  3. High risk | cl_attritiongen | \|__\| |
| 27. | RISK OF BIAS ITEM: Is there a risk of different attrition bias for the primary outcome measure, that is, meaningful differential attrition?  1. Low risk  2. Some risk  3. High risk | cl_attritiondiff | \|__\| |

| **Outcome (Dependent Variable) Coding Form**    Code each eligible outcome or dependent variable using the form below. Note that you should code this only once for a variable that is measured at multiple time points. That is, recidivism measured at 3, 6, and 9-months is a single dependent variable. Code the characteristics of the measure using this form and the data for each measurement time point on the effect size forms. (Note: -99 = "missing" / -98 = "not applicable" / -97 = "cannot tell"). | | | |
| --- | --- | --- | --- |
| **Identifiers** | | |  |
| 1. | Reference ID | dv_studyid | \|__\|__\|__\|__\|__\| |
| 2. | Modification date (dd/mm/yy) | dv_mdate | \|__\|__\|__\|__\|__\|__\| |
| 3. | Outcome ID | dv_dvid | \|__\|__\|__\|__\| |
| 4. | Dependent variable label | dv_dvlabel | \|__\|__\|__\|__\| |
| **Characteristics of Variable** | |  |  |
| 5. | Elements reported in this outcome measure irrespective of the type of incident and reporting source (check best one): | dv_elem | \|__\|__\| |
|  | 1. Global dichotomy or polychotomy (e.g., created, or consumed cyberhate, extremist content or non-extremist content=yes/no) |  |  |
|  | 2. Summed dichotomous (e.g., sum of “yes/no” on list of specific behaviors) |  |  |
|  | 3. Frequency or rate, (count of incident; incidents per 1000 persons) |  |  |
|  | 4. Scale (seriousness rating or index), see this often with self-report measures |  |  |
|  | 5. Event timing (e.g., days without content creation; time since last post, log on, video watch) |  |  |
|  | 6. Proportion or amount of time on extremist website, etc. |  |  |
|  | 7. Rating of amount of prejudice, severity, change, etc. This is similar to frequency but in rating form. (e.g., How often you did “x” behavior) |  |  |
|  | 8. More than one of above elements combined in composite measure |  |  |
|  | 9. Other |  |  |
|  | 10. Unknown |  |  |
| 6. | Type of behavior represented by this measure (what’s counted, irrespective of source of information and authors’ label or description of the measure) check best one: | dv_type | \|__\| |
|  | 1. Content creation (e.g., production and authorship of original content such as making videos, writing blog posts, or uploading content) |  |  |
|  | 2. Transmission of hate speech (e.g., racist, homophobic, anti-Semitic), not specifically restricted to extremist acts |  |  |
|  | 3. Consumption of cyberhate (e.g., watch videos, visit social media platforms, or read blogs without making accounts from self or observer’s report) |  |  |
|  | 4. Collecting extremist content (e.g., organize links and content for either their personal use or to disseminate information to others who are active online |  |  |
|  | 5. Critics (e.g., comment on social media posts, submit reviews, and rate content) |  |  |
|  | 6. Joiners (e.g., those who maintain accounts but do not comment or post publicly available content) |  |  |
|  | 7. Other |  |  |
|  | 8. Unknown |  |  |
| 7. | RISK OF BIAS ITEM: Person providing outcome data knows which condition the participant is in (i.e., is there a potential bias from the lack of anonymizing the assessor?)  1. Low risk  2. Some risk  3. High risk | dv_bias | \|__\| |

| **Effect Size Coding Form**  Code all effect sizes of interest using the form below, coding each effect size separately (i.e., with a different copy of the form or record in the database). Indicate the study ID, comparison ID, and dependent variable ID. Give each effect size within a study a unique ID (i.e., 1, 2, 3 ...). (Note: -99 = "missing" / -98 = "not applicable" / -97 = "cannot tell").  There are several ways to compute effect sizes using the different tabs. ONLY USE ONE METHOD per effect size. If you have the raw means and also a regression coefficient for the same outcome from a model that adjusts for baseline differences, these are two different effect sizes. The different effect size computation methods are:  1. Means and standard deviations  2. Means and standard errors  3. Frequency of failures in each condition  4. Proportion of failures in each condition  5. Logistic regression coefficient for treatment effect dummy code  6. OLS unstandardized regression coefficient  7. OLS standardized regression coefficient  8. Independent samples t-test  9. Chi-square test (2 by 2, df = 1)  10. Point-biserial correlation coefficient  11. Phi correlation coefficient  12. Hand computation (e.g., using the online effect size calculator) | | | |
| --- | --- | --- | --- |
| **Identifiers** | | |  |
| 1. | Reference ID | es_studyid | \|__\|__\|__\|__\|__\| |
| 2. | Modification date (dd/mm/yy) | es_mdate | \|__\|__\|__\|__\|__\|__\| |
| 3. | Comparison ID | es_compid | \|__\|__\|__\|__\| |
| 4. | Outcome ID | es_dvid | \|__\|__\|__\|__\| |
| 5. | Effect Size ID | es_id | \|__\|__\|__\|__\| |
| **Effect Size Information** | |  |  |
| 6. | Direction of effect  1. Favors treatment  2. Favors control  3. Neither, exactly equal  4. Unknown | es_direct | \|__\| |
| 7. | Type of effect size (i.e., baseline differences, first post treatment outcome measure, or a follow-up measure)  1. Baseline  2. Post-test  3. Follow-up | es_etype | \|__\| |
| 8. | Effect reported as statistically significant by authors  1. No  2. Yes  3. Unknown | es_essig | \|__\| |
| 9.  10.  11. | Mean timing of measurement (days captured by the measure from the point of assignment to conditions)  Minimum timing of measurement (days captured by the measure from the point of assignment to conditions)  Maximum timing of measurement (days captured by the measure from the point of assignment to conditions) | es_meantime  es_minitime  es_maxtime | \|__\|  \|__\|  \|__\| |
| **Effect Size Data** | |  |  |
| 12. | Treatment group sample size for this effect size | es_txn | \|__\|__\|__\|__\| |
| 13. | Comparison group sample size for this effect size | es_cgn | \|__\|__\|__\|__\| |
|  | **Mean Outcome Data** |  |  |
| 14. | Mean treatment group | es_mtx | \|__\|__\|__\|__\|.__\|__\| |
| 15. | Mean comparison group | es_mcg | \|__\|__\|__\|__\|.__\|__\| |
| 16. | Are the above means adjusted for baseline differences?  1. No  2. Yes  3. Unknown | es_madj | \|__\| |
| 17. | Standard deviation treatment group | es_sdtx | \|__\|__\|__\|__\|.__\|__\| |
| 18. | Standard deviation comparison group | es_sdcg | \|__\|__\|__\|__\|.__\|__\| |
| 19. | Standard error treatment group | es_setx | \|__\|__\|__\|__\|.__\|__\| |
| 20. | Standard error comparison group | es_secg | \|__\|__\|__\|__\|.__\|__\| |
|  | **Dichotomous outcome data** |  |  |
| 21. | Treatment group number successful | es_txn | \|__\|__\|__\|__\| |
| 22. | Comparison group number successful | es_cgn | \|__\|__\|__\|__\| |
| 23. | Treatment group number failures | es_txnf | \|__\|__\|__\|__\| |
| 24. | Comparison group number failures | es_cgnf | \|__\|__\|__\|__\| |
| 25. | Treatment group proportion of successes (only code this if raw frequencies are not available) | es_txpf | \|__\|.__\|__\|__\|__\|__\| |
| 26. | Comparison group proportion of successes (only code this if raw frequencies are not available) | es_cgpf | \|__\|.__\|__\|__\|__\|__\| |
| 27. | Are the above frequencies or proportions adjusted for baseline differences?  1. No  2. Yes  3. Unknown | es_padj | \|__\| |
|  | **Logistic regression** |  |  |
| 28. | Logistic regression coefficient (for treatment effect dummy) | es_lgor | \|__\|.__\|__\|__\|__\|__\| |
| 29. | Standard error for logistic regression coefficient | es_selgor | \|__\|.__\|__\|__\|__\|__\| |
| 30. | t-test or z-test for logistic regression coefficient | es_olst | \|__\|.__\|__\|__\|__\|__\| |
| 31. | Odds ratio for treatment effect dummy (optional) | es_or | \|__\|__\|__\|.__\|__\|__\| |
|  | **OLS regression** |  |  |
| 32. | Unstandardized regression coefficient | es_olsb | \|__\|.__\|__\|__\|__\|__\| |
| 33. | Standard regression coefficient | es_olsbeta | \|__\|.__\|__\|__\|__\|__\| |
| 34. | Standard error of regression coefficient | es_olsse | \|__\|.__\|__\|__\|__\|__\| |
| 35. | Standard deviation for dependent variable | es_sddv | \|__\|.__\|__\|__\|__\|__\| |
|  | **Other possible effect size data** |  |  |
| 36. | t-test (comparing two-sample means; not the t from a regression model) | es_t | \|__\|__\|__\|__\|.__\|__\| |
| 37. | p-value from a t-test (comparing two-sample means; not the t from a regression model) | es_pfromt | \|__\|.__\|__\|__\|__\|__\| |
| 38. | Correlation coefficient point-biserial (treatment versus comparison correlated with scaled variable) | es_rpb | \|__\|.__\|__\|__\|__\|__\| |
| 39. | Correlation coefficient phi (treatment versus comparison correlated with a dichotomous variable) | es_rphi | \|__\|.__\|__\|__\|__\|__\| |
| 40. | Chi-square (treatment versus comparison correlated with a dichotomous variable, df must equal 1) | es_chisq | \|__\|__\|__\|__\|.__\|__\| |
|  | **Effect size computed by hand (e.g., using online calculator)** |  |  |
| 41. | Standardized mean difference effect size computed by hand (d-type) | es_hand | \|__\|__\|.__\|__\|__\|__\| |
| 42. | Variance for standardized mean different effect size computed by hand | es_handv | \|__\|__\|.__\|__\|__\|__\| |
| 43. | Computed effect size | es_calc | \|__\|__\|.__\|__\|__\|__\| |
| 44. | Computed effect size standard error | es_calcse | \|__\|__\|.__\|__\|__\|__\| |
|  | **Effect size coding notes** |  |  |
| 45. | Page number where effect size data found | es_page | \|__\|__\|__\|__\|__\|__\| |

| **Appendix C. Characteristics of excluded studies – Study Level** | | | | | | | | | | | |  |
| --- | --- | --- | --- | --- | --- | --- | --- | --- | --- | --- | --- | --- |
| Study | Publication type | Research funded by grant/external agency | Study setting/context | | | Study design | | | | | |  |
|  |  |  | Geographic location | Language | Year of data collection | Methodological approach | Sample size | Unit of assignment | Subjects assigned to condition | Intervention type | Researcher involvement | |
| Boccanfuso et al. (2021) | Journal article | No | Australia | English | N/A | Quantitative | 114 subjects | Individual | Randomly without matching | Offline | Researcher initiated intervention | |
| Bozeman (2015) | Dissertation | No | United States | English | 2014 | Quantative | 309 college students | Individual | Random without matching | Online | Researcher initiated intervention | |
| Braddock (2019) | Journal article | No | United States | English | 2018 | Quantitative | 357 individuals | Individual | Randomly without matching | Online | Researcher initiated intervention | |
| Chandrasekharan et al. (2017) | Journal article | No | Unspecified | English | 2015 | Quantitative | 670 million posts | Individual | N/A | Online | Observation | |
| Davey et al. (2018) | Technical report | Yes - Facebook | Unspecified | English | 2015 | Quantitative | 800 individuals | Individual | N/A | Online | Researcher initiated intervention | |
| Döring (2001) | Journal article | No | Unspecified | German | N/A | Qualitative and quantitative | 24 chat forums, 3,120 participants, 244 active sanctions | Forums; sanctions | Unrandomized | Online only | Observation | |
| Fiala & Husovec (2018) | Journal article | Yes - TILEC and the Centerlab | N/A | English | 2018, 2019 | Quantitative | 148 subjects | Individual | Randomly without matching | Online only | Researcher initiated intervention | |
| Forscher et al. (2017) | Journal article | Yes – NIH and national science foundation graduate research fellowship | United States | English | N/A | Quantitative | 302 subjects | Individual | Randomly without matching | Online and offline | Researcher initiated intervention | |
| Frischlich et al. (2018) | Journal article | Yes – Federal Ministry of Interior, Germany | Germany | English | N/A | Quantitative | 338; 155 subjects | Individual | Randomly without matching | Online and offline | Observation | |
| Kazerooni et al. (2018) | Journal article | No | United States | English | N/A | Quantitative | 156 subjects | Individual | Randomly without matching | Online | Observation | |
| Study | Publication type | Research funded by grant/external agency | Study setting/context | Study design | | | | | |  |  | |
|  |  |  | Geographic location | Language | Year of data collection | Methodological approach | Sample size | Unit of assignment | Subjects assigned to condition | Intervention type | Researcher involvement | |
| Kloss (2020) | Dissertation | No | United States | English | 2018 | Quantitative | 142 subjects | Individual | Randomly without matching | Online | Researcher initiated intervention | |
| Marcowicz (2009) | Dissertation | No | United States | English | 2008 | Quantitative | 74 subjects | Individual | Randomly without matching | Offline | Observation | |
| Matias (2019) | Journal article | No | Unspecified | English | 2016 | Quantitative | 18,264 comments | Individual | Random | Online | Researcher initiated intervention | |
| Munger (2017) | Journal article | No | Unspecified | English | 2015 | Quantitative | 242 subjects | Individual | Randomly without matching | Online | Researcher initiated intervention | |
| Ribeiro et al. (2020) | Journal article | No | Unspecified | English | 2020 | Quantitative | 3 million reddit posts; 2.9 million website posts | Posts; individual users; community forums | N/A | Online only | Observation of community intervention | |
| Shortland et al. (2020) | Journal article | No | United States | English | N/A | Quantitative | 1112 subjects | Individual | Randomly without matching | Online only | Observation | |
| Siegal & Badaan (2020) | Journal article | No | ‘Arab Twittersphere’; Lebanon | English | 2018; 2017 | Quantitative | 9,957 subjects;500 subjects | Individual | Randomly without matching | Online only | Researcher initiated intervention | |
| Simonovitz et al. (2017) | Journal article | No | Hungary | English | 2009 | Quantitative | 579 subjects | Individual | Randomly without matching | Online only | Researcher initiated intervention | |
| Stiff & Kedra (2020) | Journal article | No | United Kingdom | English | N/A | Quantitative | 80 subjects | Individual | N/A | Online | Observation | |
| van Lær (2014) | Journal article | No | United Kingdom | English | N/A | Quantitative | 124; 233 subjects | Individual | Random | Online and offline | Researcher initiated intervention | |
| Wei et al. (2020) | Journal article | No | United States | English | 2018 | Quantitative | 102,094 users, 31,210, 740 tweets | Individual | N/A | Offline | Observation of community intervention | |

| **Appendix D. Characteristics of excluded studies – Comparison level** | | | | | | | | | | | |
| --- | --- | --- | --- | --- | --- | --- | --- | --- | --- | --- | --- |
| Study | Demographics | | | | Sample size | | Intervention | | | | Outcome |
|  | Age of those included in sample | | Sex distribution | Ethnicity/nationality* | Treatment group | Comparison group | Type | Content | Location | Comparison condition |  |
|  | Youngest | Oldest |  |  |  |  |  |  |  |  |  |
| Boccanfuso et al. (2021) | 18 | N/A | 23% male | 32.5%  Anglo-Celtic,  25.4% East Asian, 18.4% South-East Asian,  17.5% European,  5.3% South Asian, 4.4% Arabic or  Middle Eastern,  1.8% West Asian, 0.9% Latinx,  0.9% African,  2.6% Other,  0.9% Prefer not to answer | 47 | 67 | Computer mediated intergroup contact ‘e-contact’ | Stigma | Online text-chat program | Comparison exposure (control group) | Reduction in stigma |
| Bozeman (2015) | 18 | 28 | 33% male | 60.5% White  0.7 American Indian  1.9% bi-racial  2.3% Black  2.3% Native Hawaiian  4.5% East Asian  7.4% Hispanic  9.4% South Asian  2.3 Other | 143 | 144 | Computer mediated written conversation | Bi-stander effect | Offline | Comparison exposure (control group) | Increase of confrontation |
| Braddock (2019) | 18 | 35 | 52.7% male | 63.6% Non-Hispanic Caucasian/White  14.6% African American/Black  10.6% Hispanic  5.0% Asian  2.2% Native American  1.7% Other  2.3% Did not disclose | N/A | N/A | Attitudinal inoculation | Radicalization | Online survey | Comparison exposure (control group) | Evidence of inoculation as effective intervention |
| Chandrasekharan et al. (2017) | N/A | N/A | N/A | N/A | 340,093; 270,435 users | 340,093; 270,435 users | Banning | Hate speech | Reddit | Comparison exposure (control group) | Banning as effective measure on reddit |
| Davey et al. (2018) | N/A | N/A | N/A | N/A | N/A | N/A | Online outreach | Radicalization | Facebook | N/A | Counter conversations programme |
| Study | Demographics | | | | Sample size | | Intervention | | | | Outcome |
|  | Age of those included in sample | | Sex distribution | Ethnicity/nationality* | Treatment group | Comparison group | Type | Content | Location | Comparison condition |  |
|  | Youngest | Oldest |  |  |  |  |  |  |  |  |  |
| Döring (2001) | N/A | N/A | N/A | N/A | N/A | N/A | Intra-community control of each other’s behaviour | Multiple | Chat forums | N/A | Intra-community behaviour control insights |
| Fiala & Husovec (2018) | N/A | N/A | 43% male | N/A | 76 | 72 | Independent alternative dispute resolution | Multiple | Social media platform simulation | Comparison exposure (baseline group) | Improved accuracy to takedown decisions |
| Forscher et al. (2017) | N/A | N/A | 32% male | Phase 1: 67% White, 25% Asian  Phase 2: 73% White, 19% Asian | 136 | 156 | Habit breaking intervention | Prejudice | Online survey | Comparison exposure (control group) | Evidence for habit-breaking intervention effectiveness |
| Frischlich et al. (2018) | 16; 18 | 55; 62 | Study 1: 52.1% male; Study 2: 56.1% male | Study 1: 52% first or second generation migrant  Study 2: 70% migrant background | 338; 155 | 338; 155 | Narrativity | Extremism | Lab simulation and online | Comparison exposure (baseline measures) | Insight into role of narrativity in extremist propaganda and counter violent extremism |
| Kazerooni et al. (2018) | 18 | 33 | 35.3% male | 24.1% Asian,  5.3% African American,  9.8% Latino/Hispanic, 2.3% Middle Eastern,  9.0% Mixed-race, 48.9% White,  1% Other | N/A | N/A | Bystander intervention | Multiple | Twitter | N/A | Insight into likelihood of cyber-bystander intervention |
| Kloss (2020) | 21 | 60+ | 27.5% male | 84.0% White/Caucasian,  8.2% Black or African American,  3.4% Hispanic/Latino,  2.3% Asian/Pacific Islander,  0.52% Native American  1.4% Multiple Ethnicity/Other | 57 | 85 | Social issues advocacy scale | Attitude towards lesbian and gay individuals | Online survey | Comparison exposure (control group) | No significant effect of treatment |
| Study | Demographics | | | | Sample Size | | Intervention | | | | Outcome |
|  | Age of those included in sample | | Sex distribution | Ethnicity/nationality* | Treatment group | Control group | Type | Content | Location | Comparison condition |  |
|  | Youngest | Oldest |  |  |  |  |  |  |  |  |  |
| Marcowicz (2009) | N/A | N/A | 45.9-54.1% male | 32.4% Black/African American, 16.2% Hispanic/ Latino, 0% American Indian/Alaska Native, 24.3% White, 2.7% Native Hawaiian/Other Pacific Islander, 18.9% Asian, 5.4% Other | 37 | 37 | Intergroup dialogue | Prejudice | In-person dialogue | Comparison exposure (control group) | Insight into intergroup contact impact on colour-blind racism |
| Matias (2019) | N/A | N/A | N/A | N/A | N/A | N/A | Community rules | Online harassment | Reddit | N/A | Insights into rule compliance as moderation measure |
| Munger (2017) | N/A | N/A | 100% male | 100% White | 191 | 51 | Group norm promotion and social sanctioning | Multiple | Twitter | Comparison exposure (control group) | Contribution to random field work experiment methodology |
| Ribeiro et al. (2020) | N/A | N/A | N/A | N/A | N/A | N/A | Community-level moderation | Radicalization | Reddit; online forums | Comparison exposure (baseline group) | Community migration insights |
| Shortland et al. (2020) | 18 | 26 | 82.45% male | 58.00% White,  11.51% Black or African American, 10.52% Hispanic/Latino, 17.09% Asian,  2.88% Other | N/A | N/A | The militant extremist mindset (MEM) scale | Radicalization | Online survey | Comparison exposure (baseline and control measures) | Insight into role of individual aggression in tendency for extremism |
| Siegal & Badaan (2020) | 18 | N/A | N/A | ‘Arabic’/Lebanese citizens | 795; N/A | N/A; N/A | Elite-endorsement with common religious identity | Hate speech | Twitter; survey | Comparison exposure (control group) | Content creation |
| Simonovitz et al. (2017) | 24 | 26 | 47.5% male | 1.2% Roma | 292 | 287 | Perspective-taking game | Prejudice attitude | Online | Comparison exposure (control group) | Template for low-cost interventions |
| Study | Demographics | | | | Sample Size | | Intervention | | | | Outcome |
|  | Age of those included in sample | | Sex distribution | Ethnicity/nationality* | Treatment group | Control group | Type | Content | Location | Comparison condition |  |
| Stiff & Kedra (2020) | N/A | N/A | 25% male | N/A | N/A | N/A | Outgroup interaction via video game | Prejudice | Online video game | N/A | Reduction in prejudice |
| van Lær (2014) | 19; 19 | 38; 35 | Study 1: 60.5% male; Study 2: 51.5% male | N/A | N/A | N/A | Perception of justice | Online harassment | Computer lab | N/A | Relatability and storytelling improve acceptance of network provider intervention |
| Wei et al. (2020) | N/A | N/A | N/A | N/A | 71,919 users,  889,579 tweets | N/A | Protest | Hate speech | Twitter | Comparison exposure (baseline group) | Online prejudice classifier |
| Note: *Some percentages may not add up to 100% due to participants identifying with more than one ethnicity or due to rounding. Other studies with percentages not reaching 100% or studies with no indication of percentages on ethnicity/nationality did not provide any further information. | | | | | | | | | | | |

| **Appendix E. Reasons studies were excluded during full-text coding** | |
| --- | --- |
| **Reason for exclusion** | **Study** |
| Studies lacked the necessary information to complete the meta-analysis | Chandrasekharan et al. (2017) |
|  | Davey et al. (2018) |
|  | Matias (2019) |
|  | Munger (2017) |
|  | Ribeiro et al. (2020) |
|  | Siegel & Badaan (2020) |
| Studies included an online intervention that focused more on the effects of media exposure on cognition than countering the transmission, creation, and/or consumption of online hate speech | Braddock (2019) |
|  | Kloss (2020) |
|  | Shortland et al. (2020) |
|  | Wei et al. (2020) |
| Studies did not meet inclusion criteria (i.e., experimental, online intervention aimed at reducing online hate speech) | Boccanfuso et al. (2020) |
|  | Bozeman (2015)  Döring (2001) |
|  | Fiala & Husovec (2018) |
|  | Forscher et al. (2017) |
|  | Frischlich et al. (2018) |
|  | Kazerooni et al. (2018) |
|  | Markowicz (2010) |
|  | Simonovits et al. (2018) |
|  | Stiff & Kedra (2020) |
|  | van Laer (2014) |

**Appendix F. STATA Log File**

------------------------------------------------------------------------------------------------------------------------------------------

name: cleaning

log: C:\Users\ajima\Dropbox\projects\hspeechSR\data\cleaning.log

log type: text

opened on: 2 Aug 2021, 11:23:58

.

. // Importing data

. import excel using "hsdata.xlsx", firstrow case(lower)

(113 vars, 20 obs)

. describe

Contains data

Observations: 20

Variables: 113

------------------------------------------------------------------------------------------------------------------------------------------

Variable Storage Display Value

name type format label Variable label

------------------------------------------------------------------------------------------------------------------------------------------

refid str5 %9s Refid

user str17 %17s User

level str1 %9s Level

tas_study byte %10.0g tas_study

tas_focus byte %10.0g tas_focus

tas_interven byte %10.0g tas_interven

egs_study byte %10.0g egs_study

egs_language byte %10.0g egs_language

egs_method byte %10.0g egs_method

egs_populate byte %10.0g egs_populate

egs_compcond byte %10.0g egs_compcond

egs_randassign byte %10.0g egs_randassign

egs_matching byte %10.0g egs_matching

egs_timeseries byte %10.0g egs_timeseries

sl_form_k str5 %9s SL_Form_k

sl_studyid str5 %9s sl_studyid

sl_pubtype str11 %11s sl_pubtype

sl_language str12 %12s sl_language

sl_location str12 %12s sl_location

sl_datastart str4 %9s sl_datastart

sl_dataend str4 %9s sl_dataend

sl_inttype str11 %11s sl_inttype

sl_rinvolve str12 %12s sl_rinvolve

sl_funding str11 %11s sl_funding

sl_uoa str7 %9s sl_uoa

sl_method str10 %10s sl_method

sl_assign str10 %10s sl_assign

sl_rndinteg str12 %12s sl_rndinteg

sl_selectrepb str14 %14s sl_selectrepb

cl_form_k str24 %24s CL_Form_k

cl_studyid str5 %9s cl_studyid

cl_txlabel str18 %18s cl_txlabel

cl_cglabel str8 %9s cl_cglabel

cl_ctxn str3 %9s cl_ctxn

cl_ccgn str3 %9s cl_ccgn

cl_meanage str3 %9s cl_meanage

cl_minage str2 %9s cl_minage

cl_maxage str3 %9s cl_maxage

cl_sex str7 %9s cl_sex

cl_whites str3 %9s cl_whites

cl_blacksafri~s str3 %9s cl_blacks/africans/caribbeans

cl_hispanicsn~s str3 %9s cl_hispanics (non-whites)

cl_asians str3 %9s cl_asians

cl_mixedmulti~e str3 %9s cl_mixed/multiple

cl_other str9 %9s cl_other

cl_inttype str11 %11s cl_inttype

cl_inttype_co~t str24 %24s cl_inttype_comment

cl_intcontent str14 %14s cl_intcontent

cl_intlocate str13 %13s cl_intlocate

cl_intlocate_~t str38 %38s cl_intlocate_comment

cl_platform str13 %13s cl_platform

cl_platform_c~t str37 %37s cl_platform_comment

cl_txother str202 %202s cl_txother

cl_comptype str12 %12s cl_comptype

cl_basediffsex str15 %15s cl_basediffsex

cl_basediffrace str16 %16s cl_basediffrace

cl_basediffage str15 %15s cl_basediffage

cl_selectionb~s str14 %14s cl_selectionbias

cl_attritiongen str13 %13s cl_attritiongen

cl_attritiond~f str17 %17s cl_attritiondiff

dv_form_k str26 %26s DV_Form_k

dv_studyid str5 %9s dv_studyid

dv_dvid str1 %9s dv_dvid

dv_dvlabel str41 %41s dv_dvlabel

dv_elem str8 %9s dv_elem

dv_type str8 %9s dv_type

dv_type_comment str28 %28s dv_type_comment

dv_bias str8 %9s dv_bias

es_form_k str28 %28s ES_Form_k

es_studyid str5 %9s es_studyid

es_compid str1 %9s es_compid

es_dvid str1 %9s es_dvid

es_id str1 %9s es_id

es_direct str10 %10s es_direct

es_etype str9 %9s es_etype

es_essig str9 %9s es_essig

es_meantime str3 %9s es_meantime

es_mintime str3 %9s es_mintime

es_maxtime str3 %9s es_maxtime

es_txn str3 %9s es_txn

es_cgn str3 %9s es_cgn

es_mtx str4 %9s es_mtx

es_mcg str4 %9s es_mcg

es_madj str8 %9s es_madj

es_sdtx str4 %9s es_sdtx

es_sdcg str4 %9s es_sdcg

es_setx str6 %9s es_setx

es_secg str6 %9s es_secg

ck str3 %9s es_txn

cl str3 %9s es_cgn

es_txnf str3 %9s es_txnf

es_cgnf str3 %9s es_cgnf

es_txpf str4 %9s es_txpf

es_cgpf str4 %9s es_cgpf

es_padj str8 %9s es_padj

es_lgor str3 %9s es_lgor

es_selgor str3 %9s es_selgor

es_olst str3 %9s es_olst

es_or str3 %9s es_or

es_olsb str5 %9s es_olsb

es_olsbeta str3 %9s es_olsbeta

es_olsse str4 %9s es_olsse

es_sddv str4 %9s es_sddv

es_t str3 %9s es_t

es_pfromt str4 %9s es_pfromt

es_rpb str3 %9s es_rpb

es_rphi str3 %9s es_rphi

es_chisq str3 %9s es_chisq

es_hand str6 %9s es_hand

es_handv str8 %9s es_handv

es_calc str3 %9s es_calc

es_calcse str3 %9s es_calcse

es_page str20 %20s es_page

------------------------------------------------------------------------------------------------------------------------------------------

Sorted by:

Note: Dataset has changed since last saved.

. drop in 8/17

(10 observations deleted)

. drop level - sl_studyid cl_studyid dv_studyid es_studyid

. browse

.

. // Converting string variables to numeric

. *destring sl_studyid, generate(sl_studyid_new) ignore("sl_studyid") force

. destring sl_pubtype, generate(sl_pubtype_new) ignore("sl_pubtype") force

sl_pubtype: characters s l _ p u b t y e removed; sl_pubtype_new generated as byte

. destring sl_language, generate(sl_language_new) ignore("sl_language") force

sl_language: characters s l _ a n g u e removed; sl_language_new generated as byte

. destring sl_location, generate(sl_location_new) ignore("sl_location") force

sl_location: characters s l _ o c a t i n removed; sl_location_new generated as byte

. destring sl_inttype, generate(sl_inttype_new) ignore("sl_inttype") force

sl_inttype: characters s l _ i n t y p e removed; sl_inttype_new generated as byte

. destring sl_rinvolve, generate(sl_rinvolve_new) ignore("sl_rinvolve") force

sl_rinvolve: characters s l _ r i n v o e removed; sl_rinvolve_new generated as byte

. destring sl_funding, generate(sl_funding_new) ignore("sl_funding") force

sl_funding: characters s l _ f u n d i g removed; sl_funding_new generated as byte

. destring sl_uoa, generate(sl_uoa_new) ignore("sl_uoa") force

sl_uoa: characters s l _ u o a removed; sl_uoa_new generated as byte

. destring sl_method, generate(sl_method_new) ignore("sl_method") force

sl_method: characters s l _ m e t h o d removed; sl_method_new generated as byte

. destring sl_assign, generate(sl_assign_new) ignore("sl_assign") force

sl_assign: characters s l _ a i g n removed; sl_assign_new generated as byte

. destring sl_rndinteg, generate(sl_rndinteg_new) ignore("sl_rndinteg") force

sl_rndinteg: characters s l _ r n d i t e g removed; sl_rndinteg_new generated as byte

. destring sl_selectrepb, generate(sl_selectrepb_new) ignore("sl_selectrepb") force

sl_selectrepb: characters s l _ e c t r p b removed; sl_selectrepb_new generated as byte

. *destring cl_txlabel, generate(cl_txlabel_new) ignore("cl_txlabel") force

. *destring cl_cglabel, generate(cl_cglabel_new) ignore("cl_cglabel") force

. destring cl_sex, generate(cl_sex_new) ignore("cl_sex") force

cl_sex: characters c l _ s e x removed; cl_sex_new generated as byte

. destring cl_inttype, generate(cl_inttype_new) ignore("cl_inttype") force

cl_inttype: characters c l _ i n t y p e removed; cl_inttype_new generated as byte

. destring cl_intcontent, generate(cl_intcontent_new) ignore("cl_intcontent") force

cl_intcontent: characters c l _ i n t o e removed; cl_intcontent_new generated as byte

. destring cl_intlocate, generate(cl_intlocate_new) ignore("cl_intlocate") force

cl_intlocate: characters c l _ i n t o a e removed; cl_intlocate_new generated as byte

. destring cl_platform, generate(cl_platform_new) ignore("cl_platform") force

cl_platform: characters c l _ p a t f o r m removed; cl_platform_new generated as byte

. destring cl_comptype, generate(cl_comptype_new) ignore("cl_comptype") force

cl_comptype: characters c l _ o m p t y e removed; cl_comptype_new generated as byte

. destring cl_basediffsex, generate(cl_basediffsex_new) ignore("cl_basediffsex") force

cl_basediffsex: characters c l _ b a s e d i f x removed; cl_basediffsex_new generated as byte

. destring cl_basediffrace, generate(cl_basediffrace_new) ignore("cl_basediffrace") force

cl_basediffrace: characters c l _ b a s e d i f r removed; cl_basediffrace_new generated as byte

. destring cl_basediffage, generate(cl_basediffage_new) ignore("cl_basediffage") force

cl_basediffage: characters c l _ b a s e d i f g removed; cl_basediffage_new generated as byte

. destring cl_selectionbias, generate(cl_selectionbias_new) ignore("cl_selectionbias") force

cl_selectionbias: characters c l _ s e t i b a removed; cl_selectionbias_new generated as byte

. destring cl_attritiongen, generate(cl_attritiongen_new) ignore("cl_attritiongen") force

cl_attritiongen: characters c l _ a t r i o n removed; cl_attritiongen_new generated as byte

. destring cl_attritiondiff, generate(cl_attritiondiff_new) ignore("cl_attritiondiff") force

cl_attritiondiff: characters c l _ a t r i o n d f removed; cl_attritiondiff_new generated as byte

. destring dv_elem, generate(dv_elem_new) ignore("dv_elem") force

dv_elem: characters d v _ e l m removed; dv_elem_new generated as byte

. destring dv_type, generate(dv_type_new) ignore("dv_type") force

dv_type: characters d v _ t y p e removed; dv_type_new generated as byte

. destring dv_bias, generate(dv_bias_new) ignore("dv_bias") force

dv_bias: characters d v _ b i a s removed; dv_bias_new generated as byte

. destring es_direct, generate(es_direct_new) ignore("es_direct") force

es_direct: characters e s _ d i r c t removed; es_direct_new generated as byte

. destring es_etype, generate(es_etype_new) ignore("es_etype") force

es_etype: characters e s _ t y p removed; es_etype_new generated as byte

. destring es_essig, generate(es_essig_new) ignore("es_essig") force

es_essig: characters e s _ i g removed; es_essig_new generated as byte

. destring es_madj, generate(es_madj_new) ignore("es_madj") force

es_madj: characters e s _ m a d j removed; es_madj_new generated as byte

(4 missing values generated)

. destring es_padj, generate(es_padj_new) ignore("es_padj") force

es_padj: characters e s _ p a d j removed; es_padj_new generated as byte

(5 missing values generated)

.

. destring refid, replace

refid: all characters numeric; replaced as int

. destring sl_datastart, replace

sl_datastart: all characters numeric; replaced as int

. destring sl_dataend, replace

sl_dataend: all characters numeric; replaced as int

. destring cl_ctxn, replace

cl_ctxn: all characters numeric; replaced as int

. destring cl_ccgn, replace

cl_ccgn: all characters numeric; replaced as int

. destring cl_meanage, replace

cl_meanage: all characters numeric; replaced as byte

. destring cl_minage, replace

cl_minage: all characters numeric; replaced as byte

. destring cl_maxage, replace

cl_maxage: all characters numeric; replaced as byte

. destring cl_whites, replace

cl_whites: all characters numeric; replaced as byte

. destring cl_blacksa~s, replace

cl_blacksafricanscaribbeans: all characters numeric; replaced as byte

. destring cl_hispani~s, replace

cl_hispanicsnonwhites: all characters numeric; replaced as byte

. destring cl_asians, replace

cl_asians: all characters numeric; replaced as byte

. destring cl_mixedmu~e, replace

cl_mixedmultiple: all characters numeric; replaced as byte

. destring cl_other, replace

cl_other: contains nonnumeric characters; no replace

. destring cl_inttype~t, replace

cl_inttype_comment: contains nonnumeric characters; no replace

. destring cl_intloca~t, replace

cl_intlocate_comment: contains nonnumeric characters; no replace

. destring cl_platfor~t, replace

cl_platform_comment: contains nonnumeric characters; no replace

. destring cl_txother, replace

cl_txother: contains nonnumeric characters; no replace

. destring dv_dvid, replace

dv_dvid: all characters numeric; replaced as byte

. destring dv_dvlabel, replace

dv_dvlabel: contains nonnumeric characters; no replace

. destring dv_type_co~t, replace

dv_type_comment: contains nonnumeric characters; no replace

. destring es_compid, replace

es_compid: all characters numeric; replaced as byte

. destring es_dvid, replace

es_dvid: all characters numeric; replaced as byte

. destring es_id, replace

es_id: all characters numeric; replaced as byte

. destring es_meantime, replace

es_meantime: all characters numeric; replaced as byte

. destring es_mintime, replace

es_mintime: all characters numeric; replaced as byte

. destring es_maxtime, replace

es_maxtime: all characters numeric; replaced as byte

. destring es_txn, replace

es_txn: all characters numeric; replaced as int

. destring es_cgn, replace

es_cgn: all characters numeric; replaced as int

. destring es_mtx, replace

es_mtx: all characters numeric; replaced as double

(4 missing values generated)

. destring es_mcg, replace

es_mcg: all characters numeric; replaced as double

(4 missing values generated)

. destring es_sdtx, replace

es_sdtx: all characters numeric; replaced as double

(4 missing values generated)

. destring es_sdcg, replace

es_sdcg: all characters numeric; replaced as double

(4 missing values generated)

. destring es_setx, replace

es_setx: all characters numeric; replaced as double

(6 missing values generated)

. destring es_secg, replace

es_secg: all characters numeric; replaced as double

(6 missing values generated)

. destring ck, replace

ck: all characters numeric; replaced as byte

(9 missing values generated)

. destring cl, replace

cl: all characters numeric; replaced as byte

(9 missing values generated)

. destring es_txnf, replace

es_txnf: all characters numeric; replaced as byte

(9 missing values generated)

. destring es_cgnf, replace

es_cgnf: all characters numeric; replaced as byte

(9 missing values generated)

. destring es_txpf, replace

es_txpf: all characters numeric; replaced as double

(5 missing values generated)

. destring es_cgpf, replace

es_cgpf: all characters numeric; replaced as double

(5 missing values generated)

. destring es_lgor, replace

es_lgor: all characters numeric; replaced as byte

(9 missing values generated)

. destring es_selgor, replace

es_selgor: all characters numeric; replaced as byte

(9 missing values generated)

. destring es_olst, replace

es_olst: all characters numeric; replaced as byte

(9 missing values generated)

. destring es_or, replace

es_or: all characters numeric; replaced as byte

(9 missing values generated)

. destring es_olsb, replace

es_olsb: all characters numeric; replaced as double

(6 missing values generated)

. destring es_olsbeta, replace

es_olsbeta: all characters numeric; replaced as byte

(9 missing values generated)

. destring es_olsse, replace

es_olsse: all characters numeric; replaced as double

(6 missing values generated)

. destring es_sddv, replace

es_sddv: all characters numeric; replaced as double

(6 missing values generated)

. destring es_t, replace

es_t: all characters numeric; replaced as byte

(9 missing values generated)

. destring es_pfromt, replace

es_pfromt: all characters numeric; replaced as double

(7 missing values generated)

. destring es_rpb, replace

es_rpb: all characters numeric; replaced as byte

(9 missing values generated)

. destring es_rphi, replace

es_rphi: all characters numeric; replaced as byte

(9 missing values generated)

. destring es_chisq, replace

es_chisq: all characters numeric; replaced as byte

(9 missing values generated)

. destring es_hand, replace

es_hand: all characters numeric; replaced as double

(7 missing values generated)

. destring es_handv, replace

es_handv: all characters numeric; replaced as double

(7 missing values generated)

. destring es_calc, replace

es_calc: all characters numeric; replaced as byte

(9 missing values generated)

. destring es_calcse, replace

es_calcse: all characters numeric; replaced as byte

(9 missing values generated)

.

. drop sl_pubtype sl_language sl_location sl_inttype sl_rinvolve sl_funding sl_uoa sl_method sl_assign sl_rndinteg sl_selectrepb cl_txlabe

> l cl_cglabel cl_sex cl_inttype cl_intcontent cl_intlocate cl_platform cl_comptype cl_basediffsex cl_basediffrace cl_basediffage cl_selec

> tionbias cl_attritiongen cl_attritiondiff dv_elem dv_type dv_bias es_direct es_etype es_essig es_madj es_padj cl_form_k dv_form_k es_for

> m_k ck cl

.

. rename *_new *

. rename user author

. replace author = "Bodine-Baron et al. (2020)" in 1/7

variable author was str17 now str26

(7 real changes made)

. replace author = "Alvarez-Benjumea & Winter (2018)" in 8/10

variable author was str26 now str32

(3 real changes made)

.

. // Labeling variables and values

.

. *Study level

.

. label variable refid "Reference ID"

. label define refid 13715 "Bodine-Baron et al. (2020)" 14921 "Alvarez-Benjumea & Winter (2018)"

. label value refid refid

.

. label variable author "Author"

.

. label variable sl_pubtype "Publication type"

. label define sl_pubtype 1 "Book" 2 "Book chapter" 3 "Journal article (peer reviewed)" 4 "Journal article (non-peer reviewed)" 5 "Thesis-

> dissertation" 6 "Technical report" 7 "Conference paper" 8 "Government publication" 9 "Other (specify)"

. label value sl_pubtype sl_pubtype

.

. label variable sl_language "Language type of study"

. label define sl_language 1 "English" 2 "German" 3 "Korean" 4 "Other"

. label value sl_language sl_language

.

. label variable sl_location "Location of study"

. label define sl_location 1 "North America" 2 "South America" 3 "Europe" 4 "Africa" 5 "Asia" 6 "Oceania" 7 "Unknown"

. label value sl_location sl_location

.

. label variable sl_datastart "Year data collection started"

.

. label variable sl_dataend "Year data collection ended"

.

. label variable sl_inttype "Intervention type"

. label define sl_inttype 1 "Online only" 2 "Offline only" 3 "Mixed-approach" 4 "Unknown"

. label value sl_inttype sl_inttype

.

. label variable sl_rinvolve "Researcher involvement"

. label define sl_rinvolve 1 "Researcher-initiated" 2 "Online platform" 3 "Gov't-initiated" 4 "Non-profit" 5 "Unknown"

. label value sl_rinvolve sl_rinvolve

.

. label variable sl_funding "Externally funded?"

. label define sl_funding 1 "No" 2 "Yes" 3 "Unknown"

. label value sl_funding sl_funding

.

. label variable sl_uoa "Unit of assignment"

. label define sl_uoa 1 "Individual" 2 "Incident (multiple comments)" 3 "Online platform" 4 "Online groups" 5 "Other" 6 "Unknown"

. label value sl_uoa sl_uoa

.

. label variable sl_method "Methods"

. label define sl_method 1 "Quantitative" 2 "Qualitative" 3 "Mixed methods"

. label value sl_method sl_method

.

. label variable sl_assign "Subject assignment"

. label define sl_assign 1 "Randomly, matching, etc." 2 "Randomly w/o matching" 3 "Regression discontinuity" 4 "Wait list control/quasi-ra

> ndom" 5 "Quasi-experimental, matched individual" 6 "Quasi-experimental, matched group" 7 "Quasi-experimental, statistical control" 8 "Qu

> asi-experimental, no statistical control" 9 "Quasi-experimental, cohort design" 10 "Quasi-experimental, other"

. label value sl_assign sl_assign

.

. label variable sl_rndinteg "Random assignment integrity"

. label define sl_rndinteg 1 "Maintained" 2 "Failures" 3 "Unknown" 4 "Not applicable"

. label value sl_rndinteg sl_rndinteg

.

. label variable sl_selectrepb "Risk of selective outcome reporting?"

. label define sl_selectrepb 1 "Low risk" 2 "Some risk" 3 "High risk"

. label value sl_selectrepb sl_selectrepb

.

. *Condition level

.

. *label variable cl_txlabel "Treatment"

.

. *label variable cl_cglabel "Control"

.

. label variable cl_ctxn "Treatment group N"

.

. label variable cl_ccgn "Comparison group N"

.

. label variable cl_meanage "Mean age"

.

. label variable cl_minage "Youngest age"

.

. label variable cl_maxage "Oldest age"

.

. label variable cl_sex "Sex distribution"

. label define cl_sex 1 "76-100%" 2 "51-75%" 3 "26-50%" 4 "0-25%"

. label value cl_sex cl_sex

.

. label variable cl_whites "% Whites"

.

. label variable cl_blacksafricanscaribbeans "% Blacks/Africans/Caribbeans"

.

. label variable cl_hispanicsnonwhites "% Hispanics (non-White)"

.

. label variable cl_asians "% Asians"

.

. label variable cl_mixedmultiple "% mixed/multiple ethnic groups"

.

. label variable cl_other "% other"

.

. label variable cl_inttype "Type of intervention"

. label define cl_inttype 1 "Online hate detection only" 2 "Server shutdowns" 3 "Deletion of social media accounts" 4 "Responding to onlin

> e hate" 5 "Modifying hateful content" 6 "Countering 'fake news'" 7 "Twitter 'fact' check" 8 "Other"

. label value cl_inttype cl_inttype

.

. label variable cl_inttype_comment "Type of intervention (Other)"

.

. label variable cl_intcontent "Content of intervention"

. label define cl_intcontent 1 "General online hate speech/cyberhate" 2 "Right-wing extremism content" 3 "Left-wing extremism content" 4 "

> Islamist extremist content" 5 "Multiple" 6 "Other"

. label value cl_intcontent cl_intcontent

.

. label variable cl_intlocate "Location of intervention"

. label define cl_intlocate 1 "Website" 2 "Text messaging applications" 3 "Online social media platform" 4 "Other"

. label value cl_intlocate cl_intlocate

.

. label variable cl_intlocate_comment "Location of intervention (other)"

.

. label variable cl_platform "Social media platform"

. label define cl_platform 1 "Facebook" 2 "Instagram" 3 "TikTok" 4 "WhatsApp" 5 "Google" 6 "Snapchat" 7 "Twitter" 8 "4chan" 9 "Gab" 10 "Ot

> her (specify):" 11 "N/A"

. label value cl_platform cl_platform

.

. label variable cl_platform_comment "Social media platform (other)"

.

. label variable cl_txother "Other elements of this condition"

.

. label variable cl_comptype "Comparison condition"

. label define cl_comptype 1 "No exposure" 2 "Comparison exposure" 3 "Other (specify)"

. label value cl_comptype cl_comptype

.

. label variable cl_basediffsex "Baseline comparison of conditions (sex)"

. label define cl_basediffsex 1 "Statistically" 2 "Descriptively" 3 "Unknown"

. label value cl_basediffsex cl_basediffsex

.

. label variable cl_basediffrace "Baseline comparison of conditions (race)?"

. label define cl_basediffrace 1 "Statistically" 2 "Descriptively" 3 "Unknown"

. label value cl_basediffrace cl_basediffrace

.

. label variable cl_basediffage "Baseline comparison of conditions (age)"

. label define cl_basediffage 1 "Statistically" 2 "Descriptively" 3 "Unknown"

. label value cl_basediffage cl_basediffage

.

. label variable cl_selectionbias "RoB: baseline differences"

. label define cl_selectionbias 1 "Low risk" 2 "Some risk" 3 "High risk"

. label value cl_selectionbias cl_selectionbias

.

. label variable cl_attritiongen "RoB: gen attrition"

. label define cl_attritiongen 1 "Low risk" 2 "Some risk" 3 "High risk"

. label value cl_attritiongen cl_attritiongen

.

. label variable cl_attritiondiff "RoB: diff attrition"

. label define cl_attritiondiff 1 "Low risk" 2 "Some risk" 3 "High risk"

. label value cl_attritiondiff cl_attritiondiff

.

. *Dependent Variable level

.

. label variable dv_dvid "Outcome ID"

.

. label variable dv_dvlabel "DV label"

.

. label variable dv_elem "Elements reported"

. label define dv_elem 1 "Global dichotomy" 2 "Summed dichotomous" 3 "Frequency/rate" 4 "Scale" 5 "Event timing" 6 "Proportion" 7 "Rating

> of amount" 8 "Composite measure" 9 "Other (specify)" 10 "Unknown"

. label value dv_elem dv_elem

.

. label variable dv_type "Behavior measured"

. label define dv_type 1 "Content creation" 2 "Transmission of hate speech" 3 "Consumption of cyberhate" 4 "Collecting extremist content"

> 5 "Critics" 6 "Joiners" 7 "Other (specify)" 8 "Unknown"

. label value dv_type dv_type

.

. label variable dv_bias "RoB: person providing outcome data knows condition"

. label define dv_bias 1 "Low risk" 2 "Some risk" 3 "High risk"

. label value dv_bias dv_bias

.

.

. *Effect size level

.

. label variable es_compid "Comparison ID"

.

. label variable es_dvid "Outcome ID"

.

. label variable es_id "Effect size ID"

.

. label variable es_direct "Direction of effect"

. label define es_direct 1 "Favors treatment" 2 "Favors control" 3 "Neither (exactly equal)" 4 "Unknown"

. label value es_direct es_direct

.

. label variable es_etype "Type of effect size"

. label define es_etype 1 "Baseline" 2 "Post-test" 3 "Follow-up"

. label value es_etype es_etype

.

. label variable es_essig "Effect stat sig?"

. label define es_essig 1 "No" 2 "Yes" 3 "Unknown"

. label value es_essig es_essig

.

. label variable es_meantime "Mean timing of measurement"

.

. label variable es_mintime "Minimum timing of measurement"

.

. label variable es_maxtime "Maximum timing of measurement"

.

. label variable es_txn "Treatment sample size"

.

. label variable es_cgn "Comparison sample size"

.

. label variable es_mtx "Mean treatment group"

.

. label variable es_mcg "Mean comparison group"

.

. label variable es_madj "Means adjusted?"

. label define es_madj 1 "No" 2 "Yes" 3 "Unknown"

. label value es_madj es_madj

.

. label variable es_sdtx "SD treatment group"

.

. label variable es_sdcg "SD comparison group"

.

. label variable es_setx "SE treatment group"

.

. label variable es_secg "SE comparison group"

.

. *label variable es_txn "Treatment number successful"

.

. *label variable es_cgn "Comparison number successful"

.

. label variable es_txnf "Treatment number failures"

.

. label variable es_cgnf "Comparison number failures"

.

. label variable es_txpf "Treatment proportion"

.

. label variable es_cgpf "Comparison proportion"

.

. label variable es_padj "Frequencies/proportions adjusted?"

. label define es_padj 1 "No" 2 "Yes" 3 "Unknown"

. label value es_padj es_padj

.

. label variable es_lgor "Logistic regression coeff"

.

. label variable es_selgor "SE for logistic regression coeff"

.

. label variable es_olst "T-test/z-test for logistic regression coeff"

.

. label variable es_or "Odds ratio for treatment"

.

. label variable es_olsb "Unstandardized regression coeff"

.

. label variable es_olsbeta "Standard regression coeff"

.

. label variable es_olsse "SE of regression coeff"

.

. label variable es_sddv "SD for dependent variable"

.

. label variable es_t "t-test"

.

. label variable es_pfromt "p-value from t-test"

.

. label variable es_rpb "Correlation coeff point-biserial"

.

. label variable es_rphi "Correlation coeff phi"

.

. label variable es_chisq "Chi-square"

.

. label variable es_hand "SMD (computed by hand)"

.

. label variable es_handv "Variance for SMD (computed by hand)"

.

. label variable es_calc "Computed effect size"

.

. label variable es_calcse "Computed effect size SE"

.

. label variable es_page "Page number ES data"

.

.

. // Assigning missing values

.

. mvdecode _all, mv(-99=.a \ -98=.b \ -97=.c)

author: string variable ignored

cl_meanage: 10 missing values generated

cl_maxage: 3 missing values generated

cl_whites: 3 missing values generated

cl_blacksa~s: 3 missing values generated

cl_hispani~s: 3 missing values generated

cl_asians: 3 missing values generated

cl_mixedmu~e: 3 missing values generated

cl_other: string variable ignored

cl_inttype~t: string variable ignored

cl_intloca~t: string variable ignored

cl_platfor~t: string variable ignored

cl_txother: string variable ignored

dv_dvlabel: string variable ignored

dv_type_co~t: string variable ignored

es_meantime: 10 missing values generated

es_mintime: 10 missing values generated

es_maxtime: 10 missing values generated

es_mtx: 1 missing value generated

es_mcg: 1 missing value generated

es_sdtx: 1 missing value generated

es_sdcg: 1 missing value generated

es_setx: 1 missing value generated

es_secg: 1 missing value generated

es_txnf: 1 missing value generated

es_cgnf: 1 missing value generated

es_lgor: 1 missing value generated

es_selgor: 1 missing value generated

es_olst: 1 missing value generated

es_or: 1 missing value generated

es_olsb: 1 missing value generated

es_olsbeta: 1 missing value generated

es_olsse: 1 missing value generated

es_sddv: 1 missing value generated

es_t: 1 missing value generated

es_pfromt: 1 missing value generated

es_rpb: 1 missing value generated

es_rphi: 1 missing value generated

es_chisq: 1 missing value generated

es_hand: 1 missing value generated

es_handv: 1 missing value generated

es_calc: 1 missing value generated

es_calcse: 1 missing value generated

es_page: string variable ignored

.

.

.

.

. save hsdata

file hsdata.dta saved

. log close cleaning

name: cleaning

log: C:\Users\ajima\Dropbox\projects\hspeechSR\data\cleaning.log

log type: text

closed on: 2 Aug 2021, 11:23:59

------------------------------------------------------------------------------------------------------------------------------------------

------------------------------------------------------------------------------------------------------------------------------------------

name: desc

log: C:\Users\ajima\Dropbox\projects\hspeechSR\data\desc.log

log type: text

opened on: 2 Aug 2021, 11:24:06

. use hsdata.dta

.

. //Descriptives for study level

.

. tab sl_pubtype

Publication type | Freq. Percent Cum.

------------------------------------+-----------------------------------

Journal article (peer reviewed) | 3 30.00 30.00

Technical report | 7 70.00 100.00

------------------------------------+-----------------------------------

Total | 10 100.00

. tab sl_pubtype refid, m

| Reference ID

Publication type | Bodine-Ba Alvarez-B | Total

----------------------+----------------------+----------

Journal article (peer | 0 3 | 3

Technical report | 7 0 | 7

----------------------+----------------------+----------

Total | 7 3 | 10

.

. tab sl_language

Language |

type of |

study | Freq. Percent Cum.

------------+-----------------------------------

English | 10 100.00 100.00

------------+-----------------------------------

Total | 10 100.00

. tab sl_language refid, m

Language |

type of | Reference ID

study | Bodine-Ba Alvarez-B | Total

-----------+----------------------+----------

English | 7 3 | 10

-----------+----------------------+----------

Total | 7 3 | 10

.

. tab sl_location

Location of |

study | Freq. Percent Cum.

--------------+-----------------------------------

Europe | 3 30.00 30.00

Asia | 7 70.00 100.00

--------------+-----------------------------------

Total | 10 100.00

. tab sl_location refid, m

Location of | Reference ID

study | Bodine-Ba Alvarez-B | Total

--------------+----------------------+----------

Europe | 0 3 | 3

Asia | 7 0 | 7

--------------+----------------------+----------

Total | 7 3 | 10

.

. tab sl_datastart

Year data |

collection |

started | Freq. Percent Cum.

------------+-----------------------------------

2016 | 3 30.00 30.00

2019 | 7 70.00 100.00

------------+-----------------------------------

Total | 10 100.00

. tab sl_datastart refid, m

Year data |

collection | Reference ID

started | Bodine-Ba Alvarez-B | Total

-----------+----------------------+----------

2016 | 0 3 | 3

2019 | 7 0 | 7

-----------+----------------------+----------

Total | 7 3 | 10

.

. tab sl_dataend

Year data |

collection |

ended | Freq. Percent Cum.

------------+-----------------------------------

2016 | 3 30.00 30.00

2019 | 7 70.00 100.00

------------+-----------------------------------

Total | 10 100.00

. tab sl_dataend refid, m

Year data |

collection | Reference ID

ended | Bodine-Ba Alvarez-B | Total

-----------+----------------------+----------

2016 | 0 3 | 3

2019 | 7 0 | 7

-----------+----------------------+----------

Total | 7 3 | 10

.

. tab sl_inttype

Intervention |

type | Freq. Percent Cum.

---------------+-----------------------------------

Online only | 10 100.00 100.00

---------------+-----------------------------------

Total | 10 100.00

. tab sl_inttype refid, m

Intervention | Reference ID

type | Bodine-Ba Alvarez-B | Total

---------------+----------------------+----------

Online only | 7 3 | 10

---------------+----------------------+----------

Total | 7 3 | 10

.

. tab sl_rinvolve

Researcher |

involvement | Freq. Percent Cum.

---------------------+-----------------------------------

Researcher-initiated | 3 30.00 30.00

Online platform | 7 70.00 100.00

---------------------+-----------------------------------

Total | 10 100.00

. tab sl_rinvolve refid, m

Researcher | Reference ID

involvement | Bodine-Ba Alvarez-B | Total

---------------------+----------------------+----------

Researcher-initiated | 0 3 | 3

Online platform | 7 0 | 7

---------------------+----------------------+----------

Total | 7 3 | 10

.

. tab sl_funding

Externally |

funded? | Freq. Percent Cum.

------------+-----------------------------------

Yes | 10 100.00 100.00

------------+-----------------------------------

Total | 10 100.00

. tab sl_funding refid, m

Externally | Reference ID

funded? | Bodine-Ba Alvarez-B | Total

-----------+----------------------+----------

Yes | 7 3 | 10

-----------+----------------------+----------

Total | 7 3 | 10

.

. tab sl_uoa

Unit of assignment | Freq. Percent Cum.

-----------------------------+-----------------------------------

Individual | 10 100.00 100.00

-----------------------------+-----------------------------------

Total | 10 100.00

. tab sl_uoa refid, m

| Reference ID

Unit of assignment | Bodine-Ba Alvarez-B | Total

----------------------+----------------------+----------

Individual | 7 3 | 10

----------------------+----------------------+----------

Total | 7 3 | 10

.

. tab sl_method

Methods | Freq. Percent Cum.

--------------+-----------------------------------

Quantitative | 10 100.00 100.00

--------------+-----------------------------------

Total | 10 100.00

. tab sl_method refid, m

| Reference ID

Methods | Bodine-Ba Alvarez-B | Total

--------------+----------------------+----------

Quantitative | 7 3 | 10

--------------+----------------------+----------

Total | 7 3 | 10

.

. tab sl_assign

Subject assignment | Freq. Percent Cum.

----------------------------------------+-----------------------------------

Randomly w/o matching | 10 100.00 100.00

----------------------------------------+-----------------------------------

Total | 10 100.00

. tab sl_assign refid, m

| Reference ID

Subject assignment | Bodine-Ba Alvarez-B | Total

----------------------+----------------------+----------

Randomly w/o matching | 7 3 | 10

----------------------+----------------------+----------

Total | 7 3 | 10

.

. tab sl_rndinteg

Random |

assignment |

integrity | Freq. Percent Cum.

---------------+-----------------------------------

Maintained | 10 100.00 100.00

---------------+-----------------------------------

Total | 10 100.00

. tab sl_rndinteg refid, m

Random |

assignment | Reference ID

integrity | Bodine-Ba Alvarez-B | Total

---------------+----------------------+----------

Maintained | 7 3 | 10

---------------+----------------------+----------

Total | 7 3 | 10

.

. tab sl_selectrepb

Risk of |

selective |

outcome |

reporting? | Freq. Percent Cum.

------------+-----------------------------------

Low risk | 10 100.00 100.00

------------+-----------------------------------

Total | 10 100.00

. tab sl_selectrepb refid, m

Risk of |

selective |

outcome | Reference ID

reporting? | Bodine-Ba Alvarez-B | Total

-----------+----------------------+----------

Low risk | 7 3 | 10

-----------+----------------------+----------

Total | 7 3 | 10

.

.

. //Descriptives for comparison level

.

. tab cl_ctxn

Treatment |

group N | Freq. Percent Cum.

------------+-----------------------------------

42 | 1 10.00 10.00

45 | 1 10.00 20.00

46 | 1 10.00 30.00

475 | 7 70.00 100.00

------------+-----------------------------------

Total | 10 100.00

. tab cl_ctxn refid, m

Treatment | Reference ID

group N | Bodine-Ba Alvarez-B | Total

-----------+----------------------+----------

42 | 0 1 | 1

45 | 0 1 | 1

46 | 0 1 | 1

475 | 7 0 | 7

-----------+----------------------+----------

Total | 7 3 | 10

.

. tab cl_ccgn

Comparison |

group N | Freq. Percent Cum.

------------+-----------------------------------

47 | 3 30.00 30.00

465 | 7 70.00 100.00

------------+-----------------------------------

Total | 10 100.00

. tab cl_ccgn refid, m

Comparison | Reference ID

group N | Bodine-Ba Alvarez-B | Total

-----------+----------------------+----------

47 | 0 3 | 3

465 | 7 0 | 7

-----------+----------------------+----------

Total | 7 3 | 10

.

. tab cl_meanage

no observations

. tab cl_meanage refid, m

| Reference ID

Mean age | Bodine-Ba Alvarez-B | Total

-----------+----------------------+----------

.a | 7 0 | 7

.c | 0 3 | 3

-----------+----------------------+----------

Total | 7 3 | 10

.

. tab cl_minage

Youngest |

age | Freq. Percent Cum.

------------+-----------------------------------

18 | 10 100.00 100.00

------------+-----------------------------------

Total | 10 100.00

. tab cl_minage refid, m

Youngest | Reference ID

age | Bodine-Ba Alvarez-B | Total

-----------+----------------------+----------

18 | 7 3 | 10

-----------+----------------------+----------

Total | 7 3 | 10

.

. tab cl_maxage

Oldest age | Freq. Percent Cum.

------------+-----------------------------------

24 | 7 100.00 100.00

------------+-----------------------------------

Total | 7 100.00

. tab cl_maxage refid, m

| Reference ID

Oldest age | Bodine-Ba Alvarez-B | Total

-----------+----------------------+----------

24 | 7 0 | 7

.c | 0 3 | 3

-----------+----------------------+----------

Total | 7 3 | 10

.

. tab cl_sex

Sex |

distributio |

n | Freq. Percent Cum.

------------+-----------------------------------

51-75% | 7 70.00 70.00

26-50% | 3 30.00 100.00

------------+-----------------------------------

Total | 10 100.00

. tab cl_sex refid, m

Sex |

distributi | Reference ID

on | Bodine-Ba Alvarez-B | Total

-----------+----------------------+----------

51-75% | 7 0 | 7

26-50% | 0 3 | 3

-----------+----------------------+----------

Total | 7 3 | 10

.

. tab cl_whites

% Whites | Freq. Percent Cum.

------------+-----------------------------------

0 | 7 100.00 100.00

------------+-----------------------------------

Total | 7 100.00

. tab cl_whites refid, m

| Reference ID

% Whites | Bodine-Ba Alvarez-B | Total

-----------+----------------------+----------

0 | 7 0 | 7

.c | 0 3 | 3

-----------+----------------------+----------

Total | 7 3 | 10

.

. tab cl_blacksafricanscaribbeans

% |

Blacks/Afri |

cans/Caribb |

eans | Freq. Percent Cum.

------------+-----------------------------------

0 | 7 100.00 100.00

------------+-----------------------------------

Total | 7 100.00

. tab cl_blacksafricanscaribbeans refid, m

% |

Blacks/Afr |

icans/Cari | Reference ID

bbeans | Bodine-Ba Alvarez-B | Total

-----------+----------------------+----------

0 | 7 0 | 7

.c | 0 3 | 3

-----------+----------------------+----------

Total | 7 3 | 10

.

. tab cl_hispanicsnonwhites

% Hispanics |

(non-White) | Freq. Percent Cum.

------------+-----------------------------------

0 | 7 100.00 100.00

------------+-----------------------------------

Total | 7 100.00

. tab cl_hispanicsnonwhites refid, m

% |

Hispanics |

(non-White | Reference ID

) | Bodine-Ba Alvarez-B | Total

-----------+----------------------+----------

0 | 7 0 | 7

.c | 0 3 | 3

-----------+----------------------+----------

Total | 7 3 | 10

.

. tab cl_asians

% Asians | Freq. Percent Cum.

------------+-----------------------------------

0 | 7 100.00 100.00

------------+-----------------------------------

Total | 7 100.00

. tab cl_asians refid, m

| Reference ID

% Asians | Bodine-Ba Alvarez-B | Total

-----------+----------------------+----------

0 | 7 0 | 7

.c | 0 3 | 3

-----------+----------------------+----------

Total | 7 3 | 10

.

. tab cl_mixedmultiple

% |

mixed/multi |

ple ethnic |

groups | Freq. Percent Cum.

------------+-----------------------------------

0 | 7 100.00 100.00

------------+-----------------------------------

Total | 7 100.00

. tab cl_mixedmultiple refid, m

% |

mixed/mult |

iple |

ethnic | Reference ID

groups | Bodine-Ba Alvarez-B | Total

-----------+----------------------+----------

0 | 7 0 | 7

.c | 0 3 | 3

-----------+----------------------+----------

Total | 7 3 | 10

.

. tab cl_other

% other | Freq. Percent Cum.

------------+-----------------------------------

-97 | 3 30.00 30.00

80.1 Java | 7 70.00 100.00

------------+-----------------------------------

Total | 10 100.00

. tab cl_other refid, m

| Reference ID

% other | Bodine-Ba Alvarez-B | Total

-----------+----------------------+----------

-97 | 0 3 | 3

80.1 Java | 7 0 | 7

-----------+----------------------+----------

Total | 7 3 | 10

.

. tab cl_inttype

Type of intervention | Freq. Percent Cum.

----------------------------------+-----------------------------------

Responding to online hate | 1 10.00 10.00

Other | 9 90.00 100.00

----------------------------------+-----------------------------------

Total | 10 100.00

. tab cl_inttype refid, m

| Reference ID

Type of intervention | Bodine-Ba Alvarez-B | Total

----------------------+----------------------+----------

Responding to online | 0 1 | 1

Other | 7 2 | 9

----------------------+----------------------+----------

Total | 7 3 | 10

.

. tab cl_inttype_comment

Type of intervention |

(Other) | Freq. Percent Cum.

-------------------------+-----------------------------------

Deletion of hate content | 2 22.22 22.22

social media campaign | 7 77.78 100.00

-------------------------+-----------------------------------

Total | 9 100.00

. tab cl_inttype_comment refid, m

Type of intervention | Reference ID

(Other) | Bodine-Ba Alvarez-B | Total

----------------------+----------------------+----------

| 0 1 | 1

Deletion of hate co.. | 0 2 | 2

social media campaign | 7 0 | 7

----------------------+----------------------+----------

Total | 7 3 | 10

.

. tab cl_intcontent

Content of intervention | Freq. Percent Cum.

-------------------------------------+-----------------------------------

General online hate speech/cyberhate | 7 70.00 70.00

Multiple | 3 30.00 100.00

-------------------------------------+-----------------------------------

Total | 10 100.00

. tab cl_intcontent refid, m

Content of | Reference ID

intervention | Bodine-Ba Alvarez-B | Total

----------------------+----------------------+----------

General online hate s | 7 0 | 7

Multiple | 0 3 | 3

----------------------+----------------------+----------

Total | 7 3 | 10

.

. tab cl_intlocate

Location of intervention | Freq. Percent Cum.

-----------------------------+-----------------------------------

Online social media platform | 7 70.00 70.00

Other | 3 30.00 100.00

-----------------------------+-----------------------------------

Total | 10 100.00

. tab cl_intlocate refid, m

Location of | Reference ID

intervention | Bodine-Ba Alvarez-B | Total

----------------------+----------------------+----------

Online social media p | 7 0 | 7

Other | 0 3 | 3

----------------------+----------------------+----------

Total | 7 3 | 10

.

. tab cl_intlocate_comment

Location of intervention (other) | Freq. Percent Cum.

---------------------------------------+-----------------------------------

Online forum resembling internet forum | 3 100.00 100.00

---------------------------------------+-----------------------------------

Total | 3 100.00

. tab cl_intlocate_comment refid, m

Location of | Reference ID

intervention (other) | Bodine-Ba Alvarez-B | Total

----------------------+----------------------+----------

| 7 0 | 7

Online forum resemb.. | 0 3 | 3

----------------------+----------------------+----------

Total | 7 3 | 10

.

. tab cl_platform

Social media |

platform | Freq. Percent Cum.

-----------------+-----------------------------------

Other (specify): | 7 70.00 70.00

N/A | 3 30.00 100.00

-----------------+-----------------------------------

Total | 10 100.00

. tab cl_platform refid, m

Social media | Reference ID

platform | Bodine-Ba Alvarez-B | Total

-----------------+----------------------+----------

Other (specify): | 7 0 | 7

N/A | 0 3 | 3

-----------------+----------------------+----------

Total | 7 3 | 10

.

. tab cl_platform_comment

Social media platform (other) | Freq. Percent Cum.

--------------------------------------+-----------------------------------

Facebook, Instagram, Twitter, YouTube | 7 100.00 100.00

--------------------------------------+-----------------------------------

Total | 7 100.00

. tab cl_platform_comment refid, m

Social media platform | Reference ID

(other) | Bodine-Ba Alvarez-B | Total

----------------------+----------------------+----------

| 0 3 | 3

Facebook, Instagram.. | 7 0 | 7

----------------------+----------------------+----------

Total | 7 3 | 10

.

. tab cl_txother

Other elements of this condition | Freq. Percent Cum.

----------------------------------------+-----------------------------------

Highlights a descriptive norm against.. | 1 33.33 33.33

Highlights a descriptive norm against.. | 1 33.33 66.67

Replies highlighting the unacceptabil.. | 1 33.33 100.00

----------------------------------------+-----------------------------------

Total | 3 100.00

. tab cl_txother refid, m

Other elements of | Reference ID

this condition | Bodine-Ba Alvarez-B | Total

----------------------+----------------------+----------

| 7 0 | 7

Highlights a descri.. | 0 1 | 1

Highlights a descri.. | 0 1 | 1

Replies highlightin.. | 0 1 | 1

----------------------+----------------------+----------

Total | 7 3 | 10

.

. tab cl_comptype

Comparison |

condition | Freq. Percent Cum.

--------------------+-----------------------------------

Comparison exposure | 10 100.00 100.00

--------------------+-----------------------------------

Total | 10 100.00

. tab cl_comptype refid, m

Comparison | Reference ID

condition | Bodine-Ba Alvarez-B | Total

--------------------+----------------------+----------

Comparison exposure | 7 3 | 10

--------------------+----------------------+----------

Total | 7 3 | 10

.

. tab cl_basediffsex

Baseline |

comparison of |

conditions |

(sex) | Freq. Percent Cum.

--------------+-----------------------------------

Statistically | 7 70.00 70.00

Unknown | 3 30.00 100.00

--------------+-----------------------------------

Total | 10 100.00

. tab cl_basediffsex refid, m

Baseline |

comparison of |

conditions | Reference ID

(sex) | Bodine-Ba Alvarez-B | Total

--------------+----------------------+----------

Statistically | 7 0 | 7

Unknown | 0 3 | 3

--------------+----------------------+----------

Total | 7 3 | 10

.

. tab cl_basediffrace

Baseline |

comparison of |

conditions |

(race)? | Freq. Percent Cum.

--------------+-----------------------------------

Statistically | 7 70.00 70.00

Unknown | 3 30.00 100.00

--------------+-----------------------------------

Total | 10 100.00

. tab cl_basediffrace refid, m

Baseline |

comparison of |

conditions | Reference ID

(race)? | Bodine-Ba Alvarez-B | Total

--------------+----------------------+----------

Statistically | 7 0 | 7

Unknown | 0 3 | 3

--------------+----------------------+----------

Total | 7 3 | 10

.

. tab cl_basediffage

Baseline |

comparison of |

conditions |

(age) | Freq. Percent Cum.

--------------+-----------------------------------

Statistically | 7 70.00 70.00

Unknown | 3 30.00 100.00

--------------+-----------------------------------

Total | 10 100.00

. tab cl_basediffage refid, m

Baseline |

comparison of |

conditions | Reference ID

(age) | Bodine-Ba Alvarez-B | Total

--------------+----------------------+----------

Statistically | 7 0 | 7

Unknown | 0 3 | 3

--------------+----------------------+----------

Total | 7 3 | 10

.

. tab cl_selectionbias

RoB: |

baseline |

differences | Freq. Percent Cum.

------------+-----------------------------------

Low risk | 3 30.00 30.00

Some risk | 7 70.00 100.00

------------+-----------------------------------

Total | 10 100.00

. tab cl_selectionbias refid, m

RoB: |

baseline |

difference | Reference ID

s | Bodine-Ba Alvarez-B | Total

-----------+----------------------+----------

Low risk | 0 3 | 3

Some risk | 7 0 | 7

-----------+----------------------+----------

Total | 7 3 | 10

.

. tab cl_attritiongen

RoB: gen |

attrition | Freq. Percent Cum.

------------+-----------------------------------

Low risk | 3 30.00 30.00

High risk | 7 70.00 100.00

------------+-----------------------------------

Total | 10 100.00

. tab cl_attritiongen refid, m

RoB: gen | Reference ID

attrition | Bodine-Ba Alvarez-B | Total

-----------+----------------------+----------

Low risk | 0 3 | 3

High risk | 7 0 | 7

-----------+----------------------+----------

Total | 7 3 | 10

.

. tab cl_attritiondiff

RoB: diff |

attrition | Freq. Percent Cum.

------------+-----------------------------------

Low risk | 3 30.00 30.00

Some risk | 7 70.00 100.00

------------+-----------------------------------

Total | 10 100.00

. tab cl_attritiondiff refid, m

RoB: diff | Reference ID

attrition | Bodine-Ba Alvarez-B | Total

-----------+----------------------+----------

Low risk | 0 3 | 3

Some risk | 7 0 | 7

-----------+----------------------+----------

Total | 7 3 | 10

.

. save hsdata, replace

file hsdata.dta saved

. log close desc

name: desc

log: C:\Users\ajima\Dropbox\projects\hspeechSR\data\desc.log

log type: text

closed on: 2 Aug 2021, 11:24:06

-------------------------------------------------------------------------------------------------------------------------

name: meta

log: C:\Users\ajima\Dropbox\projects\hspeechSR\data\meta.log

log type: text

opened on: 4 Feb 2022, 09:34:43

. use hsdata.dta

.

.

. //Identify effect sizes with common outcome

. list author dv_dvlabel dv_type if dv_type == 1

+-------------------------------------------------------------------------+

| author dv_dvlabel dv_type |

|-------------------------------------------------------------------------|

1. | Bodine-Baron et al. (2020) Use social media Content creation |

8. | Alvarez-Benjumea & Winter (2018) Hate speech score Content creation |

9. | Alvarez-Benjumea & Winter (2018) Hate speech score Content creation |

10. | Alvarez-Benjumea & Winter (2018) Hate speech score Content creation |

+-------------------------------------------------------------------------+

.

. //Clarifying the different types of hate speech conditions for study 14921

. replace dv_dvlabel = "Hate speech score-counter" in 9

(1 real change made)

. replace dv_dvlabel = "Hate speech score-censord" in 10

(1 real change made)

. replace dv_dvlabel = "Hate speech score-excensord" in 8

(1 real change made)

.

. //compute effect sizes for standardized mean difference ...

.

. generate sdpooled = sqrt(((es_txn-1)*es_sdtx^2+(es_cgn-1)*es_sdcg^2)/(es_txn+es_cgn-2))

(5 missing values generated)

.

. generate d = (es_mtx-es_mcg)/sdpooled

(5 missing values generated)

.

. generate smdse = sqrt((es_txn+es_cgn)/(es_txn*es_cgn)+(d^2/(2*(es_txn+es_cgn))))

(5 missing values generated)

.

. generate w = 1/smdse^2

(5 missing values generated)

.

. //convert effect sizes to Hedges g and standard error

.

. generate g = d*(1-(3/(4*es_txn+es_cgn-9)))

(5 missing values generated)

. generate g_se = sqrt((es_txn+es_cgn)/(es_txn*es_cgn)+(g^2/(2*(es_txn+es_cgn))))

(5 missing values generated)

. generate g_w = 1/g_se^2

(5 missing values generated)

.

.

. //calculating adjusted effect size (Hedges g) for one study (14921) based on table 4 coefficients

.

. generate dadjusted = es_olsb/sdpooled

(7 missing values generated)

. generate gadjusted = dadjusted*(1-(3/(4*es_txn*es_cgn-9)))

(7 missing values generated)

. generate se_adjusted = gadjusted/(es_olsb/es_olsse)

(7 missing values generated)

. generate wadjusted = 1/se_adjusted^2

(7 missing values generated)

.

. // ... and logit transoformation for proportions for comparability to standardized mean difference; computes the logged

> odds ratio but converts to d by division by 1.83

.

. generate p_se = sqrt((1/(es_txn*es_txpf)) + (1/(es_txn*(1-es_txpf)))+ ///

> (1/(es_cgn*es_cgpf)) + (1/(es_cgn*(1-es_cgpf))))/1.83

(5 missing values generated)

.

. generate p_es = (ln(es_txpf*(1-es_cgpf)/(es_cgpf*(1-es_txpf))))/1.83

(5 missing values generated)

.

. generate w_pes = 1/p_se^2

(5 missing values generated)

.

. //generate and assign adjusted g and p_es to 'es' for meta set

.

. generate esg = gadjusted in 8/10

(7 missing values generated)

. replace esg = p_es in 1

(1 real change made)

.

. generate gse = se_adjusted in 8/10

(7 missing values generated)

. replace gse = p_se in 1

(1 real change made)

.

. generate gw = wadjusted in 8/10

(7 missing values generated)

. replace gw = w_pes in 1

(1 real change made)

.

. //Single effect sizes for study 14921

. list author dv_dvlabel gadjusted se_adjusted wadjusted if refid==14921, compress nol

+--------------------------------------------------------------------------------------------------+

| author dv_dvlabel gadjusted se_adj~d wadjus~d |

|--------------------------------------------------------------------------------------------------|

8. | Alvarez-Benjumea & Winter (2018) Hate speech score-excensord -.3144675 .125787 63.20164 |

9. | Alvarez-Benjumea & Winter (2018) Hate speech score-counter -.1093548 .124977 64.02361 |

10. | Alvarez-Benjumea & Winter (2018) Hate speech score-censord -.3287296 .1264345 62.55602 |

+--------------------------------------------------------------------------------------------------+

.

. //Table of all effect sizes

. list author dv_dvlabel esg gse gw if gse !=., compress nol

+--------------------------------------------------------------------------------------------------+

| author dv_dvlabel esg gse gw |

|--------------------------------------------------------------------------------------------------|

1. | Bodine-Baron et al. (2020) Use social media -.167701 .1480438 45.62674 |

8. | Alvarez-Benjumea & Winter (2018) Hate speech score-excensord -.3144675 .125787 63.20164 |

9. | Alvarez-Benjumea & Winter (2018) Hate speech score-counter -.1093548 .124977 64.02361 |

10. | Alvarez-Benjumea & Winter (2018) Hate speech score-censord -.3287296 .1264345 62.55602 |

+--------------------------------------------------------------------------------------------------+

.

. //declare meta-analysis data with pre-computed effect sizes. Model: random effects, method: REML

.

. meta set esg gse, studylabel(author)

(6 missing values generated)

Meta-analysis setting information

Study information

No. of studies: 4

Study label: author

Study size: N/A

Effect size

Type: <generic>

Label: Effect size

Variable: esg

Precision

Std. err.: gse

CI: [_meta_cil, _meta_ciu]

CI level: 95%

Model and method

Model: Random effects

Method: REML

.

. // meta-analysis for the effect sizes in observation 1 (study 13715) and observation 9 (study 14921)

. meta summarize if _meta_id ==1 | _meta_id ==9

Effect-size label: Effect size

Effect size: esg

Std. err.: gse

Study label: author

Meta-analysis summary Number of studies = 2

Random-effects model Heterogeneity:

Method: REML tau2 = 0.0000

I2 (%) = 0.00

H2 = 1.00

-----------------------------------------------------------------------------------

Study | Effect size [95% conf. interval] % weight

---------------------------------+-------------------------------------------------

Bodine-Baron et al. (2020) | -0.168 -0.458 0.122 41.61

Alvarez-Benjumea & Winter (2018) | -0.109 -0.354 0.136 58.39

---------------------------------+-------------------------------------------------

theta | -0.134 -0.321 0.054

-----------------------------------------------------------------------------------

Test of theta = 0: z = -1.40 Prob > |z| = 0.1617

Test of homogeneity: Q = chi2(1) = 0.09 Prob > Q = 0.7633

.

. //Produce forest plot for meta-analysis

.

. meta forestplot if _meta_id ==1 | _meta_id ==9

Effect-size label: Effect size

Effect size: esg

Std. err.: gse

Study label: author

.

. save hsdata, replace

file hsdata.dta saved

. log close meta

name: meta

log: C:\Users\ajima\Dropbox\projects\hspeechSR\data\meta.log

log type: text

closed on: 4 Feb 2022, 09:34:44

-------------------------------------------------------------------------------------------------------------------------
